# A single‐center cohort study of patients with hereditary spherocytosis in Central Europe reveals a high frequency of novel disease‐causing genotypes

**DOI:** 10.1002/hem3.31

**Published:** 2024-01-26

**Authors:** Leo Kager, Raúl Jimenez‐Heredia, Petra Zeitlhofer, Wolfgang Novak, Sebastian K. Eder, Anna Segarra‐Roca, Alexandra Frohne, Karin Nebral, Matthias Haimel, René Geyeregger, Katharina Roetzer‐Londgin, Oskar A. Haas, Kaan Boztug

**Affiliations:** ^1^ St. Anna Children's Hospital Medical University of Vienna Vienna Austria; ^2^ St. Anna Children's Cancer Research Institute (CCRI) Vienna Austria; ^3^ Ludwig Boltzmann Institute for Rare and Undiagnosed Diseases Vienna Austria; ^4^ Department of Pediatrics and Adolescent Medicine Medical University of Vienna Vienna Austria; ^5^ Labdia Labordiagnostik Vienna Austria; ^6^ CeMM Research Center for Molecular Medicine of the Austrian Academy of Sciences Vienna Austria

Hereditary spherocytosis (HS) is the most common red blood cell (RBC) cytoskeleton disorder and the most frequent cause of chronic hemolytic anemia (CHA) in Northern European ancestries.^1^ HS is phenotypically and genetically heterogeneous, caused by mutations that alter the expression and/or structure of transmembrane, adapter, or spectrin‐based cytoskeletal proteins essential for RBC stability, shape, and function.^1^ These defects lead to the formation of spheroidal, fragile RBCs that are selectively trapped in the spleen, resulting in CHA, jaundice, gallstone formation, and splenomegaly.^2^ Clinical HS phenotypes range from subclinical to transfusion‐dependent severe CHA. Total or near‐total splenectomies are curative for severe HS.^2^

HS‐causing defects comprise *ANK1* (encoding ankyrin‐1), *SPTB* (encoding β‐spectrin), *SPTA1* (encoding α‐spectrin), *SLC4A1* (encoding band 3), and *EPB42* (encoding protein 4.2) mutations. HS occurs in all ethnic groups, with differences in overall prevalence and genetic subtypes.^2^, ^3^

Next‐generation sequencing (NGS) has helped elucidate pathogenic variants underlying HS, the complexity of inheritance modes, and genotype–phenotype correlations in different ancestries including Western Europe,^4^ North America,^5^ South America,^6^ South Asia,^7^ and East Asia.^3^, ^8^ Moreover, better insights into the structures and interactions of key RBC protein complexes were recently provided.^9^

Here, we report the results of the first Central European single‐center study including 113 individuals from 35 families (HS patients, *n* = 69 including 35 index patients and 34 affected family members; unaffected first‐degree relatives, *n* = 44). Ancestry was Central European in 58/69 patients (86%) and Eastern Turkey (*n* = 5), Palestine region (*n* = 4), Iran (*n* = 1), and Chechnya (*n* = 1) (Supporting Information S1: Tables S1 and S2). Analyses on patients and both parents were performed in 30/35 (86%) families. Details of diagnostics, clinics, genetic analyses, case reports, and summaries on identified genetic variants are provided in the Supporting Information.

In the index group, we identified 34 causative variants in 34/35 patients (97%). Deleterious variants in *ANK1* were found in 16/35 (46%), in *SLC4A1* in 5/35 (14%), in *SPTB* in 11/35 (31%) patients, and in *SPTA1* in 2/35 (6%) (Table 1 and Supporting Information S1: Figure S25). In one patient (3%), we were unable to identify the genetic cause of HS unequivocally (Supporting Information S1: Patient P35). Details on the localization of the identified variants in the respective proteins are found in Figure 1. Combined annotation‐dependent depletion (CADD) versus minor allele frequency (MAF) plots and gDNA variant localization, are found in Supporting Information S1: Figures S6/S13/S20/S23.

**Table 1:** Description of the variants in HS genes in 34/35 patients of the index group in whom the genetic cause was unequivocally identified.

Patient ID	Gene	gDNA (Hg19)^a^	cDNA change^b^	Protein change^c^	Status	Mutation type	Inheritance	MAF GnomAD^d^	CADD score (v1.3)^e^	LELY^f^	αLEPRA^g^	PMID^h^	Pathogenicity^i^
1	*ANK1*	Chr8:41615608del	c.76del	p.Asp26ThrfsTer11	Het	Frameshift	AD^j^	NA	35	WT	WT	‐	Pathogenic
2	*ANK1*	Chr8:41584836G>A	c.358C>T	p.Gln120Ter	Het	Stop gain	AD^j^	NA	38	WT	WT	29797310^k^	Pathogenic
3	*ANK1*	Chr8:41575217del	c.1211del	p.Gly404AlafsTer2	Het	Frameshift	AD^j^, ^l^	NA	35	Het	WT	‐	Pathogenic
4	*ANK1*	Chr8:41573376C>T	c.1405−9G>A	p.Asp469GlyfsTer21	Het	Intronic	AD^j^, ^l^	NA	2.1	WT	WT	32436265	Pathogenic
5	*ANK1*	Chr8:41573376C>T	c.1405−9G>A	p.Asp469GlyfsTer21	Het	Intronic	De novo^m^	NA	2.1	Het	WT	32436265	Pathogenic
6	*ANK1*	Chr8:41571709del	c.1765del	p.Leu589PhefsTer48	Het	Frameshift	AD^j^	NA	35	WT	WT	‐	Pathogenic
7	*ANK1*	Chr8:41571689_41571701del	c.1773_1785del	p.Gly592ThrfsTer41	Het	Frameshift	AD^j^, ^l^	NA	36	Het	WT	29797310^k^	Pathogenic
8	*ANK1*	Chr8:41547745‐41552901del	‐	‐	Het	CNV	AD^j^	NA	NA	Het	WT	‐	Pathogenic
9	*ANK1*	Chr8:41552797C>A	c.3013G>T	p.Glu1005Ter	Het	Stop gain	AD^j^	NA	42	Het	WT	‐	Pathogenic
10	*ANK1*	Chr8:41550351del	c.3675del	p.Phe1225LeufsTer24	Het	Frameshift	AD^j^	NA	25.8	WT	WT	‐	Pathogenic
11	*ANK1*	Chr8:41550259del	c.3766del	p.Leu1256CysfsTer6	Het	Frameshift	De novo^m^	NA	35	Het	WT	‐	Pathogenic
12	*ANK1*	Chr8:41547849G>A	c.4000C>T	p.Arg1334Ter	Het	Stop gain	AD^j^	NA	50	Het	WT	31400153	Pathogenic
13	*ANK1*	Chr8:41546049del	c.4164del	p.Leu1389SerfsTer17	Het	Frameshift	Unknown^n^	NA	22.8	Het	WT	‐	Pathogenic
14	*ANK1*	Chr8:41543672_41543675del	c.4387_4390del	p.Asn1463TrpfsTer17	Het	Frameshift	AD^j^	NA	35	WT	WT	29797310^k^	Pathogenic
15	*ANK1*	Chr8:41542137G>A	c.4462C>T	p.Arg1488Ter	Het	Stop gain	De novo^m^	NA	36	Het	WT	12899723	Pathogenic
16	*ANK1*	Chr8:41402861_41535706del	‐	‐	Het	CNV	De novo^m^	NA	NA	Het	WT	‐	Pathogenic
17	*SLC4A1*	Chr17:42336589del	c.823del	p.His275ThrfsTer22	Het	Frameshift	AD^j^	NA	17.3	WT	WT	‐	Pathogenic
18	*SLC4A1*	Chr17:42335366C>G	c.1270G>C	p.Gly424Arg	Het	Missense	AD^j^	NA	33	Het	WT	‐	Likely pathogenic
19	*SLC4A1*	Chr17:42335028G>T	c.1430C>A	p.Ser477Ter	Hom	Stop gain	AR	NA	37	Het	WT	27718309^k^	Pathogenic
20	*SLC4A1*	Chr17:42334876G>A	c.1468C>T	p.Arg490Cys	Het	Missense	AD^j^	0.00003187	26.6	WT	WT	9233560	Pathogenic
21	*SLC4A1*	Chr17:42330512C>T	c.2285G>A	p.Ser762Asn	Het	Missense	AD^n^	NA	26.6	WT	WT	‐	Likely pathogenic
22	*SPTB*	Chr14:65271803G>A	c.154C>T	p.Arg52Trp	Het	Missense	AD^j^	NA	34	WT	WT	36035481	Likely pathogenic
23	*SPTB*	Chr14:1465263352dup	c.1264dup	p.Gln422ProfsTer7	Het	Frameshift	AD^j^	NA	35	WT	WT	‐	Pathogenic
24	*SPTB*	Chr14:65260587T>C	c.1796−2A>G	‐	Het	Splicing	De novo^m^	NA	23.3	WT	WT	‐	Pathogenic
25	*SPTB*	Chr14:65260469G>A	c.1912C>T	p.Arg638Ter	Het	Stop gain	Unknown^n^	NA	36	WT	WT	31723846	Pathogenic
26	*SPTB*	Chr14:65260425C>T	c.1956G>A	p.Trp652Ter	Het	Stop gain	Unknown^n^	NA	39	Het	WT	29505016	Pathogenic
27	*SPTB*	Chr14:65246611dup	c.4309dup	p.Glu1437GlyfsTer54	Het	Frameshift	AD^j^	NA	35	Het	WT	29797310^k^	Pathogenic
28	*SPTB*	Chr14:65240046G>T	c.5070C>A	p.Tyr1690Ter	Het	Stop gain	AD^j^	NA	39	Het	WT	‐	Pathogenic
29	*SPTB*	Chr14:65240011A>G	c.5105T>C	p.Leu1702Pro	Het	Missense	AD^j^, ^l^	NA	25.8	WT	WT	‐	Likely pathogenic
30	*SPTB*	Chr14:65239951_65239952del	c.5165_5166del	p.Phe1722Ter	Het	Frameshift	AD^j^	NA	35	Het	WT	33761640	Pathogenic
31	*SPTB*	Chr14:6523581_65235813delinsGAA	c.5961_5964delinsTTC	p.Met1988SerfsTer7	Het	Frameshift	Unknown^n^	NA	27.5	WT	WT	29797310^k^	Pathogenic
32	*SPTB*	Chr14:65235797_65235798del	c.5979_5980del	p.Glu1993AspfsTer3	Het	Frameshift	AD^j^	NA	36	WT	WT	‐	Pathogenic
33	*SPTA1*	Chr1:158624457G>A	c.2980C>T	p.Arg994Ter	Comp	Stop gain	AR	0.000004009	38	WT	HET	‐	Pathogenic
		Chr1:158613314G>A	c.4339−99C>T	p.Lys1449ValfsTer27	Het	Intronic		0.004543	8.7			8941647	Pathogenic
34	*SPTA1*	Chr1:158647479C>T	c.957+1G>A	‐	Comp	Splicing	AR	NA	26.4	HET	HET	‐	Pathogenic
		Chr1:158613314G>A	c.4339−99C>T	p.Lys1449ValfsTer27	Het	Intronic		0.004543	8.7			8941647	Pathogenic

Abbreviations: AD, autosomal dominant; *ANK1*, ankyrin 1; AR, autosomal recessive; CADD, combined annotation dependent depletion; cDNA, complementary DNA; CNV, copy number variants; gDNA, genomic DNA; Het, heterozygous; Hom, homozygous; HS, hereditary spherocytosis; MAF, minor allele frequency; NA, not annotated; *SLC4A1*, solute carrier family 4 member 1; *SPTA1*, spectrin alpha, erythrocytic 1; *SPTB*, spectrin beta, erythrocytic; WES, whole‐exome sequencing, WT, wild type.

^a^ Genomic coordinates and variant description for the genome assembly GRCh37 (Hg19) (https://grch37.ensembl.org/index.html).

^b^ The Human Genome Variation Society coding sequence name (HGVSc [cDNA]) (https://www.hgvs.org/).

^c^ The Human Genome Variation Society protein sequence name (HGVSc [protein]).

^d^ MAF provided by the Genome Aggregation Database (https://gnomad.broadinstitute.org/).

^e^ Prediction program: CADD v1.3 (http://cadd.gs.washington.edu/; https://pubmed.ncbi.nlm.nih.gov/24487276/).

^f^ LELY, low expression allele Lyon (αLELY), which is composed of two in cis variants in *SPTA1* (c.5572C>G and c.6531−12C) that cause strongly reduced amounts of α‐spectrin (https://pubmed.ncbi.nlm.nih.gov/8486776/).

^g^ αLEPRA (Low Expression PRAgue) is a deep intronic SPTA1 mutation (c.4339−99C>T). Positioned at ‐99 of intron 30, it activates an alternative acceptor splice site at position ‐70 of the same intron (https://www.ncbi.nlm.nih.gov/pmc/articles/PMC507680/).

^h^ PubMed identifier of the first report of the variant: yet already described variants.

^i^ According to the American College of Medical Genetics and Genomics/Association for Molecular Pathology (ACMG‐AMP) criteria (https://pubmed.ncbi.nlm.nih.gov/25741868/).

^j^ Both parents were tested and one carried the pathogenic variant and had HS phenotype (for details on the affected relatives, see Supporting Information S1: Table S2), the other carried neither the variant nor had HS phenotype.

^k^ Already published by our group, more details are provided in the Supporting Information.

^l^ Siblings with HS phenotypes were tested; they carried the pathogenic variant.

^m^ Both parents were tested and none carried the pathogenic variant and none had HS phenotype.

^n^ Details are provided in the Supporting Information.

**Figure 1:**
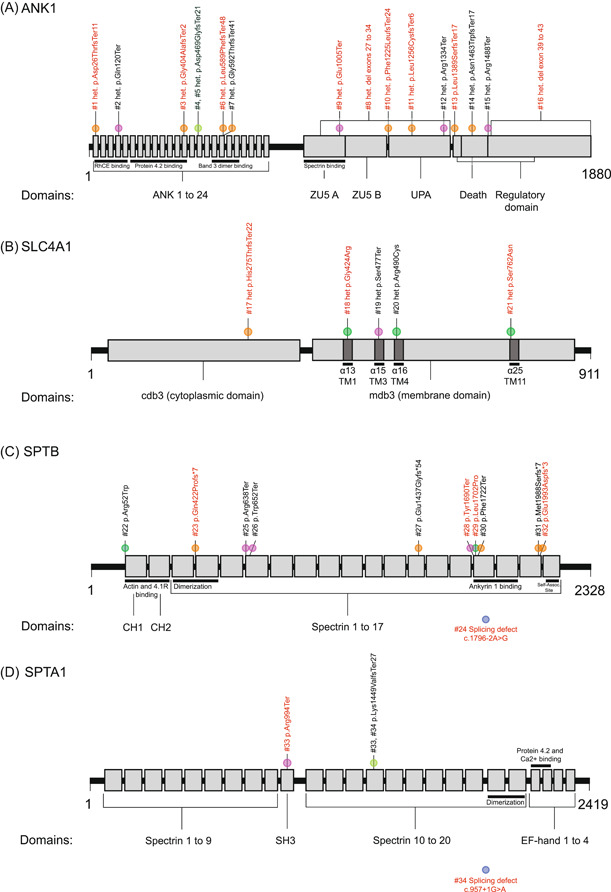
Two‐dimensional protein representation of (A) *ANK1*, (B) *SLC4A1*, (C) *SPTB*, and (D) *SPTA1*. In red, are newly described mutations. In black, are mutations previously reported in the literature. The colors of the circles define the type of mutation: orange for frameshifts, pink for nonsense, light green for intronic, dark green for missense, and blue for splicing mutations.

Of the 34 identified variants, 19 (56%) were not previously reported. Six variants were described previously by us (Table 1), and two of these were subsequently reported in other populations (Supporting Information S1: Families F2/F14). The remaining nine variants were first published elsewhere (Table 1 and Supporting Information S1: Tables S4–S6). From the 34 index patients with pathogenic (*n* = 30) or likely pathogenic (*n* = 4) variants, 22 had AD inherited heterozygous variants (64.5%), five had AD de novo heterozygous variants (14.5%), three had autosomal recessive inheritance (one homozygous *SLC4A1*, two compound heterozygous *SPTA1*, 9%), and four had variants with unknown inheritance patterns (12%) (Table 1).

Most identified variants were high impact (*n* = 27/34; 79%) including frameshift (*n* = 14; 41%), nonsense (*n* = 9, 26%), copy number variants (CNVs, *n* = 2; 6%), and splice‐site variants (*n* = 2; 6%). The remaining were missense (*n* = 5; 15%) and intronic variants (*n* = 2; 6%) (Table 1 and Supporting Information S1: Figure S25). We could demonstrate that the intronic variant in *ANK1* (c.1405−9 G>A) generated a new splice–acceptor site resulting in a frameshift and premature stop (Supporting Information S1: Figure S2 and Families F4/F5), as recently reported by others.^10^ Truncating variants were common in *ANK1, SPTB*, and *SPTA1* (Supporting Information S1: Figures S5/S17/S22). In *SLC4A1*, 60% of variants were missense (Supporting Information S1: Figures S11 and S12); and two patients with *SPTB‐*associated HS carried missense variants (Supporting Information S1: Figures S17 and S18). One heterozygous AD‐inherited CNV in *ANK1* (interstitial deletion of exons 27–33) was discovered in Family 8 (Figure 1A and Supporting Information S1: Family F8). The other de novo CNV (132.85 kb large deletion) in Patient P16 affects a total of six contiguous genes (*LOC102723729*, *GPAT4* [OMIM #608143], *NKX6‐3* [OMIM #610772], *ANK1* exons 39–43 [C‐terminal region], *MIR486‐1*, and *MIR486‐2*) (Figure 1A and Supporting Information S1: Family F16 and Figures S3 and S4). The 34 affected family members carried the same causative variants as their pediatric index‐group relatives (Supporting Information S1: Families F1–F34).

In 4/34 index patients (12%) with known cause of disease, ≥2 putative pathogenic variants in HS candidate genes were identified (Supporting Information S1: Families F18/F24/F28/F33 and Figures S9/S15/S16/S21).

The low‐expression allele Lyon (αLELY), composed of two in cis *SPTA1* variants (c.5572C>G and c.6531−12C>T) causing strongly reduced amounts of α‐spectrin, was analyzed in the index group, with 18/35 (51%) patients heterozygous for this allele (Table 1 and Supporting Information S1: Families F1–F35).^11^ The deep intronic “Low Expression allele PRAgue” (α‐LEPRA), which enhances an alternative acceptor splice site leading to nonsense‐mediated decay,^12^ was analyzed in all index group patients. Two were identified to carry heterozygous α‐LEPRA variants in trans to other pathogenic *SPTA1* variants (Table 1 and Supporting Information S1: Families F33 and F34). Overall, causative variants were identified in 68/69 patients (98.5%) and 32/69 (46%) had causative variants in *ANK1*, 19/69 in *SPTB* (28%), 14/69 in *SLC4A1* (20%), and 3/69 (4%) in *SPTA1*.

Clinical characteristics for all 69 patients are summarized in Supporting Information S1: Tables S1a–e (index group) and S2a–d (affected family members). Data for grading disease severity before splenectomy were available for all 35 index patients and 21 affected family members. Five of the 56 (9%) patients had severe phenotypes (*ANK1*, *n* = 2; *SPTB*, *n* = 2; homozygous *SLC4A1*, *n* = 1). Moderate phenotypes were observed in 34/56 (61%) patients (*ANK1*, *n* = 17; *SPTB*, *n* = 10; *SLC4A1*, *n* = 3; compound heterozygous *SPTA1*, *n* = 3; causative variants not unequivocally identified, *n* = 1) and mild phenotypes in 17/56 (30%) patients (*SLC4A1*, *n* = 10; *SPTB*, *n* = 5; *ANK1*, *n* = 2). There were milder phenotypes in patients with heterozygous *SCL4A1* variants, and eosin 5‐maleimide binding was highest compared to the other variant cohorts (Supporting Information S1: Table S3 and Figure S1). Hemoglobin levels were significantly higher in patients with heterozygous *SLC4A1* variants compared to patients with *ANK1* variants (*p*‐adjusted <0.001) and compound heterozygous *SPTA1* variants (*p*‐adjusted <0.05) (Supporting Information S1: Table S3 and Figure S1). Of note, hemoglobin levels were higher in patients with *SPTB* missense variants compared to *SPTB* stop gain variants (*p*‐adjusted <0.05) (Supporting Information S1: Figure S19). However, the number of patients was too low to allow the establishment of robust phenotype correlation with different types of variants in HS candidate genes in our cohort (Supporting Information S1: Figures S7/S8 [*ANK1*], S14 [*SLC4A1*], and S19 [*SPTB*]).

Compared to other studies, we performed all analyses in our hospital and research institute including all available first‐degree family members. This allowed us to identify causative variants in 98.5% of patients; and this accuracy is higher than previously reported (e.g., in studies from Western Europe [89%], North America [97%],^5^ and East Asia [85%]^8^).

We found variants in *ANK1* as the most common cause of HS (46%), in line with results from North America (49%)^5^ and South Asia (53.2%).^7^ Lower frequencies of causative *ANK1* variants were reported in cohorts from Western Europe (27%)^4^ and East Asia (29%).^8^ Stop‐gain and frameshift mutations were found in 75% of our patients with *ANK1* variants, similar to results from East^3^ and South Asia.^7^ Interestingly, the intronic variant leading to a frameshift (*ANK1*, c.1405−9G>A) appears common and was described in different ancestries (Table 1 and Supporting Information S1: Table S4).

Variants in *SPTB* were identified in 31% of index patients, similar in frequency to North America (33%),^5^ but higher than in Western Europeans (20%).^4^ The highest incidence of causative *SPTB* variants was found in Asian HS cohorts.^3^, ^7^ Of note, all patients carrying causative variants in *SPTB* in our study had Central European ancestry. Similar to results from Asia,^7^ truncating variants were found in most of our *SPTB* patients. In most populations, <20% of causative defects in *SPTB* are missense variants.^5^, ^7^

In 14% of our index patients, causative variants in *SLC4A1* were identified, as observed in West European (14%)^4^ and North American (13%)^5^ cohorts. Missense variants were the most prevalent genomic aberrations (60%), corroborating previous findings.^3^, ^4^ The patient with the most severe phenotype in our cohort carries a homozygous variant in *SLC4A1* (Band3 null^Vienna^) and we provide an update on the clinical course since the first publication^13^ (Supporting Information S1: Family F19 and Figure S10).

A Western European study identified variants in *SPTA1* as the most common cause (36%) of HS.^4^ Like in other series (South Asia [6.4%],^7^ East Asia [9%],^3^ North America [5%]^5^), 6% of our index patients carried causative *SPTA1* variants. Interestingly, all carried the low expression α‐LEPRA allele in trans to a high impact *SPTA1* variant, and have moderate HS phenotypes.

Genotype–phenotype correlations have corroborated that patients who carry heterozygous variants in *ANK1* and *SPTB* (more often high‐impact) are phenotypically similar, and patients with heterozygous *SLC4A1* variants (more often missense) have milder hematological phenotypes,^4^, ^5^, ^14^ with considerable phenotypical variability in the different genetic subcohorts.

Collectively, we provide the first report on the mutational spectrum of HS in a single center in Central Europe. Family analyses, in‐house standardized laboratory, and clinical investigations, coupled with NGS analyses, allowed the identification of the genetic cause with high accuracy and confidence in 98.5% of patients and the discovery of unusual causative variants, like deep intronic, splicing, CNVs, and a homozygous pathogenic variant in *SLC4A1*.

## AUTHOR CONTRIBUTIONS

Leo Kager and Kaan Boztug designed the research study. Raúl Jimenez‐Heredia, Anna Segarra‐Roca, Alexandra Frohne, Karin Nebral, and Petra Zeitlhofer performed the research. Matthias Haimel and Raúl Jimenez‐Heredia analyzed the NGS data. Wolfgang Novak and Sebastian K. Eder performed the statistical analysis. Leo Kager, Raúl Jimenez‐Heredia, and Kaan Boztug wrote the paper. René Geyeregger, Katharina Roetzer‐Londgin, and Oskar A. Haas critically reviewed the paper.

## CONFLICT OF INTEREST STATEMENT

Leo Kager reports acting on the advisory board of Agios, Amgen, Bayer, and Novartis (unrelated to this study). The remaining authors declare no conflict of interest.

## FUNDING

This study was supported through intramural funding of the Ludwig Boltzmann Institute for Rare and Undiagnosed Diseases (LBI‐RUD) Vienna, and by a charitable donation of the Kapsch group (www.kapsch.net/kapschgroup) to O. A. H.

## Supporting information

Supporting information.

## Data Availability

The data that supports the findings of this study are available in the Supporting Information of this article.
