# Supplementary material for: A single‐center cohort study of patients with hereditary spherocytosis in Central Europe reveals a high frequency of novel disease‐causing genotypes
Source: Hemasphere. 2024 Jan 26;8(1):e31. doi: 10.1002/hem3.31 (PMC10878193; doi:10.1002/hem3.31)
Supplement: Supplementary file 1 — Supporting information. [file HEM3-8-e31-s001.docx]

**Supporting information to**

**A single-centre cohort study of patients with hereditary spherocytosis in Central Europe reveals a high frequency of novel disease-causing genotypes**

Author list: See Title page in the submission.

**Supplemental materials and methods** 4-6

## Supplemental clinical and laboratory results

Index patients: Table S1a (ANK1) 7-8

Table S1b (SLC4A1) 8

Table S1c (SPTB) 9

Table S1d (SPTA1) 10

Table S1e (no unequivocal causative variants identified) 10

Affected family members: Table S2a (ANK1) 11-12

Table S2b (SLC4A1) 13

Table S2c (SPTB) 14

Table S2d (SPTA1) 15

Table S3 16

Figure S1 16

## Supplemental genetic results

General information 17-19

## Case reports: Index patients (P) and their families (F) with causative variants in *ANK1*

## Families F1-F16 20-38

Figure S2: Splice site predicted effect of the intronic variants c.1405-9G>A 25

Figure S3: SNP/CGH array analysis of P16 37

Figure S4: MRI of the abdomen of patient 16 38

Summary *ANK1* variants: Figure S5: The 15 causative variants and the types of variation 39

Figure S6A and S6B: gDNA/cDNA variant representation and CADD/MAF graph 40

Figure S7: HS phenotypes and types of variants 41

Figure S8: HS phenotypes and localization of variants 42

Table S4: *ANK1* variants described ≥ 2 families (our study) 43

**Case reports: Index patients (P) and their families (F) with causative variants in *SLC4A1***

Families F17-F21 44-50

Figure S9: Segregation analysis in P18 46

Figure S10: Peripheral blood smear of P19 after splenectomy 48

Summary *SLC4A1* variants: Figure S11: The 5 causative variants and the types of variation 51

Figure S12: 3D model of band 3 and 3 missense variants 52

Figure S13A and S13B: gDNA/cDNA variant representation and CADD/MAF graph 53

Figure S14: HS phenotypes and types of variants 54

Table S5: *SLC4A1* variants described ≥ 2 families (our study) 54

**Case reports: Index patients (P) and their families (F) with causative variants in *SPTB***

Families F22-F32 55-67

Figure S15: *SPTB* splice acceptor variant segregation in F24 58

Figure S16: *SPTB* variants segregation in F28 63

Summary *SPTB* variants: Figure S17: The 11 causative variants and the types of variation 68

Figure S18: 3D model of beta-spectrin and two missense variants 69

Figure S19: HS phenotypes and types of SPTB variants 69

Figure S20A and S20B: gDNA/cDNA variant representation and CADD/MAF graph 70

Table S6: SPTB variants described ≥ 2 families (our study) 71

**Case reports: Index patients (P) and their families (F) with causative variants in *SPTA1***

Families F33-F34 72-74

Figure S21: Variant segregation in F33 73

Summary *SPTA1* variants: Figure S22: The 3 causative variants and the types of variation 75

Figure S23A and S23B: gDNA/cDNA variant representation and CADD/MAF graph 76

**Case reports: Index patient (P) and his family (F) with no unequivocal causative candidate variants identified**

Family F35 77-78

Figure S24: Variant segregation in F35 78

**Summary causative variants:**

Figure S25: Distribution of variants in HS candidate genes in the index group 79

**Supporting references** 80-85

# Supplemental Materials and Methods

**Patients and diagnostics of HS:** Our study was approved by the responsible local ethics committee and performed with written informed consent from patients, parents and/or legal guardians as appropriate. The index group consisted of 35 consecutive children, adolescents and young adults who were diagnosed in our tertiary expertise centre for paediatric haematology in Austria between 2015 and 2020 with HS according to ICSH guidelines [King M-J, et al. Int J Lab Hematol. 2015; 37(3):304-325]. All index patients were referred to our centre because of hemolytic anemia. Laboratory and genetic HS diagnostics were subsequently attempted to be performed in all first-degree family members (a total of 113 first-grade members in 35 unrelated families were analyzed), and further identified 34 first-degree family members with HS (ICSH guidelines); hence the HS cohort encompassed 69 patients. Clinical (family history, ancestry, RBC transfusions, abdomen sonography, surgical reports) and laboratory data (erythropoietic and biochemical haemolytic parameters and eosin-5-maleimide (EMA) binding test or osmotic fragility test) were analyzed retrospectively. Blood smears from all 113 individuals were analyzed by one of the authors (L.K.), and a diagnosis of HS confirmed in all 69 patients. EMA binding analysis was performed as reported [King MJ et. al Br J Haematol. 2000 Dec;111(3):924-33.]. All serial blood analyses (i.e., ≥3 samples) from specimens taken before splenectomy were performed in our hospital laboratory and were available in the index group and in eight first-degree family members with HS (total cohort N=43 patients). Less than three blood samples taken before splenectomy were analyzed in our hospital laboratory in additional 13 family members with HS, and the cohort for genotype/phenotype corelation analyses encompassed 56 patients. Thirteen family members with HS had previous splenectomies and were not included into the genotype-phenotype analyses. Mild, moderate and severe forms of HS were classified based on Hb level, reticulocyte count, total bilirubin levels and number of RBC transfusions (modified from Eber S & Lux SE [Eber S & Lux SE. Semin Hematol. 2004 Apr;41(2):118-41] and van Vuren et al. 2019 [van Vuren A et. al Hemasphere. 2019 Aug 7;3(4):e276.]).

**Statistics**: Peripheral blood levels of the patients, or the respective medians in case of multiple measurements, were analyzed using ANOVA or KruskalWallis test depending on the presence of a normal distribution. The post hoc group comparisons were performed with Tukey's test or Dunn's test respectively and p-values were adjusted for multiple testing. Statistical analysis and figure creation was were performed using R statistics.

**Targeted NGS panel sequencing and whole exome sequencing (WES):** For targeted sequencing, we custom-designed a haematology-focused NGS panel as described previously [Kager L, et al. Br J Haematol. 2018 Jul;182(2):251-258]. For WES, the Illumina TrueSeq Rapid Exome kit and the Illumina HiSeq3000 system were used as described [Salzer E, et al. Nat Immunol. 2016 Dec;17(12):1352-1360; Ozen A, et al. N Engl J Med. 2017 Jul 6;377(1):52-61]. Briefly, reads were aligned to the human genome version 19 by means of the Burrows-Wheeler Aligner (BWA), and single nucleotide variants (SNVs) and insertions/deletions were called and annotated using the Genome Analysis Tool Kit (GATK, Broad Institute) and VEP, respectively. The obtained list was then filtered according to the presence of variants with a minor allele frequency (MAF) >0.01 in 1,000 Genomes, gnomAD, and dbSNP build 149. After further filtering steps for nonsense, frameshift, missense, and splice-site variants as well as small in-frame deletions and insertions using VCF.Filter software [Müller H et. al Nucleic Acids Res. 2017 Jul 3;45(W1):W567-W572.], an internal database was used to filter out recurrent variants. Moreover, variants were prioritized using tools such as SIFT, Polyphen-2 and the combined annotation dependent depletion (CADD) score [Kircher M et. al Nat Genet. 2014 Mar;46(3):310-5], to predict the deleteriousness of a given variant. PopViz, a webserver for visualizing minor allele frequencies (MAF) and damage prediction scores of human genetic variations (CADD) was used to analyze and depict all identified variants [Zhang P et. al Bioinformatics. 2018 Dec 15;34(24):4307-4309]. All clinically relevant HS-related genetic variants identified in the index group were confirmed by Sanger sequencing. In addition, Sanger sequencing for these variants was also performed in all available first grade family members. The *LELY* allele (or α^LELY^) [Wilmotte R, et al. J Clin Invest. 1993 May;91(5):2091-6] and the αLEPRA [H Wichterle H et al. J Clin Invest. 1996 Nov 15;98(10):2300-7] allele status was also analysed from the WES data in all index patients and selected family members.

**Identification of CNVs on NGS data:** CNVs were called from WES data for each sample as part of a cohort by applying CodeX [Jiang Y et. al Genome Biol. 2018 Nov 26;19(1):202.] and ExomeDepth [Plagnol V et. al Bioinformatics. 2012 Nov 1;28(21):2747-54.]. Cohorts were defined by grouping samples with similar read depth and captured regions to reduce technical artifacts. For each sample, the identified CNV calls were collected, deletions selected and identical calls from CodeX and ExomeDepth (same start/end position) combined. Each CNV region was annotated with supporting evidence from the CNV callers where available, Ensembl [Cunningham F et. al Nucleic Acids Res. 2022 Jan 7;50(D1):D988-D995.] gene annotation (GRCh37.87), haploinsufficiency score [Huang N et. al PLoS Genet. 2010 Oct 14;6(10):e1001154], pli [Lek M et. al Nature. 2016 Aug 18;536(7616):285-91] and the internal CNV frequency for each gene. The analysis was performed using an R script and the result stored as a tab delimited text file for further analysis [Lawrence M et. al Bioinformatics. 2009 Jul 15;25(14):1841-2 and Lawrence M et. al Stat Sci. 2014 May;29(2):214-226].

**Array CGH analysis:** The DNA sample was processed on the CytoScan™ HD Array (ThermoFisher Scientific/ Applied Biosystems™) and analyzed using the Chromosome Analysis Suite version 3.3 (ChAS; ThermoFisher Scientific/ Applied Biosystems™) software package. All aberrations were mapped to the Genome Reference Consortium GRCh37, UCSC genome assembly hg19 reference genome. Genomic segments were filtered according to the following parameters: losses ≥25 markers and ≥25kb size; gains ≥50 markers and ≥50 kb size; LOH regions ≥50 markers and ≥3 Mb size. Copy number variants (CNVs) arising from B-cell and T-cell antigen receptor gene rearrangements as well as all known common benign CNVs were excluded.

**RT-PCR analysis:** Total RNA was isolated from peripheral blood using the QIAamp® RNA Blood mini Kit (Qiagen GmbH, Hilden, Germany). RNA was reverse transcribed using random hexamers and 200 units of M-MLV reverse transcriptase (Promega, Mannheim Germany) at 42°C for 60 minutes. RT-PCR primers were designed in exons 26 and 34 of *ANK1* (NM_000037.3). Primers and details regarding RT-PCR reaction conditions are available on request. Sanger sequencing of RT-PCR products was done at Eurofins Genomics (Köln, Germany).

**Assessment of pathogenicity:** The pathogenicity of the identified variants was classified according to the American College of Medical Genetics and Genomics/Association for Molecular Pathology (ACMG/AMP) criteria [Richards S, et al. Genet Med. 2015 May;17(5):405-24]. Identified disease causing variants were compared with data from the literature [https://www.ncbi.nlm.nih.gov/pubmed/, search terms: identified variant(s) or ANK1, or SLC4A1, or SPTB, or SPTA1 and hereditary spherocytosis; last accessed March 2023] the Red Cell Membrane Disorder Database (https://research.nhgri.nih.gov/RBCmembrane; last accessed March 2023), and with ClinVar Miner (https://clinvarminer.genetics.utah.edu/; last accessed March 2023; variants were only considered as described, if basic clinical data were available) and the results for each patient are provided in the supplement.

In addition, we used the UniProt [(https://www.uniprot.org/)](https://www.uniprot.org/) and the Repository of Enhanced Structures of Proteins Involved in the Red blood cell Environment (RESPIRE) databases ([https://www.dsimb.inserm.fr/respire/)](https://www.dsimb.inserm.fr/respire/) as well as published data on the architecture of the human erythrocyte ankyrin-1 and spectrin complex [Xia X et. al Nat Struct Mol Biol. 2022 Jul;29(7):698-705; Vallese et al. Nat Struct Mol Biol. 2022 Jul;29(7):706-718; Ipsaro JJ et. al Blood. 2010 May 20;115(20):4093-101; Yasanuga et al. J Mol Biol. 2012 Apr 6;417(4):336-50 and Lux SE 4th. Blood. 2016 Jan 14;127(2):187-99] to assess the site of the identified amino acid changes within the encoded proteins.

**Supplemental Clinical and Laboratory Results**

**Table S1a.** Laboratory and clinical data before splenectomy in 16 index patients with pathogenic heterozygous variants in *ANK1*.

| **Patient ID (age/years^1^, sex, ancestry)** | **Hb level median, range g/dl** | **Reticulocytes median, range %** | **Total bilirubin median, range µmol/L** | **Eosin-5maleimide binding %** | **Grading**  **of disease severity** | | **Maximal**  **spleen size in cm** | **Gall stones (Age at diagnosis)** | **RBC**  **transfusions** | **Surgical therapy (age at procedure)** |
| --- | --- | --- | --- | --- | --- | --- | --- | --- | --- | --- |
| P1 (3, m, CE) | 10.6,  10.4 to 10.8 | 7.8,  7.3 to 8.3 | 13.6,  13.6 to 17 | 65 | Moderate | | 7 | No | 0 | None |
| P2 (18, m, CE) | 7.6,  5.1 to 9.7 | 14.7,  6.3 to 18.5 | 62.9  18.7 to 91.8 | 75^2^ | Severe | | 15 | Yes (4 years) | 17 | Cholecystectomy and subtotal splenectomy (5 years); splenectomy (16  years) |
| P3 (10, f, Palestine) | 9.4,  7.8 to 10.4 | 11.6,  6.6 to 19.6 | 59.5,  32.3 to 78.2 | 69 | Moderate | | 14 | No | 0 | None |
| P4 (4, f, CE) | 9.2,  6.9 to 14.9 | 6.4,  1.5 to 10.9 | 35.7,  22.1 to 309.4 | 67 | Moderate | | 7 | No | 5 | None |
| P5 (17, m, CE) | 9.5,  6.8 to 10.2 | 10.7,  0.2 to 17.2 | 32.3,  22.1 to 68 | 70 | Moderate | | 15 | Yes (4 years) | 3 | Cholecystectomy and splenectomy (14 years) |
| P6 (4, M, CE) | 9, 6.2 to 13 | 8.3,  1.3 to 13.9 | 17,  10.2 to 45.9 | 66 | Moderate | | 7.5 | No | 6 | None |
| P7 (6, f, CE) | 8.5,  6.7 to 11.4 | 10.4,  2.8 to 15.8 | 56.9,  17 to 91.8 | 60 | Severe | | 14 | Yes (5 years) | 17 | None |
| P8 (15, m, CE) | 8.1,  4.3 to 9.5 | 9.4,  0.1 to 15.3 | 44.2,  34 to 59.5 | 63 | Moderate | | 20 | No | 4 | Splenectomy (12 years) |
| P9 (13, m, CE) | 10.9,  6.5 to 12.1 | 9.4,  6.6 to 12.2 | 49.3,  34 to 76.5 | 72 | Moderate | | 16 | Yes (9 years) | 1 | None |
| P10 (3, f, CE) | 9.7,  7.5 to 14 | 6.2,  2.3 to 10.8 | 17,  11.9 to 265.2 | 63 | Moderate | | 7.6 | No | 4 | None |
| P11 (14, f, CE) | 11.1,  10.4 to 11.5 | 11.1,  10.2 to 12 | 59.5,  44.2 to 107.1 | 64 | Moderate | | 14 | No | 0 | None |
| P12 (6, f, CE) | 10.7,  6 to 13 | 5.6,  0.1 to 10.5 | 25.5,  17 to 52.7 | 66 | Mild | | 8 | No | 3 | None |
| P13 (20, m, Iran) | 12.7,  11.9 to 13.1 | 5.9,  4.9 to 8.1 | 68,  54.4 to 73.1 | 73 | Mild | | 15 | No | 0 | None |
| P14 (4, m, CE) | 9.7,  7.9 to 11.9 | 6.2,  0.5 to 12.8 | 25.5  3.4 to 42.5 | 63 | Moderate | | 8 | No | 5 | None |
| P15 (17, m, CE) | 13.4,  11.6 to 13.6 | 9.3,  6.1 to 11.7 | 129.2,  93.5 to 176.8 | 71 | Moderate | | 18 | Yes (11 years) | 1 | Cholecystectomy and splenectomy (16 years) |
| P16 (15, m, Chechen) | 12.3,  8.2 to 14.6 | 6.4,  2.4 to 10.1 | 35.7,  20.4 to 52.7 | 78 | | Moderate | 18.5 | Yes (4 years) | 0 | ERCP with removal of solitary gall stone (4 years) |

^1^Age at analysis, ^2^Assessed after subtotal splenectomy, initial diagnosis was established via pathologic osmotic resistance testing (Glycerol lysis time, result < 1 minute, normal > 30 minutes); Abbreviations: CE, Central European; ERCP, endoscopic retrograde cholangiopancreatography; f, female; m, male

**Table S1b.** Laboratory and clinical data in five index patients with pathogenic heterozygous or homozygous variants in *SLC4A1*.

| **Patient ID (age/years^^[[1]](#footnote-2)^^, sex, ancestry)** | **Hb level median, range g/dl** | **Reticulocytes median, range %** | **Total bilirubin median, range µmol/L** | **Eosin-5maleimide binding %** | **Grading**  **of disease severity** | **Maximal**  **spleen size in cm** | **Gall stones (Diagnosis)** | **RBC**  **transfusions** | **Surgical therapy (age at procedure)** |
| --- | --- | --- | --- | --- | --- | --- | --- | --- | --- |
| P17 (4, f, CE) | 11.3,  9 to 13.1 | 4.9,  2.1 to 7.5 | 31.5,  17 to 161.5 | 68 | Mild | 8.6 | No | 0 | None |
| P18 (19, m, CE) | 11,  4.1 to 13.9 | 7.6,  0.9 to 12.8 | 100.3,  25.5 to 413.1 | 62 | Moderate | 18 | Yes (4 years) | 2 | ERCP 2x, cholecystectomy (14 years), splenectomy (17 years) |
| P19^2^ (9, m, Turkish) | Regular transfusions | 4.6,  2.1 to 73 (regular transfusions) | 15.3,  8.5 to 30.6 (regular transfusions) | Normal during regular transfusions | Severe | 14 | No | Regular since birth, less often after  splenectomy | Splenectomy (6 years) |
| P20 (13, f,  CE) | 11.9,  11.2 to 14.2 | 4.7,  4.2 to 9.4 | 66.3,  44.2 to 124.1 | 65 | Mild | 10 | Yes (10 years) | 0 | None |
| P21 (6, f, CE) | 12.5,  8.3 to 13.3 | 4.6,  2.1 to 73 | 15.3,  8.5 to 30.6 | 75 | Mild | 8 | No | 0 | None |

**Table S1c.** Laboratory and clinical data before splenectomy in 11 index patients with pathogenic heterozygous variants in *SPTB*.

| **Patient ID (age/years^^[[2]](#footnote-3)^^, sex, ancestry)** | **Hb level median, range g/dl** | **Reticulocytes median, range %** | **Total bilirubin median, range µmol/L** | **Eosin-5maleimide binding %** | **Grading**  **of disease severity** | **Maximal**  **spleen size in cm** | **Gall stones (Age at diagnosis)** | **RBC**  **transfusions** | **Surgical therapy (age at procedure)** |
| --- | --- | --- | --- | --- | --- | --- | --- | --- | --- |
| P22 (13, f,  CE) | 12.1,  10.1 to 13.3 | 6,  2.7 to 7.9 | 35.7,  23.8 to 39.1 | 67 | Mild | 10.5 | No | 0 | None |
| P23 (7, f, CE) | 8.9,  7.8 to 10.4 | 9.9,  4.3 to 16.0 | 34,  17 to 85 | 69 | Moderate | 10 | Yes (4 years) | 0 | None |
| P24 (16, m, CE) | 10.4,  4.7 to 13 | 8.1,  0.5 to 14.4 | 92.7,  13.6 to 147.9 | 74 | Moderate | 17 | No | 1 | None |
| P25 (10, f,  CE) | 10.1,  4.8 to 11.8 | 9.7,  0.8 to 20.1 | 49.3,  27.2 to 457.3 | 59 | Moderate | 13.2 | Yes (6 years) | 1 | ERCP (6 years) |
| P26 (19, f,  CE) | 10.7,  5.4 to 11 | 7.5,  7 to 8.8 | 56.1  37.4 to 59.5 | 73 | Moderate | 19.5 | Yes (n.a.) | 3 | Cholecystectomy (17 years) |
| P27 (11, f,  CE) | 9.7,  6.6 to 11 | 6.7,  2.4 to 12.9 | 61.2,  35.7 to 197.2 | 71 | Moderate | 14.4 | Yes (6 years) | 4 | Cholecystectomy and subtotal splenectomy (7 years) |
| P28^2^ (2, f, CE) | 9.4,  6.3 to 12.8 | 9.7,  5.3 to 11.7 | 91.8,  52.7 to 178.5 | 68 | Moderate | 6 | No | 3 | None |
| P29 (10, f,  CE) | 11.7,  8.2 to 13.3 | 3.0,  0.7 to 8.6 | 25.5,  11.9 to 54.4 | 77 | Mild | 9.7 | No | 0 | None |
| P30 (13, m, CE) | 11.1,  10.1 to 13 | 6.0,  2.9 to 9.3 | 28.9,  10.2 to 59.5 | 65 | Mild | 13.3 | No | 0 | None |
| P31 (18, m, CE) | 8.7, 3.4 to 12.2 | 11.9,  0.2 to 15.8 | 147.9,  10.2 to 537.2 | 62 | Severe | 24.5 | Yes (10 years) | 3 | Cholecystectomy and splenectomy (16 years) |
| P32 (23, f,  CE) | 11.2,  7.3 to 13.2 | 7.6,  1.2 to 15.1 | 68,  25.5 to 23.9 | 74 | Moderate | 21 | Yes (11 years) | 4 | Cholecystectomy (13 years) |

**Table S1d.** Laboratory and clinical data before splenectomy in 2 index patients with pathogenic compound heterozygous variants in *SPTA1*.

| **Patient ID (age/years^^[[3]](#footnote-4)^^, sex, ancestry)** | **Hb level median, range g/dl** | **Reticulocytes median, range %** | **Total bilirubin median, range µmol/L** | **Eosin-5maleimide binding %** | **Grading**  **of disease severity** | **Maximal**  **spleen size in cm** | **Gall stones (Diagnosis)** | **RBC**  **transfusions** | **Surgical therapy (age at procedure)** |
| --- | --- | --- | --- | --- | --- | --- | --- | --- | --- |
| P33 (17, f,  CE) | 8.1,  4 to 11 | 8.7,  0.2 to 15.8 | 73.1,  23.8 to 334.9 | 66 | Moderate | 13 | Yes (5 years) | 6 | Cholecystectomy and splenectomy (8 years) |
| P34 (11, m, CE) | 10,  5.8 to 11.8 | 6.9,  0.2 to 12.5 | 45.9,  11.9 to 95.2 | 63 | Moderate | 16.4 | Yes (7 years) | 1 | cholecystectomy and subtotal splenectomy (8 years) |

**Table S1e.** Laboratory and clinical data in one pediatric index patient with no unequivocal causative candidate variants identified yet.

| **Patient ID (age/years^^[[4]](#footnote-5)^^, sex, ancestry)** | **Hb level median, range g/dl** | **Reticulocytes median, range %** | **Total bilirubin median, range µmol/L** | **Eosin-5maleimide binding %** | **Grading**  **of disease severity** | **Maximal**  **spleen size in cm** | **Gall stones (Diagnosis)** | **RBC**  **transfusions** | **Surgical therapy (age at procedure)** |
| --- | --- | --- | --- | --- | --- | --- | --- | --- | --- |
| P35 (18, m, CE) | 11,  4.1 to 13.9 | 7.6,  0.9 to 12.8 | 100.3,  25.5 to 413.1 | 62 | Moderate | 18 | Yes (4 years) | 2 | ERCP 2x, cholecystectomy (14 years), splenectomy (17 years) |

**Table S2a.** Laboratory (Reference laboratory St. Anna Children’s Hospital) and clinical data before splenectomy in 16 first-grade relatives with HS phenotypes and pathogenic heterozygous variants in *ANK1*.

| **Patient ID (age^1^, sex, ancestry, relatives)** | **Hb level median, range g/dl** | **Reticulocyte s median, range %** | **Total bilirubin median, range µmol/L** | **Eosin-5maleimide binding %** | **Grading**  **of disease severity** | | **Maximal**  **spleen size in cm** | **Gall stones (Age at diagnosis)** | **RBC**  **transfusions** | **Surgical therapy (age at procedure)** |
| --- | --- | --- | --- | --- | --- | --- | --- | --- | --- | --- |
| P36 (42, f, CE, mother of P1) | 11.1 | 11.4 | 28.9 | 63 | Moderate | | n.a | Yes (n.a) | 3 | Cholecystectomy (35 years) |
| P37 (46, m, CE, father of P2) | n.a. | n.a. | n.a. | 71^2^ | n.a. | | n.a. | Yes (n.a.) | Yes (n.a.) | Cholecystectomy and splenectomy (7 years) |
| P38 (33, f, Palestine, mother of P3) | n.a. | n.a. | n.a. | 72^2^ | n.a. | | n.a. | No | 0 | Splenectomy (10 years) |
| P39 (15, f, Palestine, sister of P3) | n.a. | n.a. | n.a. | 71^2^ | n.a. | | n.a. | No | Yes (10) | Splenectomy (8 years) |
| P40 (14, f, Palestine, sister of P3) | n.a. | n.a. | n.a. | 73^2^ | n.a. | | n.a. | No | 0 | Splenectomy (7 years) |
| P41 (37, m, CE, father of P4) | n.a. | n.a. | n.a. | 73^2^ | n.a. | | n.a. | No | Yes (n.a.) | Splenectomy (16 years) |
| P42 (7, f, CE, sister of P4) | 9.2,  6.5 to 12.4 | 7.4,  0.08 to 12.3 | 28.9,  18.7 to 93.5 | 62 | Moderate | | 10.9 | No | 1 | None |
| P43 (33, f, CE, mother of P6) | 12.8 | 13 | 62.9 | 70 | Moderate | | n.a. | Yes (13 years) | 0 | Cholecystectomy (25 years) |
| P44 (39, m, CE, father of P7) | n.a | n.a | n.a | n.a | n.a. | | n.a | Yes (7 years) | Yes (2) | Splenectomy (12 years) |
| P45 (8, f, CE, sister of P7) | 10.3  9.5 to 10.6 | 8.4  3.2 to 8.7 | 25.7  10.3 to 27.4 | 70 | Moderate | | 12 | Yes (7 years) | 0 | none |
| P46 (44, m, CE, father of P8) | n.a | n.a | n.a | 71^2^ | n.a. | | n.a | No | Yes (>10) | Splenectomy (12 years) |
| P47 (48, m, CE, father of P9) | n.a | n.a | n.a | 76^2^ | n.a. | | n.a | n.a. | Yes (n.a.) | Splenectomy (16 years) |
| P48 (36, f, CE, mother of P10) | n.a | n.a | n.a | 73^2^ | n.a. | | n.a. | Yes (n.a.) | 0 | Cholecystectomy and splenectomy (17 years) |
| P49 (37, f, CE, mother of P12) | n.a | n.a | n.a | 77^2^ | n.a. | | n.a. | Yes (n.a.) | Yes (n.a.) | Cholecystectomy and  splenectomy (14 years) |
| P50 (14, m, CE, brother of P12) | 12.6,  10.9 to 14.2 | 10.3,  8.2 to 10.7 | 69.7,  54.4 to 404.6 | 58 | | Moderate | 14.5 | Yes (11 years) | 0 | Cholecystectomy and  splenectomy (12 years) |
| P51 (37, m, CE, father of P14) | n.a | n.a | n.a | 71^2^ | | n.a. | n.a. | Yes (n.a.) | Yes (n.a.) | Cholecystectomy (32 years) and splenectomy (22 years) |

^1^Age in years at analysis. ^2^Testing was performed in our hospital reference laboratory after splenectomy. Abbreviations: CE, Central European; f, female; m, male

**Table S2b.** Laboratory (Reference laboratory St. Anna Children’s Hospital) and clinical data before splenectomy in 10 first-grade relatives with HS phenotypes and pathogenic heterozygous variants in *SLC4A1*.

| **Patient ID (age^1^/years, sex, ancestry, relatives)** | **Hb level median, range g/dl** | **Reticulocytes median, range %** | **Total bilirubin median, range µmol/L** | **Eosin-5maleimide binding %** | **Grading**  **of disease severity** | | **Maximal**  **spleen size in cm** | **Gall stones (Age at diagnosis)** | **RBC**  **transfusions**  **(Number)** | | **Surgical therapy (age at procedure)** |
| --- | --- | --- | --- | --- | --- | --- | --- | --- | --- | --- | --- |
| P52 (38, f, CE, mother of P17) | 13.1 | 4.9 | 32.5 | 70 | Mild | | n.a | n.a | 0 | | None |
| P53 (46, f, CE, mother of P18) | 13.6 | 5.0 | 29.1 | 65 | Mild | | n.a. | None | 0 | | None |
| P54 (43, f, Turkish, mother of patient P19) | 11.5 | 2.7 | 20.5 | 87 | Mild | | n.a. | Yes (n.a.) | 0 | | Cholecystectomy (n.a.) |
| P55 (46, m, Turkish, father of P19) | 12.7 | 2.0 | 15.3 | 83 | Mild | | n.a. | None | 0 | | None |
| P56 (23, m, Turkish, brother of P19) | 13.7  12.3 to 14.8 | 2.2  1.7 to 3.9 | 25.7  24.0 to 30.8 | 76 | Mild | | 13,5 | None | Yes (1  infant) | | None |
| P57 (17, f, Turkish, sister of P19) | 12.5  10.5 to 14.2 | 2.5  1.6 to 2.6 | 8.6  5.1 to 18.8 | 73 | Mild | | n.a | None | 0 | | None |
| P58 (55, m,  CE, father of P20) | 14.4 | 5.7 | 57.8 | 65 | Moderate | | 17 | Yes (<20 years) | Yes (2) | | Cholecystectomy (45 years) |
| P59 (39, f, CE, mother of P21) | 12.9 | 8.0 | 35.9 | 82 | Moderate | | 16 | None | Yes (2, after delivery) | | None |
| P60 (10, m,  CE, brother of P21) | 13.5,  12.6 to 14.1 | 3.8,  1.3 to 52.0 | 22.1,  13.6 to 32.3 | 72 | | Mild | 10.8 | No | 0 |  | None |

^1^Age at analysis. Abbreviations: CE, Central European; f, female; m, male

**Table S2c.** Laboratory (Reference laboratory St. Anna Children’s Hospital) and clinical data before splenectomy in six first-grade relatives with HS phenotypes and pathogenic heterozygous variants in *SPTB*.

| **Patient ID (age^^[[5]](#footnote-6)^^/years, sex, ancestry, relatives)** | **Hb level median, range g/dl** | **Reticulocytes median, range %** | **Total bilirubin median, range µmol/L** | **Eosin-5maleimide binding %** | **Grading**  **of disease severity** | **Maximal**  **spleen size in cm** | **Gall stones (Age at diagnosis)** | **RBC**  **transfusions** | **Surgical therapy (age at procedure)** |
| --- | --- | --- | --- | --- | --- | --- | --- | --- | --- |
| P61 (51, m,  CE, father of P22) | 14.5 | 10.6 | 74.8 | 82 | Moderate | n.a. | Yes (n.a.) | 0 | No |
| P62 (43, f, CE, mother of P23) | n.a. | n.a. | n.a. | 74^2^ | n.a. | n.a. | Yes (n.a.) | Yes (n.a.) | Splenectomy (11 years) |
| P63 (44, m,  CE, father of P27) | 11.9 | 13.0 | 63.3 | 72 | Moderate | n.a. | None | 0 | None |
| P64 (30, m,  CE, father of P28) | 9.0,  8.4 to 14.6 | 11.8,  9.2 to 14 | 119,  76.5 to 137.7 | 72 | Severe | 16 | Yes (15 years) | 12 | Cholecystectomy and splenectomy (18 years) |
| P65 (43, m,  CE, father of P29) | 14.3 | 4.5 | 29.1 | 82 | Mild | n.a. | None | 0 | None |
| P66 (14, m, brother of P29) | 12.9 | 1.9 | 8.5 | 80 | Mild | normal | None | 0 | None |
| P67 (45, m,  CE, father of P30) | 16.9 | 9.4 | 133.4 | 64 | Moderate | n.a. | Yes (n.a.) | 1 | Cholecystectomy (24 years) |
| P68 (57, m,  CE, father of P32) | n.a | n.a. | n.a. | 74^1^ | n.a. | n.a. | Yes (n.a.) | n.a. | Cholecystectomy and splenectomy (8 years) |

**Table S2d.** Laboratory (Reference laboratory St. Anna Children’s Hospital) and clinical data before splenectomy in one first-grade relative with HS phenotype and pathogenic compound heterozygous variants in *SPTA1*

| **Patient ID (age^^[[6]](#footnote-7)^^/years, sex, ancestry)** | **Hb level median, range g/dl** | **Reticulocytes median, range %** | **Total bilirubin median, range µmol/L** | **Eosin-5maleimide binding %** | **Grading**  **of disease severity** | **Maximal**  **spleen size in cm** | **Gall stones (Age at diagnosis)** | **RBC**  **transfusions** | **Surgical therapy (age at procedure)** |
| --- | --- | --- | --- | --- | --- | --- | --- | --- | --- |
| P69 (13, f, CE, sister of P33) | 9.2,  5.1 to 12.4 | 6,  2.7 to 7.9 | 30.6,  15.3 to 108.8 | 64 | Moderate | 16.5 | Yes (11 years) | 4 | None |

**Table S3.** Demographics and clinical characteristics of genotype groups

|  | ANK1  n = 21 | SLC4A1  n = 14 | SPTB  n = 17 | SPTA1  n = 3 | unknown = 1 |
| --- | --- | --- | --- | --- | --- |
| Sex, m/f | 11 / 10 | 6 / 8 | 9 / 8 | 1 / 2 | 1 / 0 |
| Age, years | 13.0 ± 9.9 | 26.3 ± 17.5 | 21.7 ± 15.2 | 13.7 ± 3.1 | 18 |
| Hemoglobin, g/dl | 10.4 ± 1.7 | 12.7 ± 1.0^a^ | 11.4 ± 2.2 | 9.1 ± 1.0 | 11.0 |
| Reticulocytes, % | 9.0 ± 2.5 | 4.5 ± 1.9^a^ | 8.1 ± 3.1 | 7.2 ± 1.4 | 7.6 |
| Total bilirubin, µmol/l | 45.1 ± 26.4 | 35.5 ± 25.4^a^ | 65.8 ± 40.0 | 49.9 ± 21.5 | 100.3 |
| EMA binding, % | 67.0 ± 5.1 | 72.5 ± 7.8 | 71.2 ± 6.7 | 64.3 ± 1.5 | 62 |
| Gall stones | 10 (48%) | 4 (29%) | 9 (53%) | 3 (100%) | 1 (100%) |
| Any RBC transfusions | 13 (62%) | 4 (31%) | 9 (53%) | 3 (100%) | 1 (100%) |
| Splenectomy | 5 (24%) | 2 (14%) | 3 (18%) | 2 (67%) | 1 (100%) |
| Cholecystectomy | 6 (29%) | 3 (21%) | 6 (35%) | 2 (67%) | 1 (100%) |
| Ancestry, Central European /  non-Central European | 18 / 3 | 9 / 5 | 17 / 0 | 3 / 0 | 1 / 0 |

^a^ One patient with severe transfusion dependence was removed for hemoglobin, reticulocyte and total bilirubin analysis in SLC4A1 group.


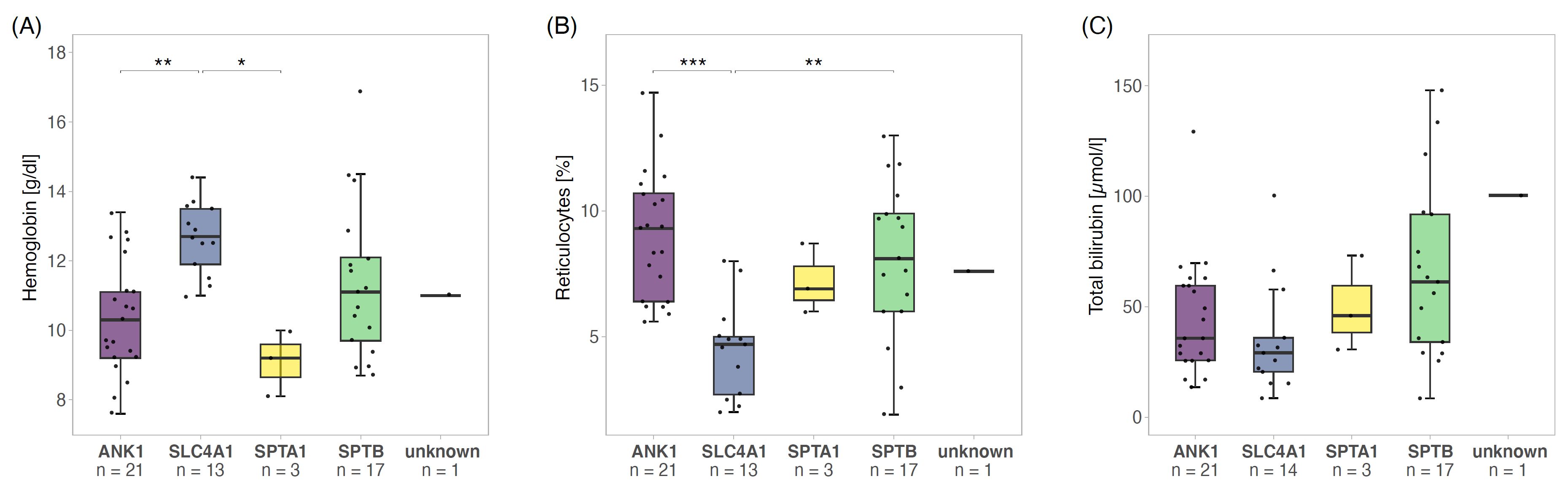


**Figure S1**. Phenotype differences between genotype groups. Boxplots are shown for (A) haemoglobin, (B) reticulocytes and (C) bilirubin from 35 patients in the core group and 21 family members for *ANK1*, *SLC4A1*, *SPTA1*, *SPTB* and unknown genotype, respectively. One subject in the *SLC4A1* group with severe transfusion dependence was removed for haemoglobin analysis; *: adjusted p value < 0.05; **: adj-p < 0.01; ***: adj-p < 0.001

# Supplemental Genetic Results

We established a comprehensive NGS-based panel covering 292 candidate genes for different types of inborn hematologic diseases as previously described [Kager et al. Br J Haematol 2018;182:251-258]. Herein, we filtered for genes which are known to cause HS, that is *ANK1* (encoding ankyrin1; associated disease according to the OMIM classification: #182900, HS type 1), *SPTB* (encoding β-spectrin; OMIM #616649, HS type 2), *SPTA1* (encoding α-spectrin, OMIM #270970, HS type 3), *SLC4A1* (encoding solute carrier family 4 member 1 or band-3 or anionic exchanger-1 (AE1), OMIM #612653, HS type 4), and *EPB42* (encoding protein 4.2, OMIM #612690, HS type 5) [https://www.omim.org, accessed 3/2023] and allele frequency (AF) < 0.01. In patients in whom no potentially causative variants could be identified, WES analysis was performed. A table was created for each of the 35 index patients, which contain details on the most likely disease-causing variant(s), as per the following format:

| **GENE^1^** | **CHR^2^** | **POS^3^** | **IDS**^4^ | **REF**^5^ | **ALT**^6^ | **gnomAD_AF**^7^ | **gnomAD_AC**^8^ | **gnomAD_hom^9^** | **Consequence^10^** | **HGVSc (cDNA)^11^** | **HGVSp**  **(protein)^12^** |
| --- | --- | --- | --- | --- | --- | --- | --- | --- | --- | --- | --- |

| **READS^13^** | **SIFT^14^** | **PolyPhen^15^** | **CADD^16^** | **Method^17^** | **LELY^18^** | **αLEPRA^19^** | **PMID^20^** | **Pathogenicity**  **(split)^21^** |
| --- | --- | --- | --- | --- | --- | --- | --- | --- |

^1^the identified gene(s) symbol (GENE) according to the HUGO Gene Nomenclature Committee ([https://www.genenames.org/)](https://www.genenames.org/),

^2^the chromosome (CHR) on which the gene is localized ([https://www.ncbi.nlm.nih.gov/gene/)](https://www.ncbi.nlm.nih.gov/gene/),

^3^the position (POS) of the identified variant(s) in the GRCh37/hg19 assembly ([https://www.ncbi.nlm.nih.gov/assembly/GCF_000001405.13/)](https://www.ncbi.nlm.nih.gov/assembly/GCF_000001405.13/),

^4^the primary identifier for variants (SNP_ID) in the Single Nucleotide Polymorphism Database (dbSNP) of Nucleotide Sequence Variation

(https://www.ncbi.nlm.nih.gov/books/NBK21088/),

^5^the nucleotide present in the reference (REF) genome GRCh37/hg19,

^6^the nucleotide present in the patient alternatively (ALT) to the reference,

^7^the minor allele frequency (GnomAD_AF) provided for each specific variant in all populations/ethnicities by the Genome Aggregation Database

(https://gnomad.broadinstitute.org/),

^8^the allele counts (GnomAD_AC) provided by GnomAD,

^9^the homozygous counts out of the total allele counts (GnomAD_hom_counts) provided by GnomAD,

^10^the consequence type of each variant defined by the sequence ontology ([http://www.sequenceontology.org/)](http://www.sequenceontology.org/), which includes from synonymous, intronic, missense and 5’ or 3’ prime UTR, to stop gain, start lost, frameshift and splice region variants, among others,

^11^the Human Genome Variation Society coding sequence name (HGVSc (cDNA)) ([https://www.hgvs.org/)](https://www.hgvs.org/),

^12^the Human Genome Variation Society protein sequence name (HGVSc (protein)),

^13^the number of reads (READS) covering the specific position, and the results from in silico analyses using the prediction tools:

^14^Prediction program: SIFT ([http://sift.jcvi.org/)](http://sift.jcvi.org/) ( <https://pubmed.ncbi.nlm.nih.gov/12824425/>),

^15^Prediction program: PolyPhen ([http://genetics.bwh.harvard.edu/pph2/)](http://genetics.bwh.harvard.edu/pph2/) (<https://pubmed.ncbi.nlm.nih.gov/20354512/>),

^16^Prediction program: CADD v1.3 [(http://cadd.gs.washington.edu/)](http://cadd.gs.washington.edu/) (https://pubmed.ncbi.nlm.nih.gov/24487276/),

^17^the (Method) used to identify the variant: either whole exome sequencing, targeted sequencing or Sanger sequencing,

^18^the homozygous, heterozygous or wild type allele of LELY [(https://pubmed.ncbi.nlm.nih.gov/8486776/)](https://pubmed.ncbi.nlm.nih.gov/8486776/),

^19^the homozygous, heterozygous or wild type allele of αLEPRA (<https://www.ncbi.nlm.nih.gov/pmc/articles/PMC507680/>)

^20^the PubMed reference number of the corresponding publication, if a variant has been previously published in the literature,

^21^and the detailed annotation of pathogenicity based on the Standard Guidelines for the Interpretation of Sequence Variants [(https://pubmed.ncbi.nlm.nih.gov/25741868/](https://pubmed.ncbi.nlm.nih.gov/25741868/) and [https://pubmed.ncbi.nlm.nih.gov/31690835/)](https://pubmed.ncbi.nlm.nih.gov/31690835/).

The identified disease-causing variants were confirmed via Sanger sequencing, and Sanger sequencing of these variants was also performed in first grade family members. We also investigated the low-expression polymorphic allele α-LELY in *SPTA1* in all index patients and some family members. The α-LELY consists of a C>T substitution in position −12 of intron 45 (dimerization domain) causing exon 46 (18 bp) to be skipped in 50% of transcripts and the synthesis of α chains with the missing of 6 corresponding amino acids necessary to form dimers [Wilmotte R et al, 1997 Blood; 90(10):4188–4196]. Besides the α-LELY, we also investigated the αLEPRA allele, which contains a transition of C>T in position -99 of intron 30. This mutation enhances an alternative acceptor splice site 70 nucleotides upstream from the regular site. The alternative splicing causes a frameshift and premature termination of translation leading to a significant decrease in alpha spectrin production [H Wichterle H et al. J Clin Invest. 1996 Nov 15;98(10):2300-7].

Identified variants were classified according to the Standard Guidelines for the Interpretation of Sequence Variants [(https://pubmed.ncbi.nlm.nih.gov/25741868/](https://pubmed.ncbi.nlm.nih.gov/25741868/) and [https://pubmed.ncbi.nlm.nih.gov/31690835/)](https://pubmed.ncbi.nlm.nih.gov/31690835/). Identified disease-causing variants were compared with data from the literature ([https://www.ncbi.nlm.nih.gov/pubmed/,](https://www.ncbi.nlm.nih.gov/pubmed/) search terms: identified variant(s), or *ANK1*, or *SLC4A1*, or *SPTB*, or *SPTA1* and hereditary spherocytosis; accessed January 2023) the Red Cell Membrane Disorder Database ([https://research.nhgri.nih.gov/RBCmembrane;](https://research.nhgri.nih.gov/RBCmembrane) accessed January 2023), and with ClinVar Miner ([https://clinvarminer.genetics.utah.edu/;](https://clinvarminer.genetics.utah.edu/) accessed January 2023; variants were only considered as described, if basic clinical data were available) and the results for each patient are provided below.

We were able to identify the causative variant(s) in 34/35 index patients. In four of these 34 patients (P18, P24, P28 and P33) more than one variant remained after filtering. In one index patient (P35) we were not able to identify the molecular cause of disease unequivocally.

Recent publications provide novel and exciting insights into the architecture of the human erythrocyte ankyrin-1 complex [Xia et al. Nat Struct Mol Biol. 2022 Jul;29(7):698-705, Vallese et al. Nat Struct Mol Biol. 2022 Jul;29(7):706-718], and we herein discuss the identified variants also in the context of these data that were published in 2022.

Clinical, laboratory and treatment data are summarized in Tables S1a-e (index patients P1 to P35) and Tables S2a-d (affected family members P36 to P69).

Details on each of the 35 families are provided below. We grouped them according to the identified causative variants (*ANK1*, *SLC4A1*, *SPTB*, *SPTA1* and unknown). **Index patients (P) and their families (F) with causative variants in *ANK1***

**Family F1 (P1 and P36): Index group P1**, male, 3-years-old, Central Europe, moderate HS phenotype

| **GENE** | **CHR** | **POS** | **IDS** | **REF** | **ALT** | **gnomAD _AF** | **gnomAD _AC** | **gnomAD _hom** | **Consequence** | **HGVSc (cDNA)** | **HGVSp (protein)** | **READS** | **SIFT** | **PolyPhen** | **CADD** |
| --- | --- | --- | --- | --- | --- | --- | --- | --- | --- | --- | --- | --- | --- | --- | --- |
| *ANK1* | 8 | 41615606 | . | TC | T | NA | NA | NA | Frameshift | ENST00000289734.7:  c.76del | ENSP00000289734.7:  p.Asp26ThrfsTer11 | 216 | NA | NA | 35 |

| **Method** | **LELY** | **αLEPRA** | **PMID** | **Pathogenicity (split)** |
| --- | --- | --- | --- | --- |
| WES | Wild type | Wild type | - | PVS1+PM2+PM4+PP1+PP3+PP4 |

Abbreviations. NA, not annotated; ANK1, ankyrin 1, WES, whole exome sequencing.

Interpretation: The heterozygous *ANK1* NM_000037.3:c.76del (p.Asp26ThrfsTer11) variant was discovered in P1 via WES. He has a moderate HS phenotype (Table S1a). His mother P36, who underwent cholecystectomy for gall stones at age 35 years, also has a moderate HS phenotype (Table S2a) and carries the same heterozygote *ANK1* variant, as confirmed via Sanger sequencing. The healthy father carries wild type (wt) *ANK1* alleles. The *LELY* in the patient was wt.

The *ANK1* variant causes a frameshift resulting in an early premature stop codon, leading to an erroneous truncated protein or nonsense mediated mRNA decay. Moreover, there is a high CADD prediction score (i.e., 35) for the identified variant. The variant per se affects a D (aspartic acid, the first aa of an alpha helix, [https://www.dsimb.inserm.fr/respire/proteins/533)](https://www.dsimb.inserm.fr/respire/proteins/533), in AR1 of the 24 ankyrin repeats (ARs) at the N-terminal domain (Figure 1A). According to the publication of Vallese et al., this variant is localized in AR1 (AR1 encompass aa 11-43); the stop codon also affects AR1 [Vallese et al. Nat Struct Mol Biol. 2022 Jul;29(7):706-718]. Ankyrin-1 binds to the N and C domains of the membrane Rhesus protein RhCE via ankyrin repeats 1-5 [Vallese et al. Nat Struct Mol Biol. 2022 Jul;29(7):706-718].

*ANK1* c.76del is pathogenic (class 5) and the autosomal dominant inherited Mendelian genotype that causes the phenotype moderate HS in P1 and P36. This variant is novel.

**Family F2 (patientsP2 and P37): Index group patient P2**, male, 18-years-old, Central Europe, severe HS phenotype

| **GENE** | **CHR** | **POS** | **IDS** | **REF** | **ALT** | **gnomAD _AF** | **gnomAD _AC** | **gnomAD _hom** | **Consequence** | **HGVSc (cDNA)** | **HGVSp (protein)** | **READS** | **SIFT** | **PolyPhen** | **CADD** |
| --- | --- | --- | --- | --- | --- | --- | --- | --- | --- | --- | --- | --- | --- | --- | --- |
| *ANK1* | 8 | 41584836 | . | G | A | NA | NA | NA | Stop gain | ENST00000289734.7:  c.358C>T | ENSP00000289734.7:  p.Gln120Ter | 248 | NA | NA | 38 |

| **Method** | **LELY** | **αLEPRA** | **PMID** | **Pathogenicity (split)** |
| --- | --- | --- | --- | --- |
| Panel | Wild type | Wild type | 29797310  32266426  36598564 | PVS1+PM2+PM4+PP1+PP3+PP4 |

Interpretation: The heterozygous *ANK1* NM_000037.3:c.358C>T (p.Gln120Ter) nonsense variant was discovered in the P2 via our Hematology-Panel and first published by us in 2018 [Kager et al. Br J Haematol 2018;182:251-258]. He had a severe HS phenotype before splenectomy (subtotal with cholecystectomy at age 5 years, total splenectomy after spleen regrowth at age 16 years; Table S1a). His father (P37), who had a similar HS phenotype (spleen- and cholecystectomy at age 7 years; Table S2a) carries the same variant. The healthy mother carries wt *ANK1* alleles. The *LELY* in the patient was wt.

The stop-gain variant results in premature cessation of translation of messenger RNA into protein, resulting in an erroneous truncated protein or nonsense mediated mRNA decay. Moreover, there is a very high CADD prediction score (i.e., 38) for the identified variant. The variant per se affects a conserved Q (glutamine; first aa of a coil segment) ([https://www.dsimb.inserm.fr/respire/proteins/533)](https://www.dsimb.inserm.fr/respire/proteins/533) in ankyrin repeat 4 (AR4; aa 110-142) in the ANK1 membrane binding domain (Figure 1A) [Vallese et al. Nat Struct Mol Biol. 2022 Jul;29(7):706-718]. Ankyrin-1 binds to the N and C domains of the membrane Rhesus protein RhCE via ankyrin repeats 1-5, and this variant affects AR4 in this domain [Vallese et al. Nat Struct Mol Biol. 2022 Jul;29(7):706-718].

*ANK1* c.358C>T is pathogenic (class 5) and the autosomal dominant inherited Mendelian genotype that causes the severe HS phenotype in P2 and his father P37. This variant was subsequently described as a *de novo* heterozygous pathogenic variant in a 10-years-old Brazil patient with moderate HS phenotype [Svidnicki et al. Annals Hematol 2020; 99:955-962]. Whereas our patient carried *LELY* WT alleles, the patient from Brazil carried variant *LELY* (not otherwise specified). The ancestry of the Brazilian patient was not provided. In addition, the variant was recently reported in a 7-years-old male from India with moderate HS phenotype [More TA et al. Mol Genet Genomics. 2023 Jan 4. doi: 10.1007/s00438-022-01984-1].

## Family F3 (patients P3, P38, P39, and P40): Index group patient P3, female, 10-years-old, Palestine, moderate HS phenotype

| **GENE** | **CHR** | **POS** | **IDS** | **REF** | **ALT** | **gnomAD_AF** | **gnomAD _AC** | **gnomAD _hom** | **Consequence** | **HGVSc (cDNA)** | **HGVSp (protein)** | **READS** | **SIFT** | **PolyPhen** | **CADD** |
| --- | --- | --- | --- | --- | --- | --- | --- | --- | --- | --- | --- | --- | --- | --- | --- |
| *ANK1* | 8 | 41575215 | . | GC | G | NA | NA | NA | Frameshift | ENST00000289734.7:  c.1211del | ENSP00000289734.7:  p.Gly404AlafsTer2 | 528 | NA | NA | 35 |

| **Method** | **LELY** | **αLEPRA** | **PMID** | **Pathogenicity (split)** |
| --- | --- | --- | --- | --- |
| Panel | Heterozygous | Wild type | - | PVS1+PM2+PP1+PP3+PP4 |

Interpretation: The heterozygous *ANK1* NM_000037.3:c.1211delG (p.Gly404AlafsTer2) variant was discovered in the patient (P3) via Panel investigation. Her mother (P38) and affected sisters (P39 and P40), who have similar HS phenotypes (Tables S1a and S2a), carry the same variant confirmed via Sanger sequencing. The healthy father carries wt *ANK1* alleles. The patient carries a heterozygous *LELY* variant. Except for the index patient P3, all three affected family members (P38, P39, and P40) underwent splenectomies in Israel before presenting at our department.

The *ANK1* variant causes a frameshift resulting in a stop codon at aa 405, leading to an erroneous truncated protein or nonsense mediated mRNA decay. Moreover, there is a high CADD prediction score (i.e., 35) for the identified variant. It affects a highly conserved G (glycine, within a coil sequence)

[[https://www.dsimb.inserm.fr/respire/proteins/533]](https://www.dsimb.inserm.fr/respire/proteins/533) in AR13 (aa 403-435) of the ANK1 membrane binding domain (Figure 1A) [Vallese et al. Nat Struct Mol Biol. 2022 Jul;29(7):706-718]. Ankyrin1 binds to protein 4.2, which stabilizes the cytoplasmic domain of band 3 dimer, via ARs 6-13 [Xia et al. Nat Struct Mol Biol. 2022 Jul;29(7):698-705], and the variant affects this domain.

*ANK1* c.1211delG is pathogenic (class 5) and the autosomal dominant inherited Mendelian genotype that causes the phenotype moderate HS in P3, P38, P39 and P40. This variant is novel.

**Family F4 (patients P4, P41, P42): Index group patient P4**, female, 4-years-old, Central Europe, moderate HS phenotype

| **GENE** | **CHR** | **POS** | **IDS** | **REF** | **ALT** | **gnomAD _AF** | **gnomAD _AC** | **gnomAD _hom** | **Consequence** | **HGVSc (cDNA)** | **HGVSp (protein)** | **READS** | **SIFT** | **PolyPhen** | **CADD** |
| --- | --- | --- | --- | --- | --- | --- | --- | --- | --- | --- | --- | --- | --- | --- | --- |
| *ANK1* | 8 | 41573376 | . | C | T | NA | NA | NA | Intron | ENST00000289734.7:  c.1405-9G>A | ENSP00000289734.7:  p.Asp469GlyfsTer21 | 121 | NA | NA | 2.1 |

| **Method** | **LELY** | **αLEPRA** | **PMID** | **Pathogenicity (split)** |
| --- | --- | --- | --- | --- |
| Panel + WES | Wild type | Wild type | 32436265, 33868383  36071563, 36203343 36928866 | PVS1+PM2+PM4+PM6+PP4 |

Interpretation: The heterozygous intronic *ANK1* NM_000037.3:c.1405-9G>A (p.Asp469GlyfsTer21) variant was discovered in the patient (P4) via Panel and WES investigation. Her father (P41) and affected sister (P42), who also have a similar HS phenotype (Tables S1a and S2a) all carry the same variant in intron 13, confirmed via Sanger sequencing. The healthy mother carries wt *ANK1* alleles. The patient is *LELY* wt. The father underwent

‘curative’ splenectomy at age 16 years. This intronic *ANK1* variant causes the generation of a new splice-acceptor site, causing the incorporation of seven nucleotides to the canonical coding sequence, creating a frameshift and premature stop, leading to an erroneous truncated protein or nonsense mediated mRNA decay (Figure S2). There is a very low CADD prediction score (i.e., 2.069) due to its annotation as a low impact variant. The pathogenic role of this intronic variant was recently established via minigene assays [Lunati-Rozie A, et al. Br J Haematol. 2023 Mar 16. doi: 10.1111/bjh.18760. Online ahead of print]. This variant was also identified in patient P5 (*de novo*), whose family is not related to family F4.

The intronic *ANK1* c.1405-9G>A variant is pathogenic (class 5) and the autosomal dominant inherited Mendelian genotype that causes the moderate HS phenotype in P4, P41 and P42; and in the unrelated P5 (see below). This variant has recently been described in HS patients in Canada (four patients from three families) [Tole et al. Br J Haematol 2020; 1C 91(3):486-496] and in a 4-months old male in China [Wu et al. Front Genet. 2021 Mar 18;12:652376. eCollection 2021]. In addition, in a recent review based on 13 selected studies with N=627 patients (which included the studies from

Tole et al and Wu et al.), the intronic ANK1 c.1405-9G>A variant was reported to be a ‘high frequency’ variant (N=8 patients) in HS [Yang L, et al. Clin Genet. 2022 Sep 7. doi: 10.1111/cge.14223]. A low hemoglobin (5.5 g/dL) was reported in the 4-month-old Chinese male, who carried this intronic variant [Wu et al. Front Genet. 2021 Mar 18;12:652376. eCollection 2021]. No details were provided on the severity of the disease in the four patients from Canada, who carried the same variant [Tole et al. Br J Haematol 2020; 191(3):486-496]. Two additional patients were published in 2023; one from China [Wang et al. Clin Genet. 2023 Jan;103(1):67-78.], and one from France [Lunati-Rozie A, et al. Br J Haematol. 2023 Mar 16. doi:

10.1111/bjh.18760. Online ahead of print]. The French patient was a 61-year-old male. We can confirm the ‘high frequency’ of this variant, as it occurred in two unrelated Austrian families (Table S4).

**Family F5: Index group patient P5**, male, 17-years-old, Central Europe, moderate HS phenotype

| **GENE** | **CHR** | **POS** | **IDS** | **REF** | **ALT** | **gnomAD _AF** | **gnomAD _AC** | **gnomAD _hom** | **Consequence** | **HGVSc (cDNA)** | **HGVSp (protein)** | **READS** | **SIFT** | **PolyPhen** | **CADD** |
| --- | --- | --- | --- | --- | --- | --- | --- | --- | --- | --- | --- | --- | --- | --- | --- |
| *ANK1* | 8 | 41573376 | . | C | T | NA | NA | NA | Intron | ENST00000289734.7:  c.1405-9G>A | ENSP00000289734.7:  p.Asp469GlyfsTer21 | 121 | NA | NA | 2.1 |

| **Method** | **LELY** | **αLEPRA** | **PMID** | **Pathogenicity (split)** |
| --- | --- | --- | --- | --- |
| Panel + WES | Heterozygous | Wild type | 32436265, 33868383  36071563, 36203343 36928866 | PVS1+PS2+PM2+PM4+PP4 |

Interpretation: The heterozygous intronic *ANK1* NM_000037.3:c.1405-9G>A variant was discovered in the patient (P5) via Panel and WES investigation. The healthy parents (laboratory data not shown) carry wt *ANK1* alleles. The patient carries a heterozygous *LELY* variant. Patients P4 (age 4 years) and P5 (age 17 years) have similar phenotypes, but whereas P4 is *LELY* wt, patient P5 carries a heterozygous *LELY* variant. The disease was rated moderate, and patient P5 developed gall stones at age 4 years and underwent cholecystectomy and total splenectomy at age 14 years.

This intronic *ANK1* variant causes the generation of a new splice-acceptor site, causing the incorporation of seven nucleotides to the canonical coding sequence, creating a frameshift and premature stop, leading to an erroneous truncated protein (Figure S2). More details in patient P4.

The *ANK1* c.1405-9G>A variant occurred *de novo* in P5 and is pathogenic (class 5) and the Mendelian genotype that causes his moderate HS phenotype.


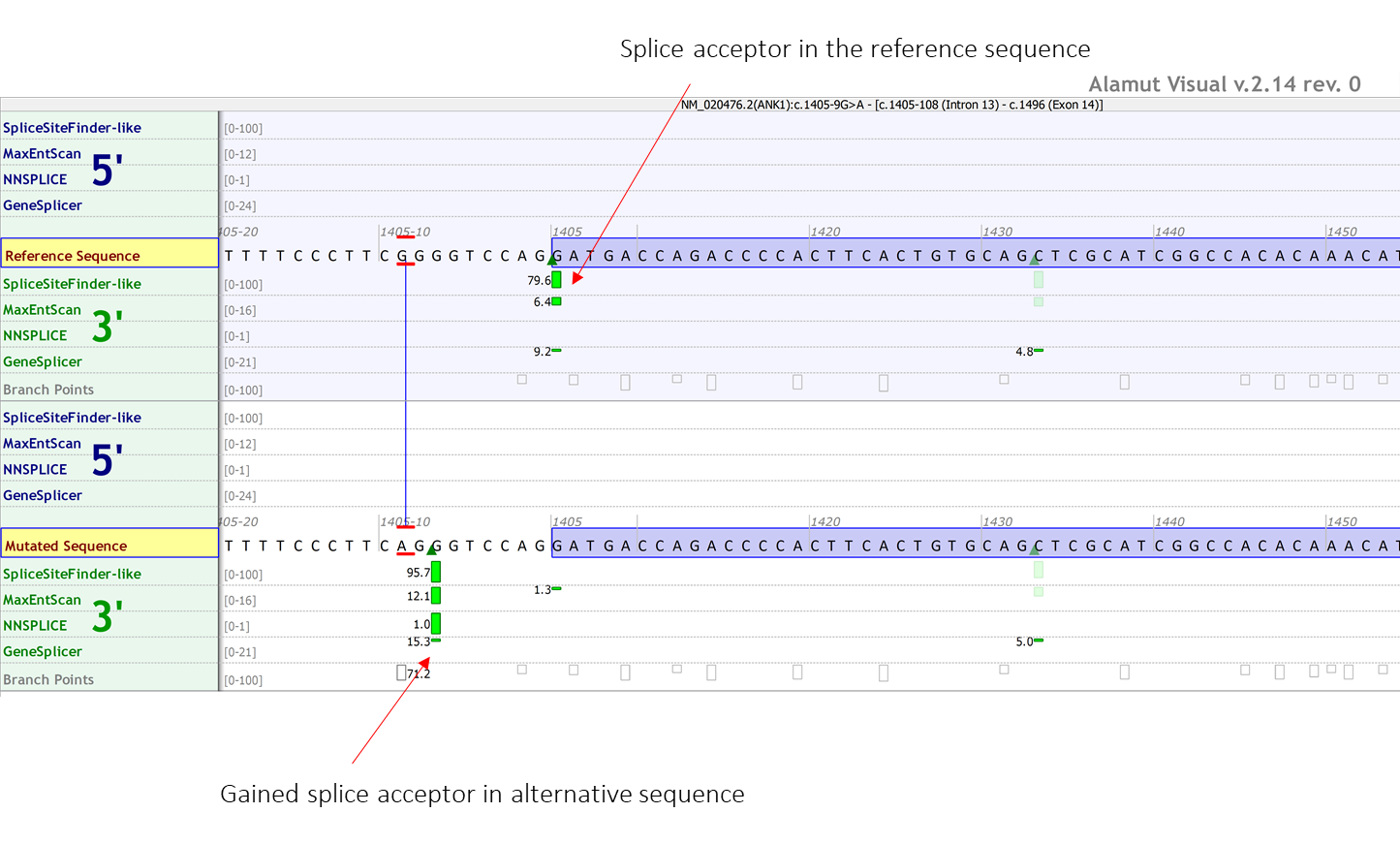


**Figure S2:** Splice site predicted effect of the intronic variant c.1405-9G>A (p.Asp469GlyfsTer21), which was identified in patients P4 (and her father P41 and sister P42) and P5 from unrelated families, visualized using Alamut visual v.2.14 rev. 0 (Sophia Genetics). The upper part represents the reference sequence. The lower part represents the mutated sequence. Highlighted in green, the expected splice site for both sequences. For the mutated sequence, there is an expected splice acceptor gained which would introduce 7 bp to the coding sequence leading to a frameshift.

**Family F6 (patients P6, P43): Index group patient P6**, male, 4-years-old, Central Europe, moderate HS phenotype

| **GENE** | **CHR** | **POS** | **IDS** | **REF** | **ALT** | **gnomAD _AF** | **gnomAD _AC** | **gnomAD _hom** | **Consequence** | **HGVSc (cDNA)** | **HGVSp (protein)** | **READS** | **SIFT** | **PolyPhen** | **CADD** |
| --- | --- | --- | --- | --- | --- | --- | --- | --- | --- | --- | --- | --- | --- | --- | --- |
| *ANK1* | 8 | 41571708 | . | AG | A | NA | NA | NA | Frameshift | ENST00000289734.7:  c.1765del | ENSP00000289734.7:  p.Leu589PhefsTer48 | 379 | NA | NA | 35 |

| **Method** | **LELY** | **αLEPRA** | **PMID** | **Pathogenicity (split)** |
| --- | --- | --- | --- | --- |
| Panel | Wild type | Wild type | - | PVS1+PM2+PM4+PP1+PP3+PP4 |

Interpretation: The heterozygous *ANK1* NM_000037.3:c.1765del (p.Leu589PhefsTer48) variant was discovered in the P6 via Panel investigation. The patient has a moderate HS phenotype (Table S1a). His mother (P43), who has a similar HS phenotype (Table S2a) carries the same variant. She underwent cholecystectomy at age 25-years. The variant was confirmed in both via Sanger sequencing. The healthy father carries wt *ANK1* alleles. The *LELY* in the patient was wt.

This *ANK1* variant causes a frameshift resulting in a premature stop codon, leading to an erroneous truncated protein or nonsense mediated mRNA decay. Moreover, there is a high CADD prediction score (i.e., 35) for the identified variant.

The variant per se affects a highly conserved L (leucine, within a coil sequence) in AR18 (AA 568-600) in the ANK1 membrane binding domain (Figure 1A) [Vallese et al. Nat Struct Mol Biol. 2022 Jul;29(7):706-718]. AR17-19 interact with the cytoplasmatic domain of Band 3 sub-complex III [Vallese et al. Nat Struct Mol Biol. 2022 Jul;29(7):706-718]. According to Xia et al., ARs 17-20 bind to Band 3 dimer [Xia et al. Nat Struct Mol Biol. 2022 Jul;29(7):698-705], and the variant affects this domain.

*ANK1* c.1765delC is pathogenic (class 5) and the autosomal dominant inherited Mendelian genotype that causes the moderate HS phenotype in P6 and P43. This variant is novel.

**Family F7 (patients P7, P44, P45): Index group patient P7**, female, 6-years-old, Central Europe, severe HS phenotype

| **GENE** | **CHR** | **POS** | **IDS** | **REF** | **ALT** | **gnomAD _AF** | **gnomAD _AC** | **gnomAD _hom** | **Consequence** | **HGVSc (cDNA)** | **HGVSp (protein)** | **READS** | **SIFT** | **PolyPhen** | **CADD** |
| --- | --- | --- | --- | --- | --- | --- | --- | --- | --- | --- | --- | --- | --- | --- | --- |
| *ANK1* | 8 | 41571688 | . | GCGG  GGAG  CCGC CC | G | NA | NA | NA | Frameshift | ENST00000289734.7:  c.1773_1785del | ENSP00000289734.7:  p.Gly592ThrfsTer41 | 509 | NA | NA | 36 |

| **Method** | **LELY** | **αLEPRA** | **PMID** | **Pathogenicity (split)** |
| --- | --- | --- | --- | --- |
| Panel | Heterozygous | Wild type | 29797310 | PVS1+PM2+PP1+PP3+PP4 |

Interpretation: The heterozygous *ANK1* NM_000037.3:c.1773_1785del (p.Gly592ThrfsTer41) variant was discovered in the 6-years old female (P7) via Panel investigation and was previously published by our group [Kager et al. Br J Haematol 2018;182:251-258]. She has a severe HS phenotype. Her father (P44), who developed gallstones at age 7-years and underwent splenectomy at age 12-years, and her 8-years old sister (P45) with moderate HS phenotype (Table S2a) carry the same variant, as confirmed via Sanger sequencing. The healthy mother carries wt *ANK1* alleles. P7 carries a heterozygous *LELY* variant.

The *ANK1* variant is pathogenic and causes a frameshift resulting in a premature stop codon, leading to an erroneous truncated protein or nonsense mediated mRNA decay. Moreover, there is a high CADD prediction score (i.e., 36) for the identified variant. The variant per se affects a highly conserved G (glycine, within a coil sequence) in AR18 (AA 568-600) (Figure 1A) [Vallese et al. Nat Struct Mol Biol. 2022 Jul;29(7):706-718]. AR1719 interact with the cytoplasmatic domain of Band 3 sub-complex III [Vallese et al. Nat Struct Mol Biol. 2022 Jul;29(7):706-718]. According to Xia et al., ARs 17-20 bind to Band 3 dimer [Xia et al. Nat Struct Mol Biol. 2022 Jul;29(7):698-705], and the variant affects this domain.

*ANK1* c.1773_1785del variant is pathogenic (class 5) and the autosomal dominant inherited Mendelian genotype that causes the severe and moderate HS phenotypes in P7, P44 and P45. The variant was previously published by our group [Kager et al. Br J Haematol 2018;182:251-258] and was not yet identified in other patients.

**Family F8 (patients P8, P46): Index group patient P8**, male, 15-years-old, Central Europe, moderate HS phenotype

| **GENE** | **CHR** | **POS** | **IDS** | **REF** | **ALT** | **gnomAD _AF** | **gnomAD _AC** | **gnomAD _hom** | **Consequence** | **HGVSc (cDNA)** | **HGVSp (protein)** | **READS** | **SIFT** | **PolyPhen** | **CADD** |
| --- | --- | --- | --- | --- | --- | --- | --- | --- | --- | --- | --- | --- | --- | --- | --- |
| *ANK1* | 8 | 41547745-  41552901 | . | NA | NA | NA | NA | NA | CNV | c.(2961+1_2962-  1)_(4103+1_41041)del | - | NA | NA | NA | NA |

| **Method** | **LELY** | **αLEPRA** | **PMID** | **Pathogenicity (split)** |
| --- | --- | --- | --- | --- |
| Panel + WES | Heterozygous | Wild type | - | PVS1+PM2+PP1+PP4 |

Abbreviations. NA, not annotated; ANK1, ankyrin 1; CNV, copy number variant, WES, whole exome sequencing.

Interpretation: The heterozygous *ANK1* deletion variant encompassing exons 27 to 33 (NM_000037.3:c.(2961+1_2962-1)_(4103+1_4104-1)del was discovered in the 15-years old male (P8) via Panel and WES investigation. He had moderate HS phenotype and underwent splenectomy at age 12 years. His father (P46), who was also splenectomized at age 12-years (Table S2a) carries the same variant. P8 carries a heterozygous *LELY* variant.

The *ANK1* variant is pathogenic (class 5) and it affects the spectrin binding domain (SBD, complete ZU5B and UPA regions, and a large part of the ZU5A region, Figure 1A). The central spectrin binding domain (SBD) of ANK1 has a modular structure that includes two tandem ZU5 domains (named for its homology to ZO-1, which is a tight junction-associated protein, and Unc5, which is a netrin receptor) and a UPA domain (a DCC [deleted in colorectal cancer]-binding motif-containing domain; named as the domain is conserved in UNC5, PIDD, and Ankyrins). The first ZU5 domain, ZU5A, is responsible for specific recognition and binding to repeats 14 and 15 of β-spectrin [Ipsaro & Mondragon, Blood. 2010;115(20):4093-4101]. The second ZU5 domain, ZU5-B, does not participate in spectrin binding directly, nor does it affect activity of ZU5-A binding via interactions [Yasanuga et al. J Mol Biol. 2012 Apr 6;417(4):336-50]. As there is structural similarity between ZU5B and the ZU5 domain found in the netrin receptor UNC5b supramodule (which also contains a UPA domain), it was suggested that UPA and ZU5B could interact with other domains in ankyrin and have regulatory functions [Yasanuga et al. J Mol Biol. 2012 Apr 6;417(4):336-50]. The UPA domain is suggested to mediate interactions between the ZU5B and death domains (DD) domains, the latter which is part of the ANK1 regulatory domain (Fig 1A) [Yasanuga et al. J Mol Biol. 2012 Apr 6;417(4):33650]. As the herein identified variant affects most of the SBD complex, it is likely that ANK1/β-spectrin binding is impaired.

*ANK1* c.(2961+1_2962-1)_(4103+1_4104-1)del CNV is the autosomal dominant inherited Mendelian genotype that causes the moderate HS phenotypes in P8 and P46. This is the first family identified to have moderate HS caused by a pathogenic CNV resulting in deletion of exons 27 to 33 in *ANK1*. A 14.8 kb *de novo* deletion, which results in loss of *ANK1* exons 17-24 was reported in a Canadian patient [Tole et al. Br J Haematol. 2020

Nov;191(3):486-496]. CNVs on 8p11.21 and HS with *ANK1* deletions in the context of contiguous gene syndromes are discussed below in patient P16.

**Family F9 (patients P9, P47): Index group patient P9**, male, 13-years-old, Central Europe, moderate HS phenotype

| **GENE** | **CHR** | **POS** | **IDS** | **REF** | **ALT** | **gnomAD _AF** | **gnomAD _AC** | **gnomAD _hom** | **Consequence** | **HGVSc (cDNA)** | **HGVSp (protein)** | **READS** | **SIFT** | **PolyPhen** | **CADD** |
| --- | --- | --- | --- | --- | --- | --- | --- | --- | --- | --- | --- | --- | --- | --- | --- |
| *ANK1* | 8 | 41552797 | . | C | A | NA | NA | NA | Stop gain | ENST00000289734.7:  c.3013G>T | ENSP00000289734.7:  p.Glu1005Ter | 791 | NA | NA | 42 |

| **Method** | **LELY** | **αLEPRA** | **PMID** | **Pathogenicity (split)** |
| --- | --- | --- | --- | --- |
| Panel | Heterozygous | Wild type | - | PVS1+PM2+PP1+PP3+PP4 |

Interpretation: The heterozygous *ANK1* NM_000037.3:c.3013G>T (p.Glu1005Ter) nonsense variant was discovered in the 13-years old male (P9) via Panel investigation. He has moderate HS phenotype (Table S1). His father (P47) was splenectomized at age 16-years for HS (Table S2a) and carries the same variant, as confirmed via Sanger sequencing. The healthy mother carries wt *ANK1* alleles. P9 carries a heterozygous *LELY* variant.

The heterozygous stop-gain variant results in premature cessation of translation of messenger RNA into protein, resulting in an erroneous truncated protein or nonsense mediated mRNA decay. Moreover, there is a very high CADD prediction score (i.e., 42) for the identified variant. The variant per se affects a highly conserved E (glutamic acid, last aa of a coil sequence) in the ZU5-A domain, which is responsible for specific recognition and binding of ANK1 to repeats 14 and 15 of β-spectrin (Figure 1A) [Ipsaro & Mondragon, Blood. 2010;115(20):4093-4101].

*ANK1* c.3013G>T variant is pathogenic (class 5) and the autosomal dominant inherited Mendelian genotype that causes the moderate HS phenotypes in P9 and P47. This variant is novel.

**Family F10 (patients P10, P48): Index group patient P10**, female, 3-years-old, Central Europe, moderate HS phenotype

| **GENE** | **CHR** | **POS** | **IDS** | **REF** | **ALT** | **gnomAD _AF** | **gnomAD _AC** | **gnomAD _hom** | **Consequence** | **HGVSc (cDNA)** | **HGVSp (protein)** | **READS** | **SIFT** | **PolyPhen** | **CADD** |
| --- | --- | --- | --- | --- | --- | --- | --- | --- | --- | --- | --- | --- | --- | --- | --- |
| *ANK1* | 8 | 41550348 | . | CA | C | NA | NA | NA | Frameshift | ENST00000289734.7:  c.3675del | ENSP00000289734.7:  p.Phe1225LeufsTer24 | 170 | NA | NA | 25.8 |

| **Method** | **LELY** | **αLEPRA** | **PMID** | **Pathogenicity (split)** |
| --- | --- | --- | --- | --- |
| WES | Wild type | Wild type | - | PVS1+PM2+PP1+PP3+PP4 |

Interpretation: The heterozygous *ANK1* NM_000037.3:c.3675delT (p.Phe1225LeufsTer24) variant was discovered in the 3-years old female (P10) via WES. She has a moderate HS phenotype (Table S1). Her mother (P48), who had undergone cholecystectomy and splenectomy at age 17-years (Table S2a) carries the same variant, as confirmed via Sanger sequencing. The healthy father carries wt *ANK1* alleles. P10 is *LELY* wt.

The heterozygous frameshift *ANK1* variant causes a premature stop codon, leading to an erroneous truncated protein or nonsense mediated mRNA decay. There is a moderately high CADD prediction score (i.e., 25.8) for the identified variant. It affects an F (phenylalanine, first aa of an alpha helix) [(https://www.dsimb.inserm.fr/respire/proteins/533)](https://www.dsimb.inserm.fr/respire/proteins/533) in the UPA domain of the SBD (Figure 1A). The UPA domain is suggested to mediate interactions between the ZU5B and the death domain (DD), the latter which is part of the ANK1 regulatory domain [Yasanuga et al. J Mol Biol. 2012 Apr 6;417(4):336-50].

*ANK1* c.3675delT variant is pathogenic (class 5) and the autosomal dominant inherited Mendelian genotype that causes the moderate HS phenotypes in P10 and P48. The variant is novel.

**Family F11: Index group patient P11**, female, 14-years-old, Central Europe, moderate HS phenotype

| **GENE** | **CHR** | **POS** | **IDS** | **REF** | **ALT** | **gnomAD _AF** | **gnomAD _AC** | **gnomAD _hom** | **Consequence** | **HGVSc (cDNA)** | **HGVSp (protein)** | **READS** | **SIFT** | **PolyPhen** | **CADD** |
| --- | --- | --- | --- | --- | --- | --- | --- | --- | --- | --- | --- | --- | --- | --- | --- |
| *ANK1* | 8 | 41550257 | . | AG | A | NA | NA | NA | Frameshift | ENST00000289734.7:  c.3766del | ENSP00000289734.7:  p.Leu1256CysfsTer6 | 120 | NA | NA | 35 |

| **Method** | **LELY** | **αLEPRA** | **PMID** | **Pathogenicity (split)** |
| --- | --- | --- | --- | --- |
| WES | Heterozygous | Wild type | - | PVS1+PM2+PM6+PP3+PP4 |

Interpretation: The heterozygous *ANK1* NM_000037.3:c.3766delC (p.Leu1256CysfsTer6) variant was discovered in the 14-years old female (P11) via WES. She has moderate HS phenotype (Table S1). Her healthy parents and her healthy brother (laboratory data not shown) all have *ANK1* wt, as confirmed via Sanger sequencing. P11 carries a heterozygote *LELY* variant.

The *de novo* *ANK1* variant causes a frameshift resulting in a premature stop codon, leading to an erroneous truncated protein or nonsense mediated mRNA decay. There is a high CADD prediction score (i.e., 35) for the identified variant. The variant affects a highly conserved L (leucine, at the end of a coil sequence) in the SBD. Both the heterozygous *ANK1* c.3675del variant in patient P10 (premature stop codon at aa 1248) and the heterozygous *ANK1* c.3766del variant in patient P11 (premature stop codon after amino acid 1261) affect the UPA domain (Figure 1A), and both have moderate HS phenotypes (Table S1a). The UPA domain is suggested to mediate interactions between the ZU5B and death domains (DD) domains, the latter which is part of the ANK1 regulatory domain [Yasanuga et al. J Mol Biol. 2012 Apr 6;417(4):336-50].

*ANK1* c.3766del variant is pathogenic (class 5) and the *de novo* Mendelian genotype that causes the moderate HS phenotype in P11. The variant is novel.

**Family F12 (patients P12, P49, P50): Index group patient P12**, female, 6-years-old, Central Europe, mild HS phenotype

| **GENE** | **CHR** | **POS** | **IDS** | **REF** | **ALT** | **gnomAD _AF** | **gnomAD _AC** | **gnomAD _hom** | **Consequence** | **HGVSc (cDNA)** | **HGVSp (protein)** | **READS** | **SIFT** | **PolyPhen** | **CADD** |
| --- | --- | --- | --- | --- | --- | --- | --- | --- | --- | --- | --- | --- | --- | --- | --- |
| *ANK1* | 8 | 41547849 | . | G | A | NA | NA | NA | Stop gain | ENST00000289734.7:  c.4000C>T | ENSP00000289734.7:  p.Arg1334Ter | 408 | NA | NA | 50 |

| **Method** | **LELY** | **αLEPRA** | **PMID** | **Pathogenicity (split)** |
| --- | --- | --- | --- | --- |
| Panel | Heterozygous | Wild type | 31400153  33868383 | PVS1+PS4moderate+PM2+PM6+PP1moderate+PP3+PP4 |

Interpretation: The heterozygous *ANK1* NM_000037.3:c.4000C>T (p.Arg1334Ter) nonsense stop gain variant was discovered in the 6-years old female (P12) via Panel investigation. She has mild HS phenotype (Table S1a). Her mother (P49) who underwent cholecystectomy and splenectomy at age 14years for HS (Table S2a) and her 14-years old brother (P50) who underwent cholecystectomy and splenectomy at age 12-years for moderate HS (Table S2a), both carry the same variant, as confirmed via Sanger sequencing. The healthy father carries wt *ANK1* alleles. Patient P12 carries a heterozygous *LELY* variant.

The heterozygous stop-gain variant affects an R (arginine, within a coil sequence) and results in premature cessation of translation of messenger RNA into protein, resulting in an erroneous truncated protein or nonsense mediated mRNA decay. Moreover, there is a very high CADD prediction score (i.e., 42) for the identified variant. The variant affects the UPA domain within the SBD (Figure 1A). The UPA domain is suggested to mediate interactions between the ZU5B and death domains (DD) domains, the latter which is part of the ANK1 regulatory domain [Yasanuga et al. J Mol Biol. 2012 Apr 6;417(4):336-50].

*ANK1* c.4000C>T variant is pathogenic (class 5) and the autosomal dominant inherited Mendelian genotype that causes the mild HS phenotype in P12 and the moderate HS phenotypes in P49 and P50. This variant has recently been described in an Italian family (four affected and investigated members; index patient 4-years-old female [2x transfusions], father, half-brother and aunt; all splenectomized in late adolescents except for the young index patient). The c.4000C>T variant was identified in all affected family members in combination with a rare single nucleotide polymorphism (rs142542271; MAF < 0.01) in *ANK1* of uncertain significance [Lazzareschi et al. Eur J Haematol. 2019 Nov;103(5):523-526]. In addition, the *ANK1* c.4000C>T variant was subsequently identified *de novo* in a 52-months-old female with HS (Hb 6.7 g/dL, reticulocytes 17.11%, total bilirubin 46.5 µmol/L) in China [Wu et al. Front Genet. 2021 Mar 18;12:652376].

**Family F13: Index group patient P13**, male, 20-years-old, Iran, mild HS phenotype

| **GENE** | **CHR** | **POS** | **IDS** | **REF** | **ALT** | **gnomAD _AF** | **gnomAD _AC** | **gnomAD _hom** | **Consequence** | **HGVSc (cDNA)** | **HGVSp (protein)** | **READS** | **SIFT** | **PolyPhen** | **CADD** |
| --- | --- | --- | --- | --- | --- | --- | --- | --- | --- | --- | --- | --- | --- | --- | --- |
| *ANK1* | 8 | 41546047 | . | GA | G | NA | NA | NA | Frameshift | ENST00000289734.7:  c.4164del | ENSP00000289734.7: p.Leu1389SerfsTer17 | 576 | NA | NA | 22.8 |

| **Method** | **LELY** | **αLEPRA** | **PMID** | **Pathogenicity (split)** |
| --- | --- | --- | --- | --- |
| Panel | Heterozygous | Wild type | - | PVS1+PM2+PM6+PP3+PP4 |

Interpretation: The heterozygous *ANK1* NM_000037.3:c.4164del (p.Leu1389SerfsTer17) variant was discovered in the P13 via Panel investigation. He has a mild HS phenotype (Table S1a). The patient is a refugee with no family members available for analysis. He carries a heterozygous *LELY* variant.

The *ANK1* variant causes a frameshift resulting in an early premature stop codon, leading to an erroneous truncated protein or nonsense mediated mRNA decay. The CADD prediction score (i.e., 22.8) is moderate. The variant per se affects an L (leucine, within a coil sequence) in the C-terminal region outside the regulatory domain of ANK1 (Figure 1A).

*ANK1* c.4164del is pathogenic (class 5) and causes the phenotype mild HS in P13. This variant is novel.

**Family F14 (patients P14, P51): Index group patient P14**, male, 4-years-old, Central Europe, moderate HS phenotype

| **GENE** | **CHR** | **POS** | **IDS** | **REF** | **ALT** | **gnomAD _AF** | **gnomAD _AC** | **gnomAD _hom** | **Consequence** | **HGVSc (cDNA)** | **HGVSp (protein)** | **READS** | **SIFT** | **PolyPhen** | **CADD** |
| --- | --- | --- | --- | --- | --- | --- | --- | --- | --- | --- | --- | --- | --- | --- | --- |
| *ANK1* | 8 | 41543669 | . | CTGTT | C | NA | NA | NA | Frameshift, splice region | ENST00000289734.7:  c.4387_4390del | ENSP00000289734.7:  p.Asn1463TrpfsTer17 | 635 | NA | NA | 35 |

| **Method** | **LELY** | **αLEPRA** | **PMID** | **Pathogenicity (split)** |
| --- | --- | --- | --- | --- |
| Panel | Wild type | Wild type | 29797310  31122244  31980736  33620149 | PVS1+PS4moderate+PM2+PP1+PP3 |

Interpretation: The heterozygous *ANK1* NM_000037.3:c.4387_4390del (p.Asn1463TrpfsTer17) variant was discovered in P14 via our HematologyPanel and first published by us in 2018 [Kager et al. Br J Haematol 2018;182:251-258]. He has moderate HS phenotype (Table S1a). His affected father (P51) underwent splenectomy for HS at age 22-years and cholecystectomy at age 32-years (Table S2a). The father carries the same pathogenic variant, as confirmed via Sanger sequencing. The healthy mother carries wt *ANK1* alleles. The patient is *LELY* wt.

This *ANK1* variant causes a frameshift resulting in a premature stop codon, leading to an erroneous truncated protein or nonsense mediated mRNA decay. Moreover, there is a high CADD prediction score (i.e., 35) for the identified variant. It affects an N (asparagine, within a coil sequence) in the death domain (DD) in the C-terminal regulatory domain of ANK1 (Figure 1A). The function of DD is currently unknown [Vallese et al. Nat Struct Mol Biol. 2022 Jul;29(7):706-718].

*ANK1* c.4387_4390del is pathogenic (class 5) and the autosomal dominant inherited Mendelian genotype that causes the phenotype moderate HS in P14 and his father P51 and was published by us earlier [Kager et al. Br J Haematol 2018;182:251-258]. This variant was subsequently reported in patients with HS in South Korea (two patients; one with moderate and one with severe phenotypes [Choi et al. Orphanet J Rare Dis. 2019 May 23;14(1):114]) and in China (38-years-old male [Qin et al. J Hum Genet. 2020 Apr;65(4):427-434] and a 9-years-old male [Xie et al. Mol Genet Genomic Med. 2021 Apr;9(4):e1577]). However, no details were provided on the phenotypes of the Chinese patients. The variant can be considered as ‘frequent’ variant (N=5 families) occurring in different ancestries (Central Europe, South- and East Asia) (Table S*4*).

**Family F15: Index group patient P15**, male, 17-years-old, Central Europe, moderate HS phenotype

| **GENE** | **CHR** | **POS** | **IDS** | **REF** | **ALT** | **gnomAD _AF** | **gnomAD _AC** | **gnomAD _hom** | **Consequence** | **HGVSc (cDNA)** | **HGVSp (protein)** | **READS** | **SIFT** | **PolyPhen** | **CADD** |
| --- | --- | --- | --- | --- | --- | --- | --- | --- | --- | --- | --- | --- | --- | --- | --- |
| *ANK1* | 8 | 41542137 | . | G | A | NA | NA | NA | Stop gain | ENST00000289734.7:  c.4462C>T | ENSP00000289734.7:  p.Arg1488Ter | 1522 | NA | NA | 36 |

| **Method** | **LELY** | **αLEPRA** | **PMID** | **Pathogenicity (split)** |
| --- | --- | --- | --- | --- |
| Panel | Heterozygous | Wild type | 12899723  30317022  31980736  36598564 | PVS1+PS4moderate+PM2+PM6+PP1+PP3 |

Interpretation: The heterozygous *ANK1* NM_000037.3:c.4462C>T (p.Arg1488Ter) nonsense stop gain variant was discovered in the 17-years old male (P15) via Panel investigation. He had moderate HS phenotype until he underwent splenectomy and cholecystectomy at age 16-years (Table S1a). The healthy parents and his healthy brother (laboratory data not shown) all carry wt *ANK1* alleles. P15 also carries a heterozygous *LELY* variant.

The stop-gain *ANK1* variant results in premature cessation of translation of messenger RNA into protein, resulting in an erroneous truncated protein or nonsense mediated mRNA decay. Moreover, there is a high CADD prediction score (i.e., 36) for the identified *ANK1* variant. It affects an R (arginine, within a coil region) in the C-terminal regulatory domain of ANK1 (Figure 1A).

The *ANK1* c.4462C>T variant is pathogenic (class 5) and the autosomal dominant inherited Mendelian genotype that causes the moderate HS phenotype in P15. This variant has been first described in a Czech patient (Variant Karlov) with HS [Özcan et al. Br J Haematol. 2003 Aug;122(4):669-77]. It was subsequently also identified in a family (13-years-old male with moderate HS phenotype and his father) in Taiwan [Lin et al. Clin Chim Acta. 2018 Dec;487:311-317] and in two Chinese patients (female, 15-years-old; male, 20-years-old) with HS [Qin et al. J Hum Genet. 2020 Apr;65(4):427434]. In addition, three adolescent Indians (one 14-years-old male, and two females aged 12 years) from three unrelated families with moderate HS phenotypes were recently reported to carry the p.Arg1488Ter variant [More TA et al. Mol Genet Genomics. 2023 Jan 4. doi: 10.1007/s00438-02201984-1]. The variant can be considered as ‘frequent’ variant occurring in different ancestries (Central Europe and East- and South Asia) (Table S4).

**Family F16: Index group patient P16**, male, 15-years-old, Chechen, moderate HS phenotype

| **GENE** | **CHR** | **POS** | **IDS** | **REF** | **ALT** | **gnomAD _AF** | **gnomAD _AC** | **gnomAD _hom** | **Consequence** | **HGVSc (cDNA)** | **HGVSp (protein)** | **READS** | **SIFT** | **PolyPhen** | **CADD** |
| --- | --- | --- | --- | --- | --- | --- | --- | --- | --- | --- | --- | --- | --- | --- | --- |
| *ANK1* | 8 | 41402861-  41535706 | . | NA | NA | NA | NA | NA | CNV | - | - | NA | NA | NA | NA |

| **Method** | **LELY** | **αLEPRA** | **PMID** | **Pathogenicity (split)** |
| --- | --- | --- | --- | --- |
| Panel + WES | Heterozygous | Wild type | - | Pathogenicity Score 1,50* |

*according to Riggs et al., Technical standards for the interpretation and reporting of constitutional copy number variants: a joint consensus recommendation of the American College of Medical Genetics and Genomics (ACMG) and the Clinical Genome Resource (ClinGen), Genet Med.

2020 February; 22(2): 245–257

Interpretation: The novel heterozygous *ANK1* CN variant (i.e., a 132.85 kb large deletion; arr 8p11.21(41402861_41535706)x1) was discovered in the 15-years old male (P16) via Panel and WES investigation; and confirmed via CytoScan^TM^ HD Array/Affimetrix^®^. He has moderate HS phenotype and developed gall stones at age 4-years and underwent endoscopic retrograde cholangiopancreatography (ERCP) with removal of the stones (Table S1a). The patient carries a heterozygous *LELY* variant. His healthy parents (laboratory data not shown) have *ANK1* wt alleles, as confirmed via CytoScan^TM^ HD Array/Affimetrix^®^. The *de novo* *ANK1* arr 8p11.21(41402861_41535706)x1 CNV affects the C-terminal region including parts of the regulatory domain (exons 39-43; Figure 1a). The deletion affects a total of 6 genes (i.e., *LOC102723729*, *GPAT4* [OMIM P608143], *NKX6-3* [OMIM P610772], *ANK1* exons 39-43, *MIR486-1* and *MIR486-2*) (Figure S3).

The novel pathogenic (class 5) CNV in *ANK1* arr8p11.21(41402861_41535706)x1 is the genotype that causes the moderate HS phenotype in P16.

Besides HS, patient’s P16 pathological phenotype also includes IgA deficiency and a cirrhosis of the left kidney, but normal kidney function (Figure S4). It is unknown if haploinsufficiency in either *LOC102723729*, *GPAT4* [OMIM P608143], *NKX6-3* [OMIM P610772], *MIR486-1* or *MIR486-2* can be associated with his immunological and non-hematological phenotypes.

*GPAT4* encodes glycerol-3-phosphate acyltransferase 4, which is highly expressed in kidney tubule cells

[(https://www.proteinatlas.org/ENSG00000158669-GPAT4/tissue/kidney)](https://www.proteinatlas.org/ENSG00000158669-GPAT4/tissue/kidney); and glycerol-3-phosphate acyltransferase activity has been shown to protect cells from lipotoxicity [Piccolis et al. Mol Cell. 2019 Apr 4;74(1):32-44.e8]. However, as only one kidney is affected, it is unlikely that *GPAT4* haploinsufficiency plays a role in the observed kidney pathology. *NKX6-3* encodes the transcription factor NK6 homeobox 3, which was reported to play a role in the development of the central nervous system, the gastrointestinal tract and the pancreas [Alentalo et al. 2006. Gene Expr Patterns. 2006 Jan;6(2):162-70].

Contiguous gene syndromes on chromosome 8p11.2 involving *ANK1* have been rarely reported; and the different phenotypes depend on the size and localization of the deletions. Pertinent details on the less than 20 cases reported are reviewed in Miya et al 2012 and Wang & Lai 2020.


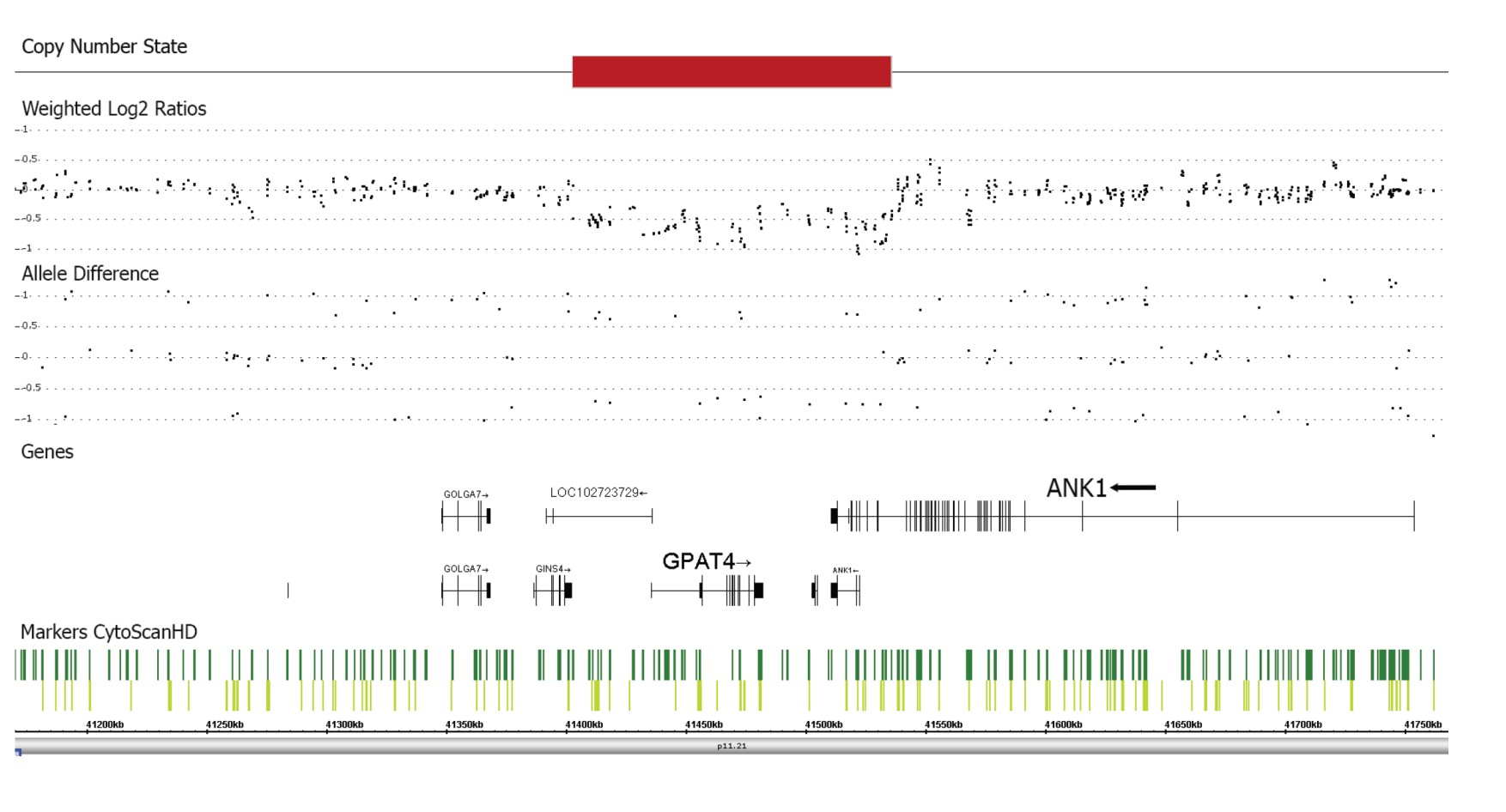


**Figure S3.** SNP/CGH array analysis of P16. CytoScan™ HD analysis showing a 132.85 kb deletion at chromosome 8p11.21 (chr8:4140286141535706) that included the *ANK1* gene (exons 39-43) as well as *LOC102723729*, *GPAT4*, *NKX6-3*, *MIR486-1*, and *MIR486-2*. The copy number state (copy number loss in red) and weighted log2 ratios are depicted in the upper part, the SNP allele pattern (allele difference) is shown in the middle part and genes are indicated by black vertical and horizontal bars in the lower part; SNP markers, light green vertical bars; oligo markers, dark green vertical bars are indicated in the bottom part of the figure.


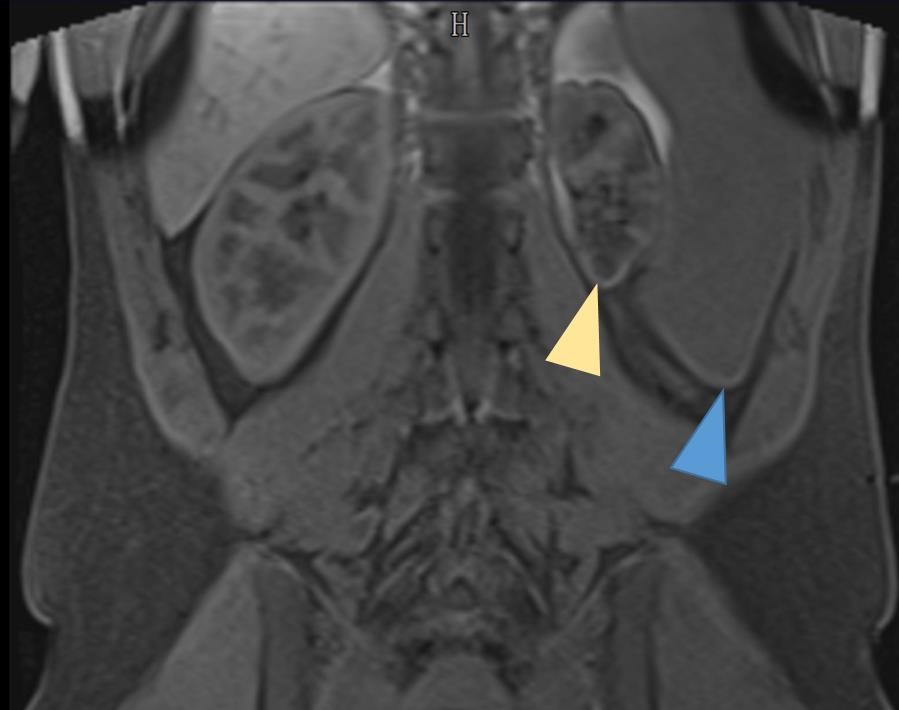


**Figure S4.** MRI of the abdomen in patient P16 showing enlarged spleen (blue triangle) and cirrhosis of the left kidney (yellow triangle).

**Summary ANK1 variants**

75% of index patients with causative *ANK1* variants had high impact alterations – that is, stop gain and frameshift variants (Figure S5).


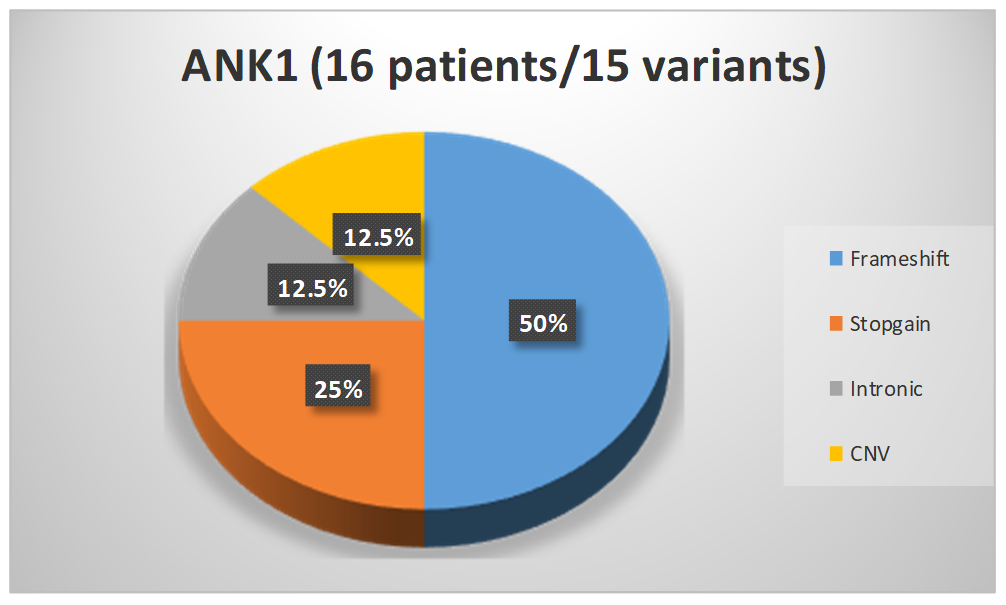


**Figure S5:** The 15 causative variants and the types of variation in *ANK1* identified in the 16 index group patients. Frameshift, N=8 (Index group patients P1, P3, P6, P7, P10, P11, P13, P14); stop-gain, N=4 (P2, P9, P12, P15); intronic variants, N=2 (same variant in P4, P5); CNVs, N=2 (P8, P16).

According to the annotation of pathogenicity based on the Standard Guidelines for the Interpretation of Sequence Variants [(https://pubmed.ncbi.nlm.nih.gov/25741868/](https://pubmed.ncbi.nlm.nih.gov/25741868/) and [https://pubmed.ncbi.nlm.nih.gov/31690835/)](https://pubmed.ncbi.nlm.nih.gov/31690835/), all 15 *ANK1* variants were classified as pathogenic. There was no difference in phenotypes among the different types of variants in *ANK1* (Figure S7).

**Figure S6A:** gDNA representation of *ANK1*. In red, newly described variants. In black, variants previously reported in the literature. The colours of the circles are described in the CADD vs MAF plot. The effect on the protein level is displayed in main figure 1.


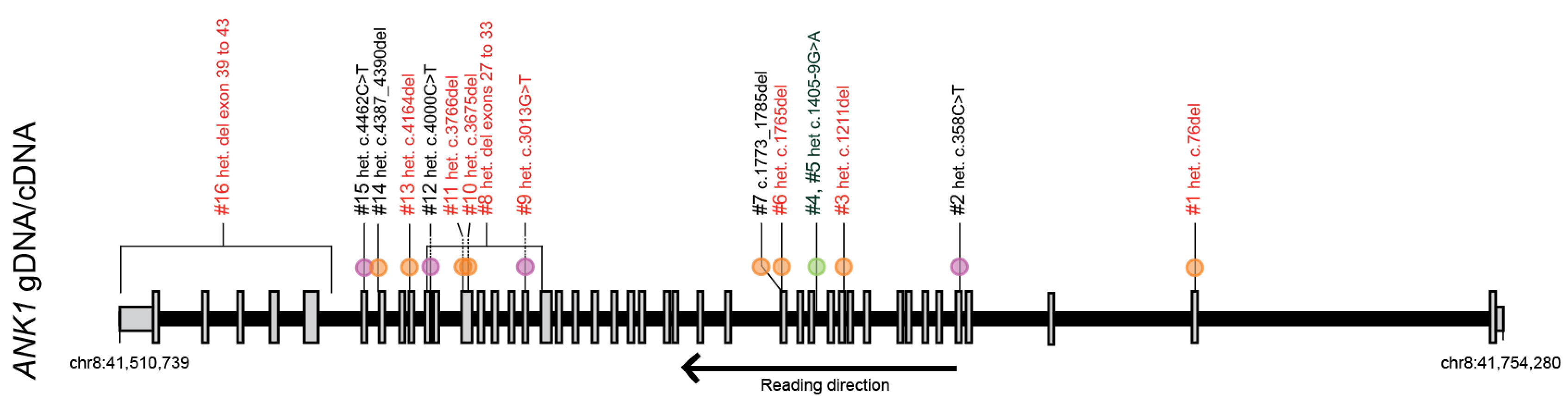


**Figure S6B:** CADD vs minor allele frequency (MAF) plot of all known *ANK1* sequence variants visualized by PopViz [Zhang et al. Bioinformatics 2018; 34: 4307–4309]. The horizontal axis shows the MAF scores and the vertical axis the CADD v1.3 ones. The specific types of the various sequence variants, which were collected from the gnomAD r2.0.2 database (https://gnomad.broadinstitute.org/), are color-coded, and the mutation of our patients are square-shaped. CNVs are not included in the CADD/MAF plot.


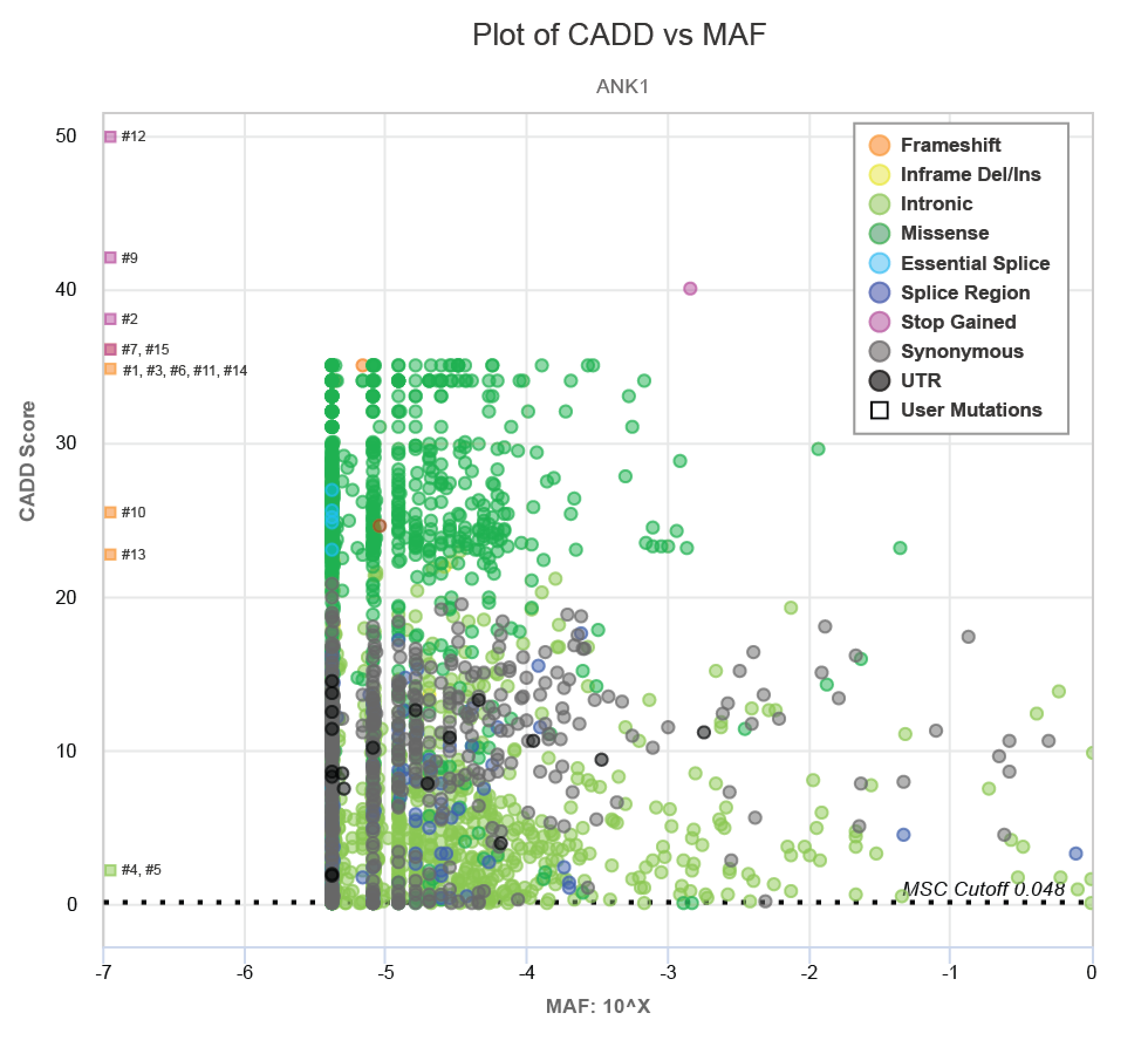


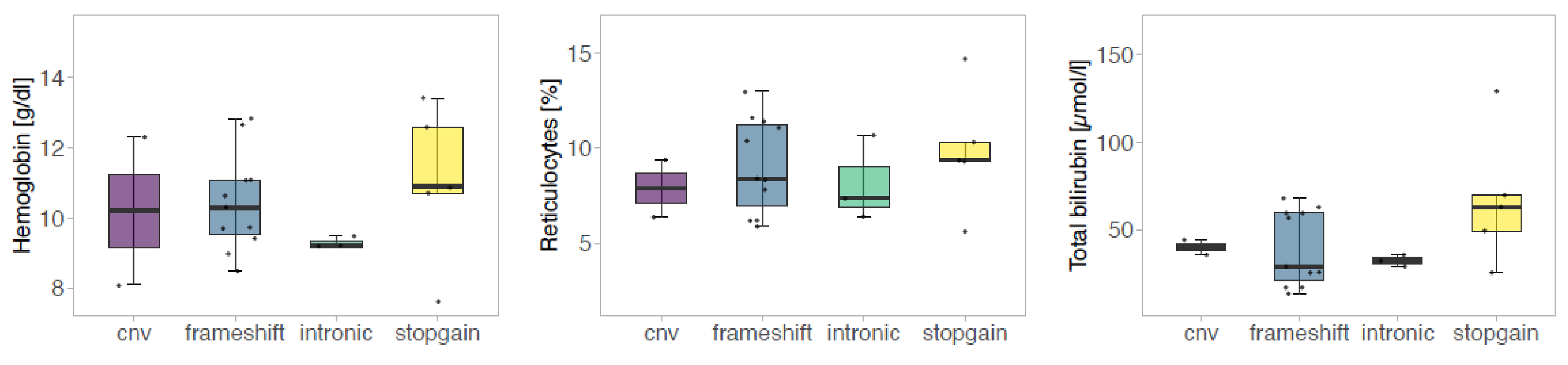


**Figure S7.** HS phenotypes and types of variants in *ANK1*.

The superhelix-shaped Ankyrin-1 is a major component of the ankyrin complex, which is responsible for anchoring the spectrin-based cytoskeleton of erythrocytes to the cell membrane.

Ankyrin-1 binds to the N- and C-domains of the membrane Rhesus protein RhCE via ankyrin repeats (AR) 1-5 [Vallese et al. Nat Struct Mol Biol. 2022 Jul;29(7):706-718]; and in patients P1 (mother P36) and P2 (father P37) the respective variants p.Asp26ThrfsTer11 [according to Vallese et al.

Nat Struct Mol Biol. 2022 Jul;29(7):706-718 this variant affects AR1] and p.Gln120Ter [according to Vallese et al. Nat Struct Mol Biol. 2022 Jul;29(7):706-718 this variant affects AR4] were predicted to affect the AR 1-5 domain (Figure 1A). Ankyrin-1 binds to protein 4.2, which stabilizes the cytoplasmic domain of band 3 dimer, via ARs 6-13 [Xia et al. Nat Struct Mol Biol. 2022 Jul;29(7):698-705]; and in patient P3 (mother P38, sisters P39 and P40) the p.Gly404AlafsTer2 variant affects the AR13 domain [Vallese et al. Nat Struct Mol Biol. 2022 Jul;29(7):706-718] (Figure 1A). ANK1 AR17-19 interact with the cytoplasmatic domain of Band 3 sub-complex IIII [Vallese et al. Nat Struct Mol Biol. 2022 Jul;29(7):706-718], and also according to Xia et al. ARs 17-20 interact with Band 3 dimer [Xia et al. Nat Struct Mol Biol. 2022 Jul;29(7):698-705]. The p.Leu589PhefsTer48 and p.Gly592ThrfsTer41variants discovered in patients P6 (mother P43) and P7 (father P44, sister P45) affect the AR17-20 domains (Figure 1A).

One intronic *ANK1* c.1405-9G>A p.Asp469GlyfsTer21 variant, which is predicted to affect the first amino acid of AR15 [Vallese et al. Nat Struct Mol Biol. 2022 Jul;29(7):706-718] (Figure 1A), was identified in the unrelated patients P4 (father P41 and sister P42) and P5. This variant was reported to be a ‘high frequency’ variant (Table S3) [Yang L, et al. Clin Genet. 2022 Sep 7. doi: 10.1111/cge.14223].

The central spectrin binding domain (SBD) of ANK1 has a modular structure that includes two tandem ZU5 domains (ZU5-A and ZU5-B) and a UPA domain [Ipsaro & Mondragon, Blood. 2010;115(20):4093-4101]. The ZU5-A domain is responsible for specific recognition and binding of ANK1 to repeats 14 and 15 of β-spectrin [Ipsaro & Mondragon, Blood. 2010;115(20):4093-4101]; and the p.Glu1005Ter variant, which was discovered in patient P9 (father P47) affects the ZU5-A domain. The UPA domain is suggested to mediate interactions between the ZU5B and death domains (DD) domains, the latter which is part of the ANK1 regulatory domain (Figure 1A) [Yasanuga et al. J Mol Biol. 2012 Apr 6;417(4):336-50]; and we have discovered variants affecting UPA; that is p.Phe1225LeufsTer24 in patient P10 (mother P48), p.Leu1256CysfsTer6 in patient P11, and p.Arg1334Ter in patient P12 (mother P49, brother P50).

CNVs in *ANK1* were identified in two patients, and one affected the ZU5A, ZU5B and UPA domains (P8 and his father P46), and one (P16) the Cterminal region of the *ANK1* gene (exons 39-43) as well as *LOC102723729*, *GPAT4*, *NKX6-3*, *MIR486-1*, and *MIR486-2*. All three patients had moderate phenotypes.

Patients with variants in the N-terminal region and membrane binding domains including the ARs had moderate (N=9) or severe (N=2) HS phenotypes, whereas patients with variants in the C-terminal region and spectrin binding domains including ZU5A, ZU5B, UPA, death domain (DD) and putative regulatory domain had either moderate (N=5) or mild (N=2) phenotypes (Figure S8).


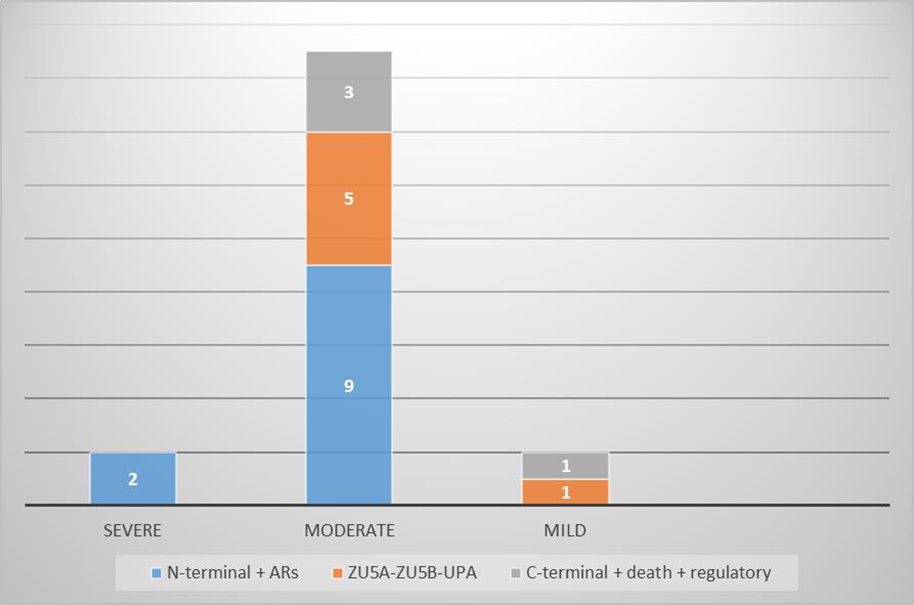


**Figure S8:** HS phenotypes according to location of the variants in *ANK1*. In 21 patients, adequate data was available. Severe phenotypes were identified in two patients with stop-gain (P2) and frameshift (P7) variants in ARs. Most patients had moderate phenotypes (N=9, N-terminal and ARs: P1, P3, P4, P5, P6, P36, P42, P43 and P45; N=5, ZU5A-ZU5B-UPA: P8, P9, P10, P11, P50; N=3, C-terminal DD and putative regulatory domain: P14, P15 and P16). Two patients had mild phenotypes. One with a stop-gain variant in the UPA domain (P12) and one with a frameshift variant in the C-terminal region outside the regulatory domain (P13) (see also Figure 1A).

Nine of the 15 variants in *ANK1* identified in our cohort are novel. Another three had been first reported by us in 2018 [Kager et al. Br J Haematol 2018;182:251-258], and two of these (family 2, p.Gln120Ter and family 14, p.Asn1463TrpfsTer17) were subsequently also identified by others in different ancestries [Svidnicki et al. Annals Hematol 2020; 99:955-962; More TA et al. Mol Genet Genomics. 2023 Jan 4. doi: 10.1007/s00438-02201984-1; Choi et al. Orphanet J Rare Dis. 2019 May 23;14(1):114; Qin et al. J Hum Genet. 2020 Apr;65(4):427-434; Xie et al. Mol Genet Genomic Med. 2021 Apr;9(4):e1577]. The table below describes pertinent details of *ANK1* variants identified in our cohort, which have been reported in more than one family (Table S4).

**Table S4.** Disease-causing *ANK1* variants identified in our study which are described in ≥ 2 families.

| **Family No. in our cohort** | **cDNA change** | **Protein change** | **Consequence** | **No. of patients/families** | **Ancestries** | **PMID** |
| --- | --- | --- | --- | --- | --- | --- |
| 2 | c.358C>T | p.Gln120Ter | Stop gain | 4/3 | Central Europe, South America, South Asia | 32266426  36598564 |
| 4 and 5 | c.1405-9G>A | p.Asp469GlyfsTer21 | Intron | 11(14^^[[7]](#footnote-8)^^) /6(?^1^) | Central Europe, West Europe, North America, East Asia | 32436265  33868383  36203343  36928866  36071563^1^ |
| 12 | c.4000C>T | p.Arg1334Ter | Stop gain | 8/3 | Central Europe, South Europe, East Asia | 31400153  33868383 |
| 14 | c.4387_4390del | p.Asn1463TrpfsTer17 | Frameshift | 6/5 | Central Europe, South Asia, East Asia | 31122244  31980736  33620149 |
| 15 | c.4462C>T | p.Arg1488Ter | Stop gain | 9/8 | Central Europe, South Asia, East Asia | 12899723  30317022  31980736  36598564 |

**Index patients (P) and their families (F) with causative variants in *SLC4A1***

**Family F17 (patients P17, P52): Index group patient P17**, female, 4-years-old, Central Europe, mild HS phenotype

| **GENE** | **CHR** | **POS** | **IDS** | **REF** | **ALT** | **gnomAD _AF** | **gnomAD _AC** | **gnomAD _hom** | **Consequence** | **HGVSc (cDNA)** | **HGVSp (protein)** | **READS** | **SIFT** | **PolyPhen** | **CADD** |
| --- | --- | --- | --- | --- | --- | --- | --- | --- | --- | --- | --- | --- | --- | --- | --- |
| *SLC4A1* | 17 | 42336583 | . | TG | T | NA | NA | NA | Frameshift | ENST00000262418.6:  c.823del | ENSP00000262418.6:  p.His275ThrfsTer22 | 437 | NA | NA | 17.3 |

| **Method** | **LELY** | **αLEPRA** | **PMID** | **Pathogenicity (split)** |
| --- | --- | --- | --- | --- |
| Panel | Wild type | Wild type | - | PVS1+PM2+PP1+PP3+PP4 |

Abbreviations. NA, not annotated; *SLC4A1*, Solute Carrier Family 4 Member 1 (Diego Blood Group).

Interpretation: The novel heterozygous *SLC4A1* c.823delC variant was discovered in the P17 via our Hematology-Panel. She has mild HS phenotype (Table S1b). Her mother (P52) also has a mild HS phenotype (Table S2b) and carries the same pathogenic variant, as confirmed via Sanger sequencing. The healthy father carries wt *SLC4A1* alleles. The patient is *LELY* wt.

This *SLC4A1* variant is pathogenic (class 5) and causes a frameshift resulting in a premature stop codon, leading to an erroneous truncated protein or nonsense mediated mRNA decay. However, there is a rather low CADD prediction score (i.e., 17.25) for the identified variant. The variant per se affects an H (histidine, within a coil region) in the N-terminal cytoplasmic domain (cdb3) [Xia et al. Nat Struct Mol Biol. 2022 Jul;29(7):698-705; Kalli AC, Reithmeier RAF. Front Physiol. 2022 Feb 25;13:817945] (Figure 1B). According to Vallese et al. the variant is localized in the band 3-III (cytosolic Band 3-glycoprotein A [GPA] dimer) domain [Vallese et al. Nat Struct Mol Biol. 2022 Jul;29(7):706-718]. Vallese et al. report that aa R295 and D297 in band 3-III interact with D748 and K755 of ankyrin 1; and the c.823delC variant causes a stop codon at aa 296 in band 3-III [Vallese et al. Nat Struct Mol Biol. 2022 Jul;29(7):706-718].

*SLC4A1* c.823delC is the autosomal dominant inherited Mendelian genotype that causes a mild HS phenotype in P17 and her mother P52. This variant is novel.

**Family F18 (patients P18, P53): Index group patient P18**, male, 19-years-old, Central European, moderate HS phenotype

| **GENE** | **CHR** | **POS** | **IDS** | **REF** | **ALT** | **gnomAD _AF** | **gnomAD _AC** | **gnomAD _hom** | **Consequence** | **HGVSc (cDNA)** | **HGVSp (protein)** | **READS** | **SIFT** | **PolyPhen** | **CADD** |
| --- | --- | --- | --- | --- | --- | --- | --- | --- | --- | --- | --- | --- | --- | --- | --- |
| *ANK1* | 8 | 41575124 | . | C | T | 0,000007 | 2 | 0 | Missense, Splice region | ENST00000289734.7:  c.1303G>A | ENSP00000289734.7:  p.Val435Met | 826 | Dele- terious | Probably _damaging | 34 |
| *SLC4A1* | 17 | 42335366 | . | C | G | NA | NA | NA | Missense | ENST00000262418.6:  c.1270G>C | ENSP00000262418.6:  p.Gly424Arg | 1157 | Dele- terious | Probably _damaging | 33 |

| **Gene** | **Method** | **LELY** | **αLEPRA** | **PMID** | **Pathogenicity (split)** |
| --- | --- | --- | --- | --- | --- |
| *ANK1* | Panel | Heterozygous | Wild type | - | PM2+PP3+PP4+BS2+BS4+BP5 |
| *SLC4A1* | Panel | Heterozygous | Wild type | - | PM2+PP1+PP2+PP3+PP4 |

Interpretation: P18 carries one likely pathogenic and one potential modifier variant in HS candidate genes, which were identified via our HematologyPanel; that is a novel heterozygous *SLC4A1* c.1270G>C missense variant and a novel heterozygous c.1303G>A missense variant in *ANK1*. The *ANK1* variant has a higher CADD score (34) when compared to the *SLC4A1* variant (CADD score 33). Both variants are predicted to be deleterious in SIFT and probably damaging in PolyPhen. Segregation analysis helped us to assess which variant may be causative.

The mother (P53) who has a mild HS phenotype (Table S2b) carries the likely pathogenic heterozygous *SLC4A1* c.1270G>C missense variant and is *ANK1* wt. The healthy father and healthy sister both carry wt *SLC4A1* alleles and both carry the *ANK1* c.1303G>A missense variant in a heterozygote state (Figure S9). The patient also carries a heterozygous *LELY* variant.

Interestingly, the patient has a more severe (i.e., moderate) HS phenotype (Table S1b) compared to the mother (mild HS phenotype; Table S2b).

The *SLC4A1* variant is likely pathogenic and affects a conserved G (glycine in a coil sequence) in the first transmembrane segment (TM1 or α13) of the band 3 transmembrane core domain in the N-terminal cytoplasmic region (mdb3) [Xia et al. Nat Struct Mol Biol. 2022 Jul;29(7):698-705; Kalli AC, Reithmeier RAF. Front Physiol. 2022 Feb 25;13:817945] (Figure 2A). Xia et al. report that F423 is a substrate binding site, and this is adjacent to the identified variant; i.e., pG424R.

The per se non-causative *ANK1* c.1303G>A variant affects ankyrin 1 repeat AR13. Vallese et al. report ankyrin AR13-24 binding to the cytoplasmic domain of band 3 [Vallese F, et al. Nat Struct Mol Biol. 2022 Jul;29(7):706-718]. However, the likely pathogenic *SLC4A1* c.1270G>C missense variant in patient P18 affects TM1, and not the cytoplasmic Band 3 domain directly. Further studies are necessary to prove that the combination of *SLC4A1* and *ANK1* variants results in the more severe HS phenotype in patient P18.

In summary, *SLC4A1* c.1270G>C is the likely pathogenic (class 4) autosomal dominant inherited Mendelian genotype that causes the HS phenotype in P18 and his mother P53. It is unknown if *ANK1* c.1303G>A variant in combination with *LELY* heterozygote (as in P18) may be a HS phenotype modifier.


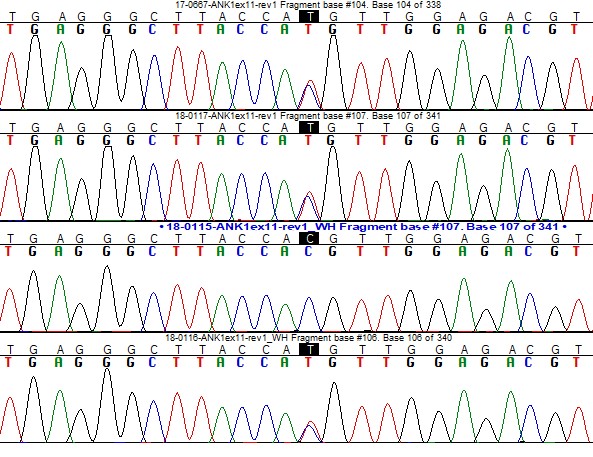

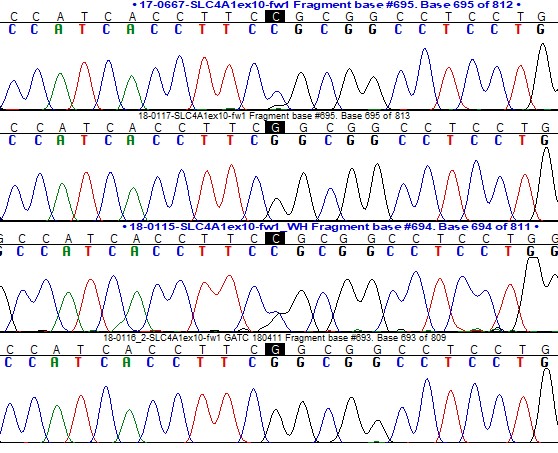


**Figure S9:** Segregation analysis in F18. **Left:** *ANK1 mis*sense variant segregation. Starting from top to bottom of the chromatograms: Index patient (HET), sister (HET), mother (WT) and father (HET). **Right:** *SLC4A1 mis*sense variant segregation. Starting from top to bottom of the chromatograms: Index patient (HET), sister (WT), mother (HET) and father (WT).

**Family F19 (patients P19, P54, P55, P56, P57): Index group patient P19,** male, 9-years-old, Turkish, severe HS phenotype (homozygous

*SLC4A1*variant; Band 3^Null^ Vienna)

| **GENE** | **CHR** | **POS** | **IDS** | **REF** | **ALT** | **gnomAD _AF** | **gnomAD _AC** | **gnomAD _hom** | **Consequence** | **HGVSc (cDNA)** | **HGVSp (protein)** | **READS** | **SIFT** | **PolyPhen** | **CADD** |
| --- | --- | --- | --- | --- | --- | --- | --- | --- | --- | --- | --- | --- | --- | --- | --- |
| *SLC4A1* | 17 | 42335028 | . | G | T | NA | NA | NA | Stop gain | ENST00000262418.6:  c.1430C>A | ENSP00000262418.6:  p.Ser477Ter | NA | NA | NA | 37 |

| **Method** | **LELY** | **αLEPRA** | **PMID** | **Pathogenicity (split)** |
| --- | --- | --- | --- | --- |
| Sanger sequencing | Heterozygous | Wild type | 27718309 | PVS1+PM2+PP2+PP3+PP4 |

Interpretation: P19 carries a homozygous *SLC4A1* c.1430C>A stop gain variant, which results in complete loss of Band 3 (Band 3^Null^ Vienna) [Kager L, et al. Pediatr Blood Cancer. 2017 Mar;64(3)]. Diagnostics, clinical presentation (transfusion-dependent chronic hemolytic anemia, dyserythropoiesis, and complete distal renal tubular acidosis (dRTA)), treatments and course until the age of five years were reported by us in detail previously [Kager et al. Pediatr Blood Cancer. 2017 Mar;64(3)]. Both his consanguine parents (P54, P55) as well as his brother (P56) and sister (P57) have subclinical spherocytosis (Table S2b) and all four carry the *SLC4A1* c.1430C>A variant in a heterozygote state. The amount of band 3 present in the red cell membranes of the parents was about 60% that in the normal control membranes [Kager et al. Pediatr Blood Cancer. 2017 Mar;64(3)].

The variant per se affects a S (Serine) and affects the third transmembrane segment (TM3; α15) of the band 3 transmembrane core domain (Figure 1B) [Xia et al. Nat Struct Mol Biol. 2022 Jul;29(7):698-705; Kalli AC, Reithmeier RAF. Front Physiol. 2022 Feb 25;13:817945]. TM3 together with TM10 may provide a binding site for substrate anions [Xia et al. Nat Struct Mol Biol. 2022 Jul;29(7):698-705]. The patient also carries a heterozygous *LELY* variant.

The *SLC4A1* c.1430C>A variant is pathogenic (class 5) and the autosomal dominant inherited Mendelian genotype that causes the severe HS phenotype in patient P19, who is homozygous for this variant; and the subclinical HS phenotype in his heterozygous family members (P54 to P57).

Follow up of the clinical course of P19 since the first publication [Kager et al. Pediatr Blood Cancer. 2017 Mar;64(3)]: At the age of six years, a laparoscopic total splenectomy was performed. In contrast to a female patient from Coimbra (Band 3 null^COIMBRA^), who carries a homozygous V488M SLC4A1 variant [Ribeiro ML, et al. Blood. 2000 Aug 15;96(4):1602-4.] and became transient transfusion independent for 9 years after splenectomy at 3 6/12 years, maintaining hemoglobin levels at 8.5–9.1 g/dl [Kager L, et al. Pediatr Blood Cancer. 2017 Mar;64(3)], patient P19 remained transfusion dependent. However, the transfusion frequency was significantly reduced from q3 to 4 weeks to q6 to 8 weeks after splenectomy. Because of permanent increase of thrombocytes > 1000 G/L, he receives oral low-dose aspirin since splenectomy. A peripheral blood smear after splenectomy shows a high number of spherocytes, dysmorphic erythrocytes and increased number of thrombocytes (Figure S10). Relative PK deficiency has been observed in HS RBCs [Andres et al. Br J Haematol. 2019 Nov;187(3):386-395]. In a band 4.1 knock-out HS mouse model, treatment with the pyruvate kinase activator mitapivat ameliorated anemia and prevented iron-overload and was not inferior to splenectomy [Matte et al. Curr Opin Hematol. 2023 Feb 10]; and if also successfully tested in humans, this treatment might be a future therapeutic option for this patient. A hematopoetic stem cell transplantation (HSCT) is not considered for different reasons.


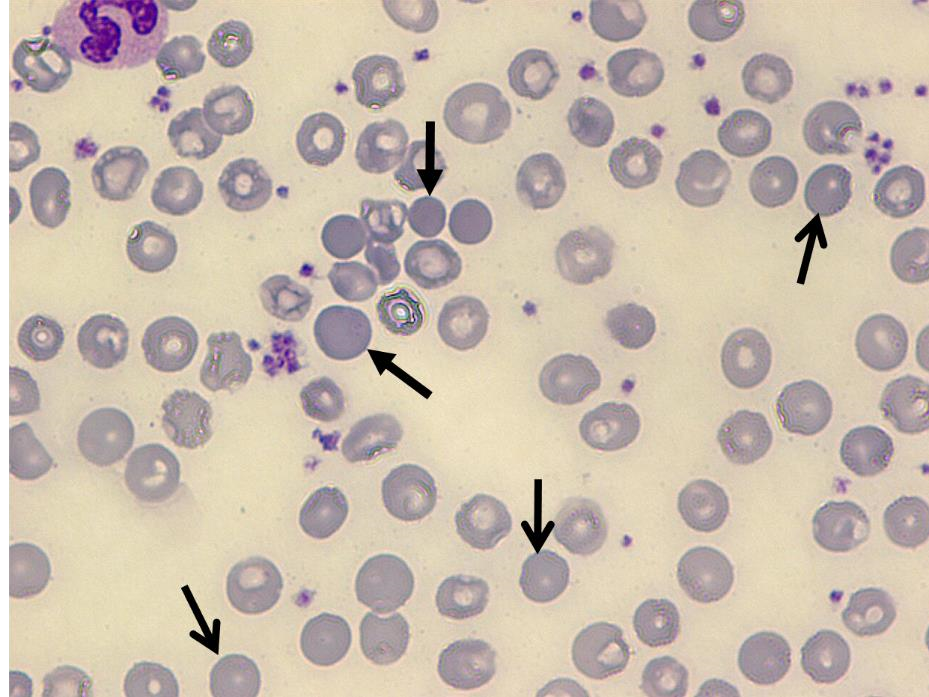


**Figure S10.** Peripheral blood smear in P19 after splenectomy (May-Gruenwald-Giemsa-stained). There are typical spherocytes (arrow) and numerous abnormal RBCs (open arrow), which may represent intermediates between spherocytes and normal RBCs. There is also an increased number of thrombocytes.

**Family F20 (patients P20, P58): Index group patient P20**, female, 13-years-old, Central European, mild HS phenotype

| **GENE** | **CHR** | **POS** | **IDS** | **REF** | **ALT** | **gnomAD _AF** | **gnomAD _AC** | **gnomAD _hom** | **Consequence** | **HGVSc (cDNA)** | **HGVSp (protein)** | **READS** | **SIFT** | **PolyPhen** | **CADD** |
| --- | --- | --- | --- | --- | --- | --- | --- | --- | --- | --- | --- | --- | --- | --- | --- |
| *SLC4A1* | 17 | 42334876 | . | G | A | 0,000032 | 1 | 0 | Missense | ENST00000262418.6:  c.1468C>T | ENSP00000262418.6:  p.Arg490Cys | 200 | Dele- terious | Probably _damaging | 26.6 |

| **Method** | **LELY** | **αLEPRA** | **PMID** | **Pathogenicity (split)** |
| --- | --- | --- | --- | --- |
| Panel | Wild type | Wild type | 9233560  29572776  36203343 | PS1+PS3+PM1+PM6+PP3+PP4 |

Interpretation: The heterozygous *SLC4A1* c.1468C>T missense variant was discovered in P20 via our Hematology-Panel. She has mild HS phenotype (Table S1b) but developed gall stones at the age of 10 years. Her father (P58) has moderate HS phenotype and underwent cholecystectomy at age 45 years (Table S2b) and carries the same pathogenic variant, as confirmed via Sanger sequencing. The healthy mother carries wt *SLC4A1* alleles. The patient is *LELY* wt.

The *SLC4A1* variant has a moderate CADD prediction score (i.e., 26.6). The variant per se affects a highly conserved R (Arginine) at the fourth transmembrane segment (TM4; α16) of the band 3 transmembrane core domain (Figure 1B) [Xia et al. Nat Struct Mol Biol. 2022 Jul;29(7):698-705; Kalli & Reithmeier. Front Physiol. 2022 Feb 25;13:817945]. Functional investigations provide evidence that the mutant R490C construct does not traffic into the plasma membrane but is retained in the endoplasmic reticulum. In addition, the variant construct impairs the localization of the wild type protein into the plasma membrane [Dhermy et al. Mol Membr Biol. 1999 Oct-Nov;16(4):305-12].

*SLC4A1* c.1468C>T is pathogenic (class 5) and the autosomal dominant inherited Mendelian genotype that causes the HS phenotype in P20 and her father P58.

This variant was first identified in six HS patients (ancestry of the patients was not provided, but the analyses were done in France) in three different families by Dhermy et al. and named Band 3^Bicetre I^ (rs1398477044) [Dhermy et al. Br J Haematol. 1997 Jul;98(1):32-40]. It was suggested that *SLC4A1* c.1468C>T, p.Arg490Cys, rs1398477044 represent a ‘hot spot’ for mutation, because arginine is encoded by the codon CGT, and CG dinucleotides having been implicated in a large number of recurrent point mutations [Dhermy et al. Br J Haematol. 1997 Jul;98(1):32-40]. The rs1398477044 variant was also identified in two HS patient in China [Wang et al. Sci China Life Sci. 2018 Aug;61(8):947-953; Wang et al. Clin Genet. 2023 Jan;103(1):6778]. Yet, at least a total of ten patients from different ancestries have been documented to have HS caused by the *SLC4A1* c.1468C>T variant (Table S5). Moreover, a pathogenic variant that affect the same codon (i.e., c.1469G>A, p.Arg490His; rs1598299485) has been identified in patients with HS from Japan [Yamamoto et al. Hum Genome Var. 2022 Jan 12;9(1):1] and China [Shen et al. BMC Med Genet. 2019 May 24;20(1):90].

## Family F21 (patients P21, P59, P60): Index group patient P21, female, 6-years-old, Central European, mild HS phenotype

| **GENE** | **CHR** | **POS** | **IDS** | **REF** | **ALT** | **gnomAD _AF** | **gnomAD _AC** | **gnomAD _hom** | **Consequence** | **HGVSc (cDNA)** | **HGVSp (protein)** | **READS** | **SIFT** | **PolyPhen** | **CADD** |
| --- | --- | --- | --- | --- | --- | --- | --- | --- | --- | --- | --- | --- | --- | --- | --- |
| *SLC4A1* | 17 | 42330512 | . | C | T | NA | NA | NA | Missense | ENST00000262418.6:  c.2285G>A | ENSP00000262418.6:  p.Ser762Asn | 664 | Dele- terious | Probably _damaging | 23.3 |

| **Method** | **LELY** | **αLEPRA** | **PMID** | **Pathogenicity (split)** |
| --- | --- | --- | --- | --- |
| Panel | Wild type | Wild type | - | PM2+PM5+PP1+PP3+PP4 |

Interpretation: The heterozygous *SLC4A1* c.2285G>A missense variant was discovered in P21 via our Hematology-Panel. She has mild HS phenotype (Table S1b). Her mother (P59) has moderate HS phenotype and received two RBC transfusions after delivery (Table S2b) and carries the same pathogenic variant, as confirmed via Sanger sequencing. Her 10-years old brother (P60) carries the same *SLC4A1*variant and has mild HS phenotype (Table S2b). The healthy father refused analyses. The patient is *LELY* wt.

The *SLC4A1* variant has a moderate CADD prediction score (i.e., 23.3). The variant per se affects a highly conserved S (Serine) at the eleventh transmembrane segment (TM11, α25) of the band 3 transmembrane core domain (Figure 1B) [Xia et al. Nat Struct Mol Biol. 2022 Jul;29(7):698-705; Kalli AC, Reithmeier RAF. Front Physiol. 2022 Feb 25;13:817945]. Guizouarn et al. reported in 2011 a different missense change at the same amino acid residue (p.Ser762Arg) in a family with hereditary stomatocytosis / cryohydrocytosis [Guizouarn et al. Br J Haem. 2011 Mar;152(5):655-64]. They also did extensive electrophysiological studies showing functional abnormalities in Xenopus laevis oocytes expressing this mutant.

*SLC4A1* c.2285G>A is likely pathogenic and the autosomal dominant inherited Mendelian genotype that causes the HS phenotype in P21, her brother P60 and her mother P59.

**Summary *SLC4A1* Variants:**

The most common disease-causing genetic alterations in *SLC4A1* were missense variants (Figure S11).


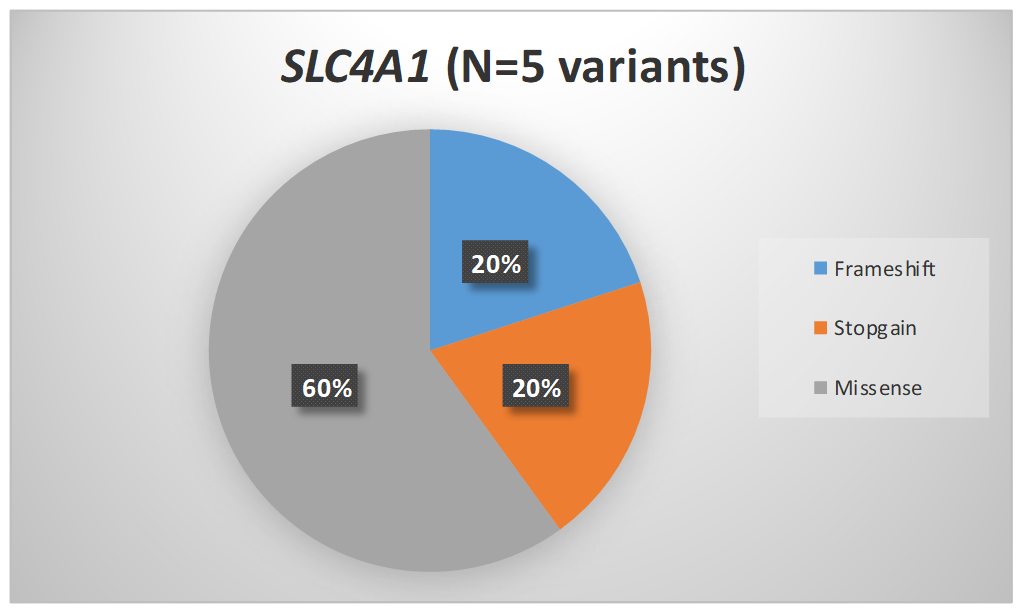


**Figure S11:** The 5 causative variants and the types of variation in *SLC4A1* identified in the index group patients. Missense, N=3 (patients P18, P20, P21); Frameshift, N=1 (P17); stop gain, N=1 (P19).

According to the annotation of pathogenicity based on the Standard Guidelines for the Interpretation of Sequence Variants [(https://pubmed.ncbi.nlm.nih.gov/25741868/)](https://pubmed.ncbi.nlm.nih.gov/25741868/), two missense variants were classified as likely pathogenic (c.1270G>C and c.2285G>A) all others as pathogenic.

Band 3 is a dimer and contains a membrane domain (mdb3) and a cytoplasmic domain (cdb3) [Xia et al. Nat Struct Mol Biol. 2022 Jul;29(7):698-705].

Interestingly, 4/5 variants affected the band 3 **transmembrane core domains** (each one in TM1, TM3, TM4, and TM11), but none affected the transmembrane gate domains or short helical regions joining the TM segments. This is in-line with Xia et al, who recently stated that from 21 reported disease mutation affecting either the core or gate domains of human band 3, 14 were localized within core domains and only seven within gate domains [Xia et al. Nat Struct Mol Biol. 2022 Jul;29(7):698-705]. One pathogenic frameshift variant affected the **helical cytoplasmic domain**. **Missense variants** were the most prevalent types (3/5), which is in-line with previous reports [Wang et al. Clin Genet. 2023 Jan;103(1):67-78; van Vuren A et al. Hemasphere. 2019 Aug 7;3(4):e276]. The localization of these missense variants in a 3D model of band3 is provided in Figure S12. Patients with missense variants had higher reticulocyte and bilirubin values compared to patients with stop-gain variants (Figure S14). One of the herein identified variants in *SLC4A1* was reported in ≥ 2 families (see Table S5).


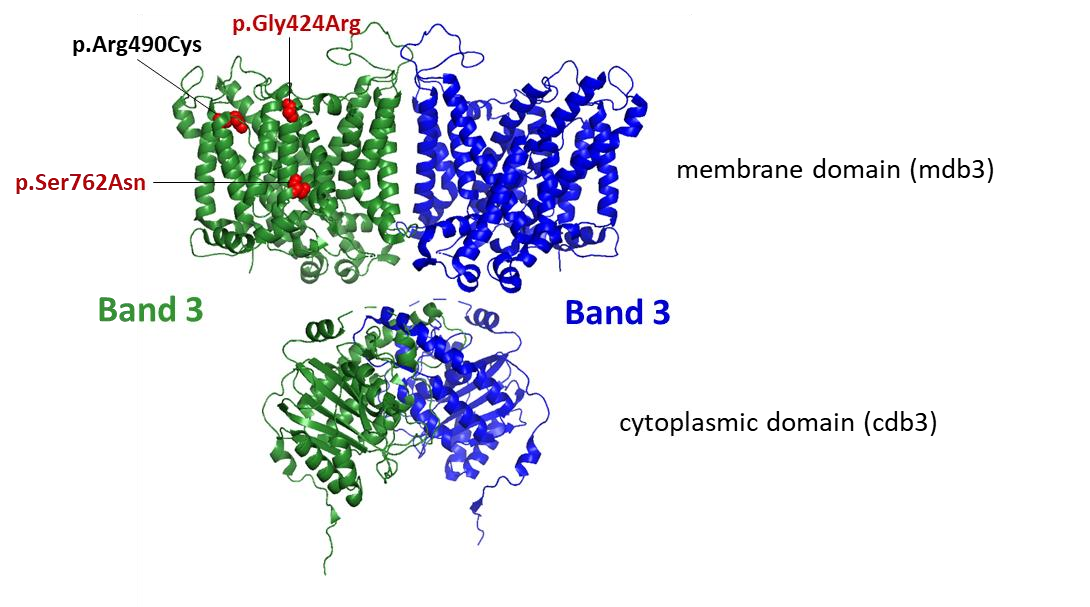


**Figure S12.** A 3D model of band 3 (dimer) with the localization of the herein identified missense variants (red spheres) in SLC4A1 protein (black: already known, red: novel variants), from the Cryo-EM structure of SLC4A1 (PDB-ID: 7TW1) was visualized using PyMol (Schrodinger, LLC. 2010. The PyMOL Molecular Graphics System, Version 2.5.0).

**Figure S13A:** gDNA representation of *SLC4A1*. In red, newly described mutations. In black, mutations previously reported in the literature. The colours of the circles are described in the CADD vs MAF plot. The effect on the protein level is displayed in main figure 1.


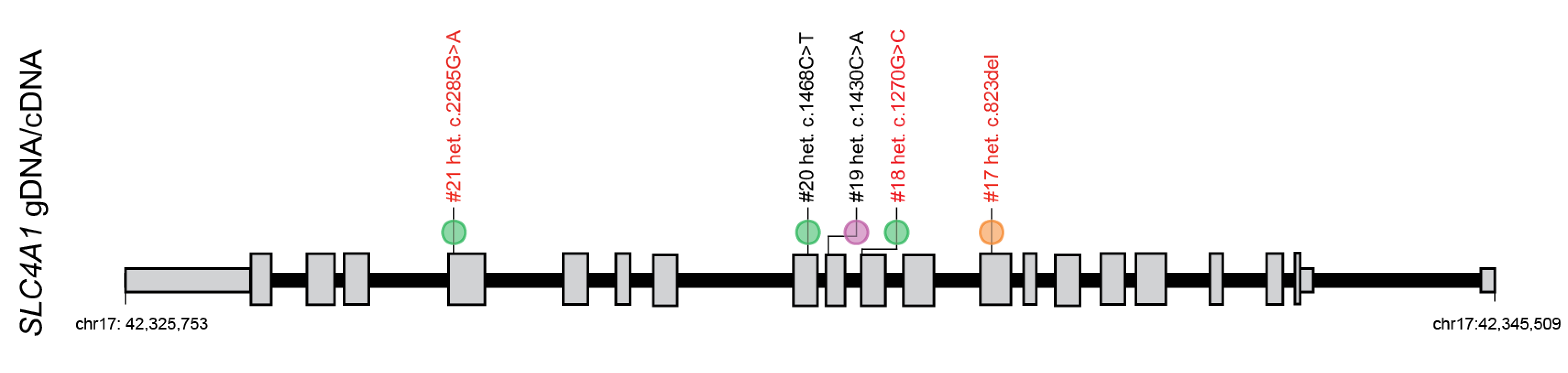


**Figure S13B:** CADD versus minor allele frequency (MAF) plot of all known *SLC4A1* sequence variants visualized by PopViz [Zhang et al. Bioinformatics 2018; 34: 4307–4309]. The horizontal axis shows the MAF scores and the vertical axis the CADD v1.3 ones. The specific types of the various sequence variants, which were collected from the gnomAD r2.0.2 database (https://gnomad.broadinstitute.org/), are colour-coded, and the variants of our patients are square-shaped. CNVs are not included in the CADD/MAF plot.


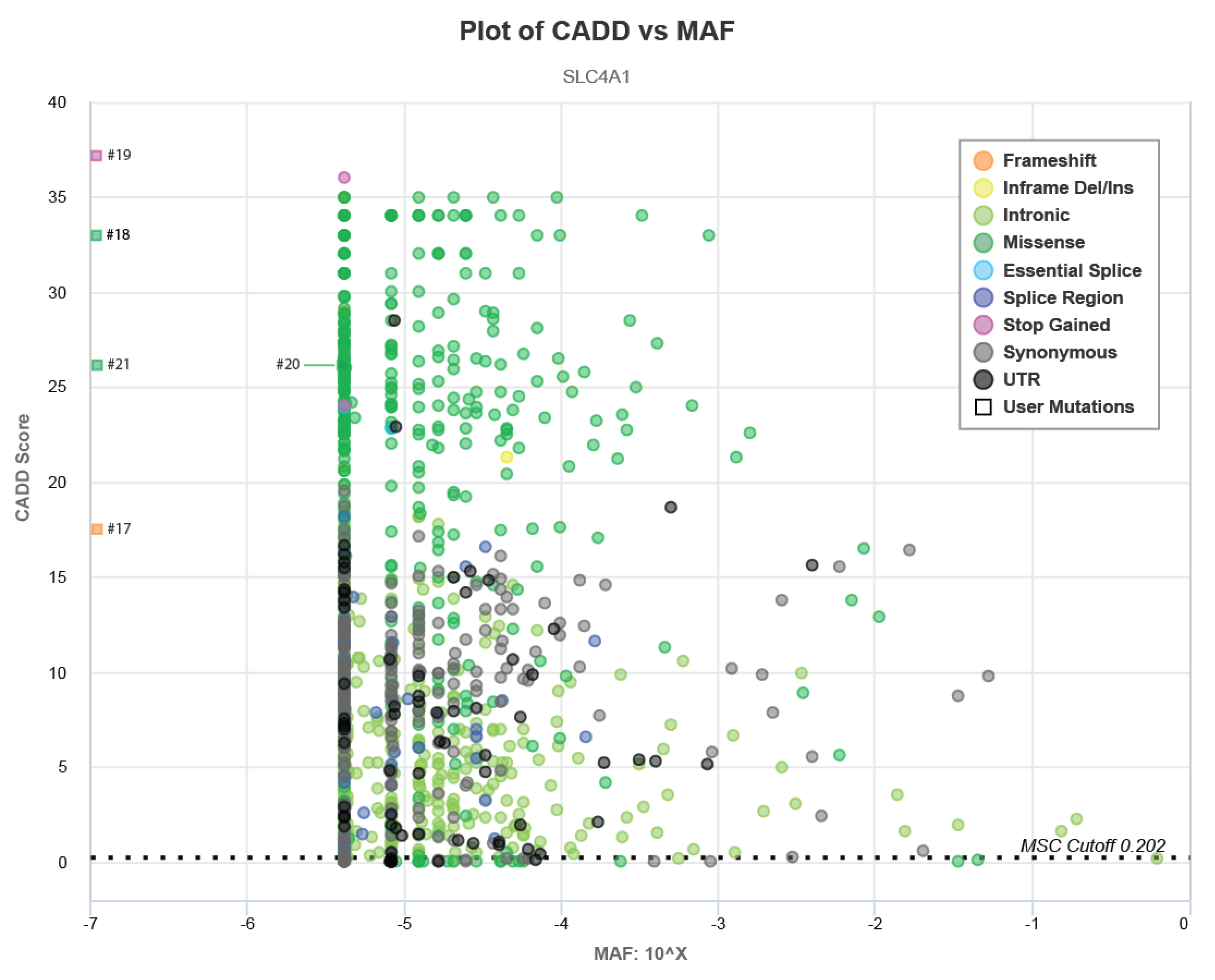


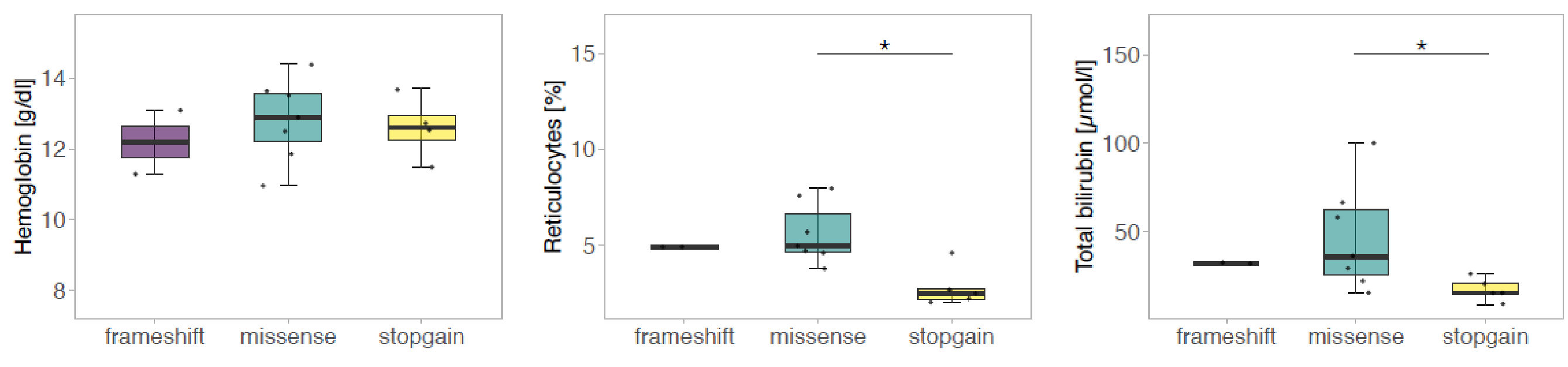


**Figure S14.** HS phenotypes and types of variants in *SLC4A1*.

From the five variants in SLC4A1 identified in our cohort, three are novel, one was reported by us previously and not yet identified by others [Kager L, et al. Pediatr Blood Cancer. 2017 Mar;64(3)], and one was identified in 6 families [Dhermy et al. Br J Haematol. 1997 Jul;98(1):32-40; Wang R, et al. Sci China Life Sci. 2018 Aug;61(8):947-953; Wang et al. Clin Genet. 2023 Jan;103(1):67-78] (Table S5).

**Table S5.** Disease-causing *SLC4A1* variants identified in our study which are described in ≥ 2 families.

| **Family No. in our cohort** | **cDNA change** | **Protein change** | **Consequence** | **No. of patients/families** | **Ancestries** | **PMID** |
| --- | --- | --- | --- | --- | --- | --- |
| 20 | c.1468C>T | p.Arg490Cys | Missense | 10/6 | Central Europe, West Europe, East Asia | 9233560  29572776  36203343 |

Abbreviation: PMID, Pubmed ID

**Index patients (P) and their families (F) with causative variants in *SPTB***

**Family F22 (patients P22, P61): Index group patient P22**, female, 6-years-old, Central European, mild HS phenotype

| **GENE** | **CHR** | **POS** | **IDS** | **REF** | **ALT** | **gnomAD _AF** | **gnomAD _AC** | **gnomAD _hom** | **Consequence** | **HGVSc (cDNA)** | **HGVSp (protein)** | **READS** | **SIFT** | **PolyPhen** | **CADD** |
| --- | --- | --- | --- | --- | --- | --- | --- | --- | --- | --- | --- | --- | --- | --- | --- |
| *SPTB* | 14 | 65271803 | . | G | A | NA | NA | NA | Missense | ENST00000389722.3:  c.154C>T | ENSP00000374372.3:  p.Arg52Trp | 628 | Dele- terious | Probably _damaging | 34 |

| **Method** | **LELY** | **αLEPRA** | **PMID** | **Pathogenicity (split)** |
| --- | --- | --- | --- | --- |
| Panel | Wild type | Wild type | 36035481 | PS4 supporting+PM2+PP1+PP3+PP4 |

Interpretation: The heterozygous *SPTB* c.154C>T missense variant was discovered in patient P22 via our Hematology-Panel. She has mild HS phenotype (Table S1c). Her father (P61) has moderate HS phenotype and developed gallstones (Table S2c) and carries the same pathogenic variant, as confirmed via Sanger sequencing. The healthy mother carries *SPTB* wt alleles. The patient is *LELY* wt.

The *SPTB* variant has a high CADD prediction score (i.e., 34) and affects a conserved R (Arginine at an alpha helix) at the first (CH1) of the two calponin repeats (CH1 and CH2, residues 51–156 and 171–282, respectively) of the actin binding domain within the N-terminal sequence of the βspectrin molecule (Figure 1C) [An et al., Biochemistry 2005, 44, 10681-10688; Lux SE 4th. Blood. 2016 Jan 14;127(2):187-99].

*SPTB* c.154C>T is likely pathogenic (class 4) and the autosomal dominant inherited Mendelian genotype that causes the HS phenotype in patient P22 and her father P61. This variant (rs1594796374) was recently described in an Italian female who was 12-years-old at HS diagnosis and underwent splenectomy at the age of 25 years. The Italian patient had a positive family history of HS, did not receive any transfusions, and biochemically had a 14% decrease in spectrin as analyzed via SDS-PAGE [Vercellati C et al. Front Physiol. 2022 Aug 12;13:949044].

**Family F23 (patients P23, P62): Index group patient P23**, female, 7-years-old, Central European, moderate HS phenotype

| **GENE** | **CHR** | **POS** | **IDS** | **REF** | **ALT** | **gnomAD _AF** | **gnomAD _AC** | **gnomAD _hom** | **Consequence** | **HGVSc (cDNA)** | **HGVSp (protein)** | **READS** | **SIFT** | **PolyPhen** | **CADD** |
| --- | --- | --- | --- | --- | --- | --- | --- | --- | --- | --- | --- | --- | --- | --- | --- |
| *SPTB* | 14 | 65263351 | . | T | TG | NA | NA | NA | Frameshift | ENST00000389722.3:  c.1264dup | ENSP00000374372.3:  p.Gln422ProfsTer7 | 688 | NA | NA | 35 |

| **Method** | **LELY** | **αLEPRA** | **PMID** | **Pathogenicity (split)** |
| --- | --- | --- | --- | --- |
| Panel + WES | Wild type | Wild type | - | PVS1+PM2+PP1+PP3+PP4 |

Interpretation: The heterozygous *SPTB* c.1264dupC frameshift variant was discovered in patient P23 via Hematology-Panel/WES. She has moderate HS phenotype and developed gallstones at the age of 4 years (Table S1c). Her mother (P62) has a similar HS phenotype and also early developed gallstones and underwent cholecystectomy and splenectomy at the age of 11 years (Table S2c) and carries the same pathogenic variant, as confirmed via Sanger sequencing. The healthy father carries *SPTB* wt alleles. The patient is *LELY* wt.

The *SPTB* variant has a high CADD prediction score (i.e., 35) and leads to a frameshift causing a premature stop codon. The variant per se affects a Q (Glutamine in an alpha helix) at the begin of the second of the 17 β-spectrin repeats (Figure 1C) at the N-terminal dimerization domain [Lux SE 4th. Blood. 2016 Jan 14;127(2):187-99]. Spectrin dimerization domain encompass spectrin β-repeats β1 (aa 303-411) and β2 (aa 423-525) [https://www.uniprot.org/uniprotkb/P11277/entry].

*SPTB* c.1264dup is pathogenic (class 5) and the autosomal dominant inherited Mendelian genotype that causes the HS phenotype in patient P23 and her mother P62. The variant is novel.

**Family F24: Index group patient P24**, male, 16-years-old, Central European, moderate HS phenotype

| **GENE** | **CHR** | **POS** | **IDS** | **REF** | **ALT** | **gnomAD _AF** | **gnomAD _AC** | **gnomAD _hom** | **Consequence** | **HGVSc (cDNA)** | **HGVSp (protein)** | **READS** | **SIFT** | **PolyPhen** | **CADD** |
| --- | --- | --- | --- | --- | --- | --- | --- | --- | --- | --- | --- | --- | --- | --- | --- |
| *SPTA1* | 1 | 158592867 | . | C | T | 0,00018 | 52 | 0 | Missense | ENST00000368147.4:  c.6026G>A | ENSP00000357129.4:  p.Arg2009His | 202 | Dele- terious | Possibly  _damaging | 26.5 |
| *SPTB* | 14 | 65260587 | . | T | C | NA | NA | NA | Splice-accept | ENST00000389722.3:  c.1796-2A>G | - | 25 | NA | NA | 23.3 |

| **Gene** | **Method** | **LELY** | **αLEPRA** | **PMID** | **Pathogenicity (split)** |
| --- | --- | --- | --- | --- | --- |
| *SPTA1* | WES | Wild type | Wild type | - | PM2+PP3+PP4 |
| *SPTB* | WES | Wild type | Wild type | - | PVS1+PM2+PM6+PP3+PP4 |

Interpretation: The heterozygous *SPTB* c.1796-2A>G splicing variant was discovered in patient P24 via WES. He has moderate HS phenotype (Table S1c). Neither his healthy parents, nor his healthy brother (laboratory data not shown) carry the variant (Figure S15). The patient is *LELY* wt.

The *SPTB* variant has a moderate CADD prediction score (i.e., 23.3) and affects a highly conserved intronic base. The variant is predicted *in silico* to be an acceptor splice-site variant.

The *SPTB* c.1796-2A>G splicing variant is pathogenic (class 5) and the Mendelian genotype that causes the HS phenotype in patient P24. The variant per se is novel. However, two different variants, which affect the same highly conserved splice-site have been identified. A *de novo*, likely pathogenic variant at the neighbouring position (i.e., c.1796-1G>C) was reported by Tole et al. in one patient in a Canadian study [Tole S, et al. Br J Haematol.

2020 Nov;191(3):486-496]. In addition, Park et al. described a *de novo* heterozygous splice variant, that is *SPTB* c.1795 + 2_1795 + 3delTG in a 17years-old male with HS [Park et al. Clin Genet. 2016 Jul;90(1):69-78]. It is of interest, that the above described three variants (i.e., *SPTB* c.1796-2A>G, c.1796-1G>C, and c.1795 + 2_1795 + 3delTG) which affect a highly conserved splice site, occurred *de novo* in all three yet identified patients.

In addition, a heterozygous *SPTA1* c.6026G>A missense variant was identified in patient P24. This variant also has a moderate CADD score (i.e., 26.5) and may modify the phenotype. Unfortunately, we were unable to test the *SPTA1* variant in the family members. We therefore classify this variant as variant of unknown significance (class 3, VUS).


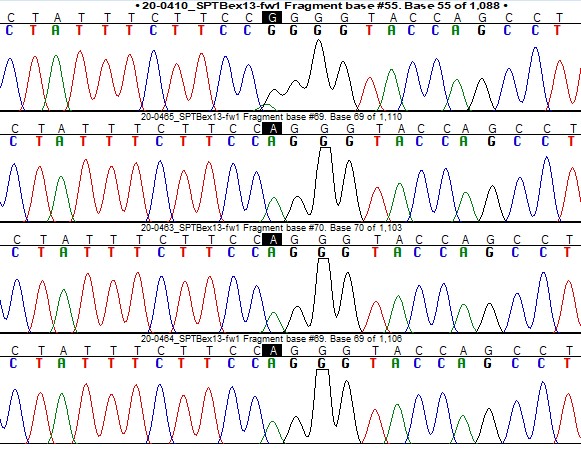


**Figure S15:** *SPTB* splice acceptor variant segregation. Starting from top to bottom of the chromatograms: Index patient (HET), sister (WT), mother (WT) and father (WT).

**Family F25: Index group patient P25**, female, 10-years-old, Central European, moderate HS phenotype

| **GENE** | **CHR** | **POS** | **IDS** | **REF** | **ALT** | **gnomAD _AF** | **gnomAD _AC** | **gnomAD _hom** | **Consequence** | **HGVSc (cDNA)** | **HGVSp (protein)** | **READS** | **SIFT** | **PolyPhen** | **CADD** |
| --- | --- | --- | --- | --- | --- | --- | --- | --- | --- | --- | --- | --- | --- | --- | --- |
| *SPTB* | 14 | 65260469 | . | G | A | NA | NA | NA | Stop gain | ENST00000389722.3:  c.1912C>T | ENSP00000374372.3:  p.Arg638Ter | 713 | NA | NA | 36 |

| **Method** | **LELY** | **αLEPRA** | **PMID** | **Pathogenicity (split)** |
| --- | --- | --- | --- | --- |
| Panel | Wild type | Wild type | 31723846  31602632  31980736  34335240 | PVS1+ PS4moderate+ PM2+PP3+PP4 |

Interpretation: The heterozygous *SPTB* c.1912C>T stop-gain variant was discovered in patient P25 via our Hematology-Panel. She has moderate HS phenotype and developed gallstones at the age of 6 years (Table S1c). Her mother and her father have no HS phenotypes (laboratory data not shown); the mother agreed to genetic testing and is *SPTB* wt; the father refused genetic analyses; therefore, the mode of inheritance was classified as ‘unknown’. The patient is *LELY* wt.

The *SPTB* variant has a high CADD prediction score (i.e., 36) and affects an R (Arginine within an alpha helix) in the fourth (β4, aa 638-741) of the 17 β-spectrin repeats (Figure 1C).

*SPTB* c.1912C>T stop-gain variant is pathogenic (class 5) and the inherited Mendelian genotype that causes the HS phenotype in patient P25. In a recent review based on 13 selected studies with N=627 patients, the *SPTB* c.1912C>T stop-gain variant was reported to be a ‘high frequency’ variant (frequency, N=5) [Yang L, et al. Clin Genet. 2022 Sep 7. doi: 10.1111/cge.14223]. For example, it was identified in a study performed in the Netherlands in one patient with moderate HS phenotype [van Vuren et al., Hemasphere. 2019 Aug 7;3(4):e276], in a 1-months-old male and a 20years-old female with HS in studies performed in China [Qin et al. J Hum Genet. 2020 Apr;65(4):427-434, Wang D et al. Front Pharmacol. 2021 Jul 16;12:644352.], and in one patient in an investigation from India [Aggarwal et al., Br J Haematol. 2020 Mar;188(5):784-795].

**Family F26: Index group patient P26**, female, 19-years-old, Central European, moderate HS phenotype

| **GENE** | **CHR** | **POS** | **IDS** | **REF** | **ALT** | **gnomAD _AF** | **gnomAD _AC** | **gnomAD _hom** | **Consequence** | **HGVSc (cDNA)** | **HGVSp (protein)** | **READS** | **SIFT** | **PolyPhen** | **CADD** |
| --- | --- | --- | --- | --- | --- | --- | --- | --- | --- | --- | --- | --- | --- | --- | --- |
| *SPTB* | 14 | 65260425 | . | C | T | NA | NA | NA | Stop gain | ENST00000389722.3:  c.1956G>A | ENSP00000374372.3:  p.Trp652Ter | 239 | NA | NA | 39 |

| **Method** | **LELY** | **αLEPRA** | **PMID** | **Pathogenicity (split)** |
| --- | --- | --- | --- | --- |
| WES | Heterozygous | Wild type | 29505016 | PVS1+PS4 supporting+PM2+PP3+PP4 |

Interpretation: The heterozygous *SPTB* c.1956G>A stop-gain variant was discovered in patient P26 via WES. She has moderate HS phenotype and developed gallstones for which she underwent cholecystectomy at age 17-years (Table S1c). No parents were available for testing (mother deceased, father refused analyses). The patient carries a heterozygous *LELY* variant.

The *SPTB* variant has a very high CADD prediction score (i.e., 39). The variant per se affects a highly conserved W (Tryptophan in an alpha helix) fourth (β4, aa 638-741) of the 17 β-spectrin repeats (Figure 1C).

*SPTB* c.1956G>A is pathogenic (class 5) and the Mendelian genotype that causes the HS phenotype in patient P26. The *SPTB* p.Trp652Ter variant was first reported by Shin et al. in a 65-year-old female Korean. The Korean patient underwent cholecystectomy about the age of 45-years and splenectomy for anemia with Hb 6.6g/dL at the age of 65 years. After splenectomy, Hb levels returned to normal. Although one son (underwent cholecystectomy) and two of her son’s children (both had neonatal jaundice) were reported to have had a clinical diagnosis of HS, no genetic testing was performed in the three family members [Shin et al., Medicine (Baltimore). 2018 Jan;97(3):e9677].

**Family F27 (patients P27, P63): Index group patient P27**, female, 11-years-old, Central European, moderate HS phenotype

| **GENE** | **CHR** | **POS** | **IDS** | **REF** | **ALT** | **gnomAD _AF** | **gnomAD _AC** | **gnomAD _hom** | **Consequence** | **HGVSc (cDNA)** | **HGVSp (protein)** | **READS** | **SIFT** | **PolyPhen** | **CADD** |
| --- | --- | --- | --- | --- | --- | --- | --- | --- | --- | --- | --- | --- | --- | --- | --- |
| *SPTB* | 14 | 65246606 | . | T | TC | NA | NA | NA | Frameshift | ENST00000389722.3:  c.4309dup | ENSP00000374372.3: p.Glu1437GlyfsTer54 | 732 | NA | NA | 35 |

| **Method** | **LELY** | **αLEPRA** | **PMID** | **Pathogenicity (split)** |
| --- | --- | --- | --- | --- |
| Panel | Heterozygous | Wild type | 29797310 | PVS1+PM2+PM4+PP1+PP3+PP4 |

Interpretation: The heterozygous *SPTB* c.4309dup frameshift variant was discovered in patient P27 via our Hematology-Panel and was previously published by us [Kager et al., Br J Haematol. 2018 Jul;182(2):251-258]. She has moderate HS phenotype, developed gallstones at age 6 years and underwent cholecystectomy and subtotal splenectomy at age 7 years (Table S1c). Her father (P63) has also moderate HS phenotype (Table S2c) and carries the same pathogenic variant, as confirmed via Sanger sequencing. Her healthy mother and brother both carry *SPTB* wt alleles. The patient carries a heterozygote *LELY* variant.

The *SPTB* variant has a high CADD prediction score (i.e., 35) and causes a frameshift which leads to a premature stop codon, leading to an erroneous truncated protein. The variant per se affects an E (Glutamic acid within a coil sequence) in the 11^th^ (β11, aa 1389-1465) of the 17 β-spectrin repeats (Figure 1C).

*SPTB* c.4309dup is pathogenic and the autosomal dominant Mendelian genotype that causes the HS phenotype in patient P27 and her father P63. The variant was published by us in 2018 [Kager et al., Br J Haematol. 2018 Jul;182(2):251-258] and was not yet reported by others.

**Family F28 (patients P28, P64): Index group patient P28**, female, 2-years-old, Central European, moderate HS phenotype

| **GENE** | **CHR** | **POS** | **IDS** | **REF** | **ALT** | **gnomAD _AF** | **gnomAD _AC** | **gnomAD _hom** | **Consequence** | **HGVSc (cDNA)** | **HGVSp (protein)** | **READS** | **SIFT** | **PolyPhen** | **CADD** |
| --- | --- | --- | --- | --- | --- | --- | --- | --- | --- | --- | --- | --- | --- | --- | --- |
| *SPTB* | 14 | 65240046 | . | G | T | NA | NA | NA | Stop gain | ENST00000389722.3:  c.5070C>A | ENSP00000374372.3:  p.Tyr1690Ter | 110 | NA | NA | 39 |
| *SPTB* | 14 | 65239294 | rs372593234 | G | A | 0,000097 | 30 | 0 | Splice region intron | ENST00000389722.3:  c.5553+4C>T |  | 47 | NA | NA | 10.97 |

| **Gene** | **Method** | **LELY** | **αLEPRA** | **PMID** | **Pathogenicity (split)** |
| --- | --- | --- | --- | --- | --- |
| *SPTB* (c.5070C>A) | WES | Heterozygous | Wild type | - | PVS1+PM2+PP1+PP3+PP4 |
| *SPTB* (c.5553+4C>T) | WES | Heterozygous | Wild type | - | PP1+PP4+BS1+BP4 |

Interpretation: The heterozygous *SPTB* c.5070C>A stop-gain variant was discovered in P28 via WES. She has a moderate HS phenotype (Table S1c). Her father (P64) had a severe HS phenotype before splenectomy (diagnosis of gall stones at age 15 years, cholecystectomy and splenectomy at age 18 years; Table S2c) and carries the same pathogenic variant, as confirmed via Sanger sequencing. Her healthy mother carries *SPTB* wt alleles. The patient carries a heterozygote *LELY* variant.

The *SPTB* variant has a very high CADD prediction score (i.e., 39) and causes a frameshift which leads to a premature stop codon, leading to an erroneous truncated protein. It affects a Y (Tyrosine in a coil sequence) in the 14^th^ (β14, aa 1690-1793) of the 17 β-spectrin repeats (Figure 1C). β14 and β15 together form the ankyrin binding domain [Ipsaro et al. Blood. 2009 May 28;113(22):5385-93].

*SPTB* c.5070C>A is pathogenic (class 5) and the autosomal dominant Mendelian genotype that causes the HS phenotype in P28 and her father P64. The variant is novel.

P28 and her father (P64) both carry one additional likely benign (class 2) variant in *SPTB*; that is c.5553+4C>T. This splice region intronic variant has a low CADD score (i.e., 10.97) and 30 heterozygotes are listed in gnomAD. Moreover, in silico investigations did not predict any splicing effect of this variant. Whereas we do not consider this variant to be the cause of the moderate and severe phenotypes of patient P28 and P64, we cannot rule out, that the variant has a modifier activity. The mother is wt for both *SPTB* variants (Figure S16).

In addition, P28 carries homozygous *UGT1A1**28 alleles (rs3064744) which cause Gilbert-Meulengracht syndrome, and this may have contributed to the rather high bilirubin levels (i.e., median 91.8µmol/L).


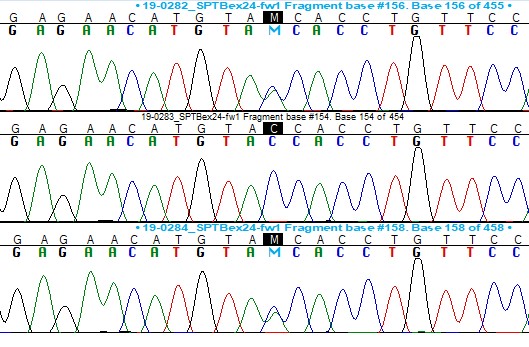

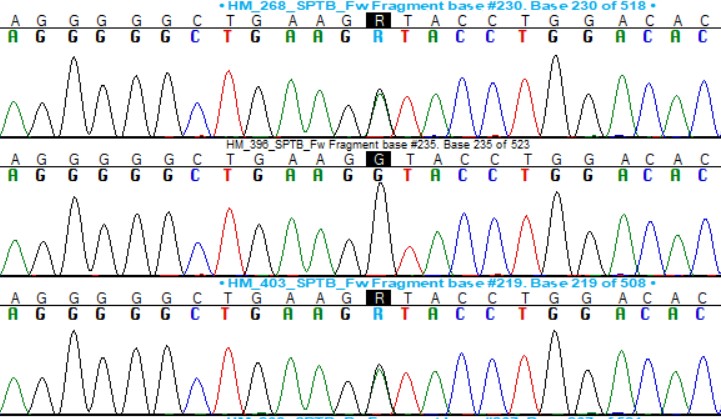


**Figure S16:** *SPTB* nonsense variant segregation (left) and *SPTB* splice variant segregation (right). Starting from top to bottom of the chromatograms for both variants, nonsense and splicing, respectively: Index patient (HET, HET), mother (WT, WT) and father (HET, HET).

**Family F29 (patients P29, P65, P66): Index group patient P29,** female, 10-years-old, Central European, mild HS phenotype

| **GENE** | **CHR** | **POS** | **IDS** | **REF** | **ALT** | **gnomAD _AF** | **gnomAD _AC** | **gnomAD _hom** | **Consequence** | **HGVSc (cDNA)** | **HGVSp (protein)** | **READS** | **SIFT** | **PolyPhen** | **CADD** |
| --- | --- | --- | --- | --- | --- | --- | --- | --- | --- | --- | --- | --- | --- | --- | --- |
| *SPTB* | 14 | 65240011 | . | A | G | NA | NA | NA | Missense | ENST00000389722.3:  c.5105T>C | ENSP00000374372.3:  p.Leu1702Pro | 323 | deleterious | Probably _damaging | 25.8 |

| **Method** | **LELY** | **αLEPRA** | **PMID** | **Pathogenicity (split)** |
| --- | --- | --- | --- | --- |
| Panel | Wild type | Wild type | - | PM1+PM2+PP1+PP3+PP4 |

Interpretation: The heterozygous *SPTB* c.5105T>C missense variant was discovered in P29 via our Hematology-Panel. She has a mild HS phenotype (Table S1c). Her father (P65) and her brother (P66) also have very mild HS phenotypes (Table S2c) and both carry the same variant, as confirmed via Sanger sequencing. Her healthy mother carries *SPTB* wt alleles. The patient is *LELY* wt.

The *SPTB* variant has a moderate CADD prediction score (i.e., 25.8). It affects a highly conserved L (Leucine in an alpha helix) in the 14^th^ (β14, aa

1690-1793) of the 17 β-spectrin repeats (Figure 1C). β14 and β15 together form the ankyrin binding domain [Ipsaro et al. Blood. 2009 May 28;113(22):5385-93].

*SPTB* p.Leu1702Pro is likely pathogenic (class 4) and the autosomal dominant Mendelian genotype that causes the mild HS phenotype in P29, her brother P66 and her father P65. The variant is novel.

**Family F30 (patients P30, P67): Index group patient P30**, male, 13-years-old, Central European, mild HS phenotype

| **GENE** | **CHR** | **POS** | **IDS** | **REF** | **ALT** | **gnomAD _AF** | **gnomAD _AC** | **gnomAD _hom** | **Consequence** | **HGVSc (cDNA)** | **HGVSp (protein)** | **READS** | **SIFT** | **PolyPhen** | **CADD** |
| --- | --- | --- | --- | --- | --- | --- | --- | --- | --- | --- | --- | --- | --- | --- | --- |
| *SPTB* | 14 | 65239949 | . | CAA | C | NA | NA | NA | Frameshift | ENST00000389722.3:  c.5165_5166del | ENSP00000374372.3:  p.Phe1722Ter | 192 | NA | NA | 35 |

| **Method** | **LELY** | **αLEPRA** | **PMID** | **Pathogenicity (split)** |
| --- | --- | --- | --- | --- |
| Panel | Heterozygous | Wild type | 33761640 | PVS1+ PS4_supporting+ PM2+PP1+PP3+PP4 |

Interpretation: The heterozygous *SPTB* c.5165_5166del frameshift variant was discovered in P30 via our Hematology-Panel. He has a mild HS phenotype (Table S1c). His father (P67) who has a moderate HS phenotype (cholecystectomy for gall stones at age 24 years [Table S2c]) carries the same pathogenic variant, as confirmed via Sanger sequencing. His healthy mother carries *SPTB* wt alleles. The patient carries a heterozygous *LELY* variant.

The *SPTB* variant causes a frameshift, resulting in an early premature stop codon, leading to an erroneous truncated protein. Moreover, there is a high CADD prediction score (i.e., 35) for the identified variant. The variant per se affects an F (Phenylalanine in a coil sequence) in the 14^th^ (β14, aa 1690-

1793) of the 17 β-spectrin repeats (Figure 1C). β14 and β15 together form the ankyrin binding domain [Ipsaro et al. Blood. 2009 May 28;113(22):538593].

*SPTB* c.5165_5166del is pathogenic (class 5) and the autosomal dominant Mendelian genotype that causes the HS phenotype in P30 and his father P67. The *SPTB* c.5165_5166del variant was recently identified in a Chinese male infant, suffering from hydrops fetalis in week 29 of gestation. After caesarian section at week 33, the newborn received exchange transfusion because of very high bilirubin levels and Coombs negative hemolytic anemia; at the age of 42 days another RBC transfusion was given for Hb 6.4 g/dL. Thereafter, no further transfusions were necessary, and the hemoglobin was 8.5 g/dL to 10.5 g/dL. No EMA testing was performed in the Chinese patient, and his parents were reported to have *SPTB* wild types, and no HS phenotypes [Zhang et al., 2021 Mar 26;100(12):e24804].

**Family F31: Index group patient P31**, male, 18-years-old, Central European, severe HS phenotype

| **GENE** | **CHR** | **POS** | **IDS** | **REF** | **ALT** | **gnomAD _AF** | **gnomAD _AC** | **gnomAD _hom** | **Consequence** | **HGVSc (cDNA)** | **HGVSp (protein)** | **READS** | **SIFT** | **PolyPhen** | **CADD** |
| --- | --- | --- | --- | --- | --- | --- | --- | --- | --- | --- | --- | --- | --- | --- | --- |
| *SPTB* | 14 | 65235811 | . | CAT | GA | NA | NA | NA | Frameshift | ENST00000389722:  c.5961_5964delinsTTC | ENSP00000374372.3:  p.Met1988SerfsTer7 | 255 | NA | NA | 27.5 |

| **Method** | **LELY** | **αLEPRA** | **PMID** | **Pathogenicity (split)** |
| --- | --- | --- | --- | --- |
| Panel | Wild type | Wild type | 29797310 | PVS1+PM2+PP3+PP4 |

Interpretation: The heterozygous c.5961_5964delinsTTC frameshift variant was discovered in P31 via our Hematology-Panel and was previously published by us [Kager et al., Br J Haematol. 2018 Jul;182(2):251-258]. He has a severe HS phenotype and underwent cholecystectomy and splenectomy at age 16 years (Table S1c). His healthy mother (laboratory data not shown) carries *SPTB* wt alleles. His father was deceased; but was reported to have had no HS phenotype. The patient carries *LELY* wt.

The *SPTB* variant causes a frameshift, resulting in an early premature stop codon, leading to an erroneous truncated protein. There is a moderate CADD prediction score (i.e., 27.5) for the identified variant. The variant per se affects an M (Methionine in an alpha helix region) in the 16^th^ (β16, aa 19062006) of the 17 β-spectrin repeats (Figure 1C).

*SPTB* c.5961_5964delinsTTC is pathogenic (class 5) and the Mendelian genotype that causes the HS phenotype in P31. The variant was first reported by us and was not identified yet by others.

**Family F32 (patients P32, P68): Index group patient P32**, female, 23-years-old, Central European, severe HS phenotype

| **GENE** | **CHR** | **POS** | **IDS** | **REF** | **ALT** | **gnomAD _AF** | **gnomAD _AC** | **gnomAD _hom** | **Consequence** | **HGVSc (cDNA)** | **HGVSp (protein)** | **READS** | **SIFT** | **PolyPhen** | **CADD** |
| --- | --- | --- | --- | --- | --- | --- | --- | --- | --- | --- | --- | --- | --- | --- | --- |
| *SPTB* | 14 | 65235793 | . | ATC | A | NA | NA | NA | Frameshift | ENST00000389722.3:  c.5979_5980del | ENSP00000374372.3:  p.Glu1993AspfsTer3 | 247 | NA | NA | 36 |

| **Method** | **LELY** | **αLEPRA** | **PMID** | **Pathogenicity (split)** |
| --- | --- | --- | --- | --- |
| Panel | Wild type | Wild type | - | PVS1+PM2+PP1+PP3+PP4 |

Interpretation: The heterozygous c.5979_5980del frameshift variant was discovered in P32 via our Hematology-Panel. She has a moderate HS phenotype and underwent cholecystectomy at age 13 years (Table S1c). Her father (P68) has a similar HS phenotype (underwent cholecystectomy and splenectomy at age 8 years; Table S2c) and carries the same pathogenic *SPTB* variant, as confirmed via Sanger sequencing. Her healthy mother carries *SPTB* wt alleles. P32 carries *LELY* wt.

The *SPTB* variant causes a frameshift, resulting in an early premature stop codon, leading to an erroneous truncated protein. There is a high CADD prediction score (i.e., 36) for the identified variant. The variant per se affects an E (Glutamic acid in an alpha helix region) in the 16^th^ (β16, aa 19062006) of the 17 β-spectrin repeats (Figure 1C).

The *SPTB* c.5979_5980del is pathogenic (class 5) and the Mendelian genotype that causes the HS phenotype in P32 and her father P68. The variant is novel.

**Summary *SPTB v*ariants**

73% of index patients with causative *SPTB* variants had high impact alterations – that is, stop gain and frameshift variants (Figure S17). Patients with missense variants (N=2) had higher hemoglobin values compared to patients with stop-gain variants. There was a trend towards milder phenotypes in patients with *SPTB* missense variants, compared to patients with high-impact variants (Figure S19).


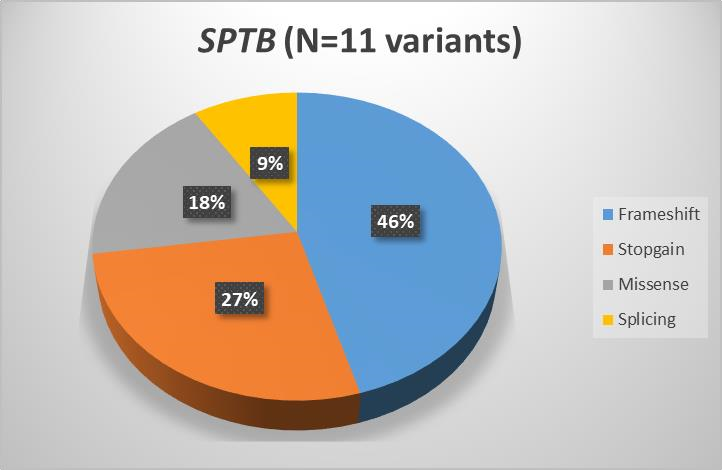


**Figure S17:** The 11 causative variants and the types of variation in *SPTB* identified in index group patients. Frameshift, N=5 (patients P23, P27, P30, P31, P32); stop-gain, N=3 (P25, P26, P28); missense variants, N=2 (P22, P29); Splicing, N=1 (P24).

According to the annotation of pathogenicity based on the Standard Guidelines for the Interpretation of Sequence Variants [(https://pubmed.ncbi.nlm.nih.gov/25741868/)](https://pubmed.ncbi.nlm.nih.gov/25741868/), both the missense variants (P22, P29) were classified as likely pathogenic, all other variants were classified as pathogenic. A 3D-model of the location of the two missense variants in *SPTB* is provided in Figure S18.


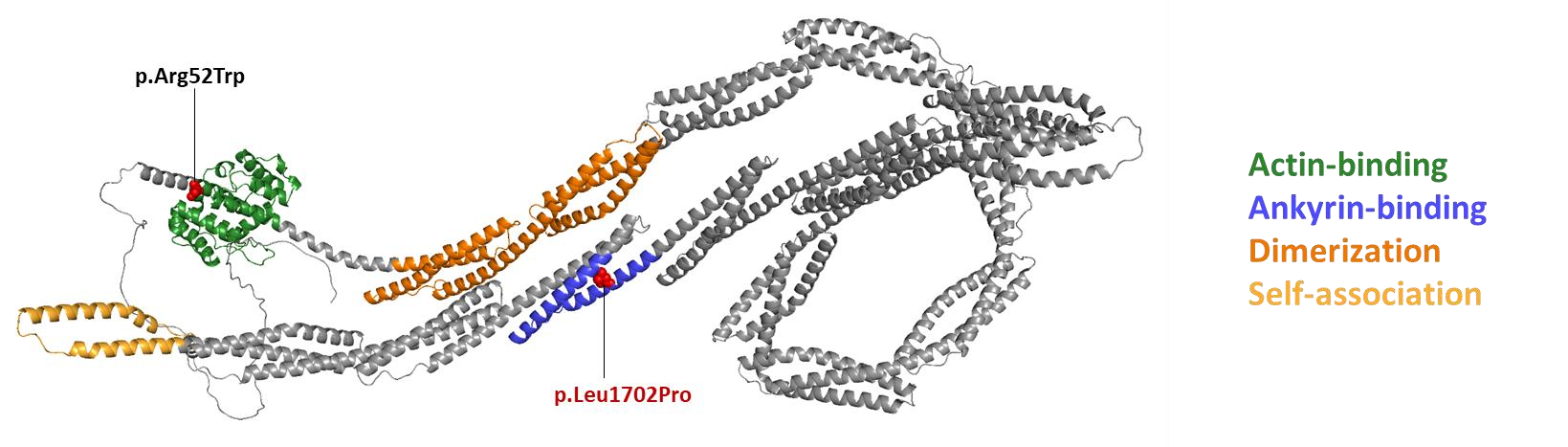


**Figure S18**: A 3D model of an SPTB Alphafold model (AF-P11277-F1) was visualized using PyMol (Schrodinger, LLC. 2010. The PyMOL Molecular Graphics System, Version 2.5.0), with the localization (red spheres) of the herein identified missense variants (black: already known, red: novel variant).


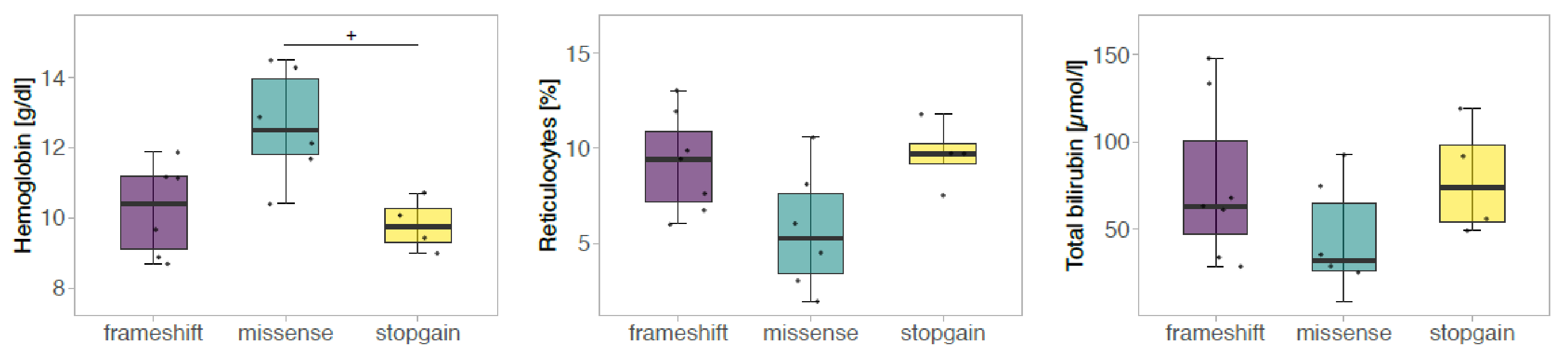


**Figure S19.** HS phenotypes and types of variants in *SPTB*. Of the 11 identified *SPTB* variants, five were novel, two were reported by us earlier and not yet described by others and four were described in ≥ 2 families (Table S6).

**Figure S20A:** gDNA representation of *SPTB*. In red, newly described variants. In black, variants previously reported in the literature. The colours of the circles are described in the CADD vs MAF plot. The effect on the protein level is displayed in main figure 1.


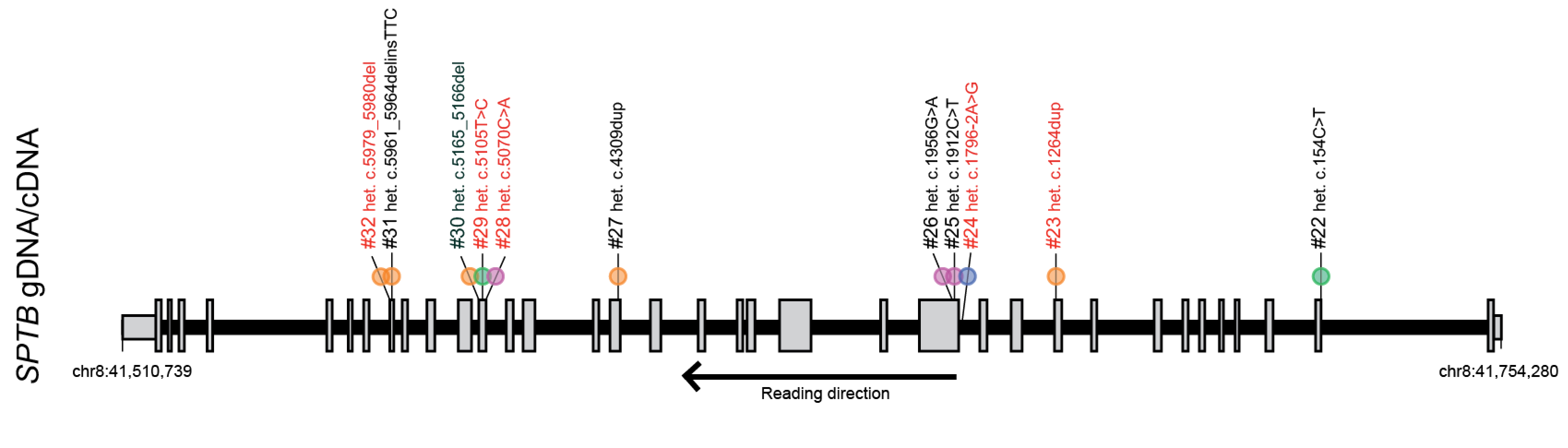


**Figure S20B:** CADD versus minor allele frequency (MAF) plot of all known *SPTB* sequence variants visualized by PopViz [Zhang et al. Bioinformatics 2018; 34: 4307–4309]. The horizontal axis shows the MAF scores and the vertical axis the CADD v1.3 ones. The specific types of the various sequence variants, which were collected from the gnomAD r2.0.2 database (https://gnomad.broadinstitute.org/), are colour-coded, and the variants of our patients are square-shaped. CNVs are not included in the CADD/MAF plot.


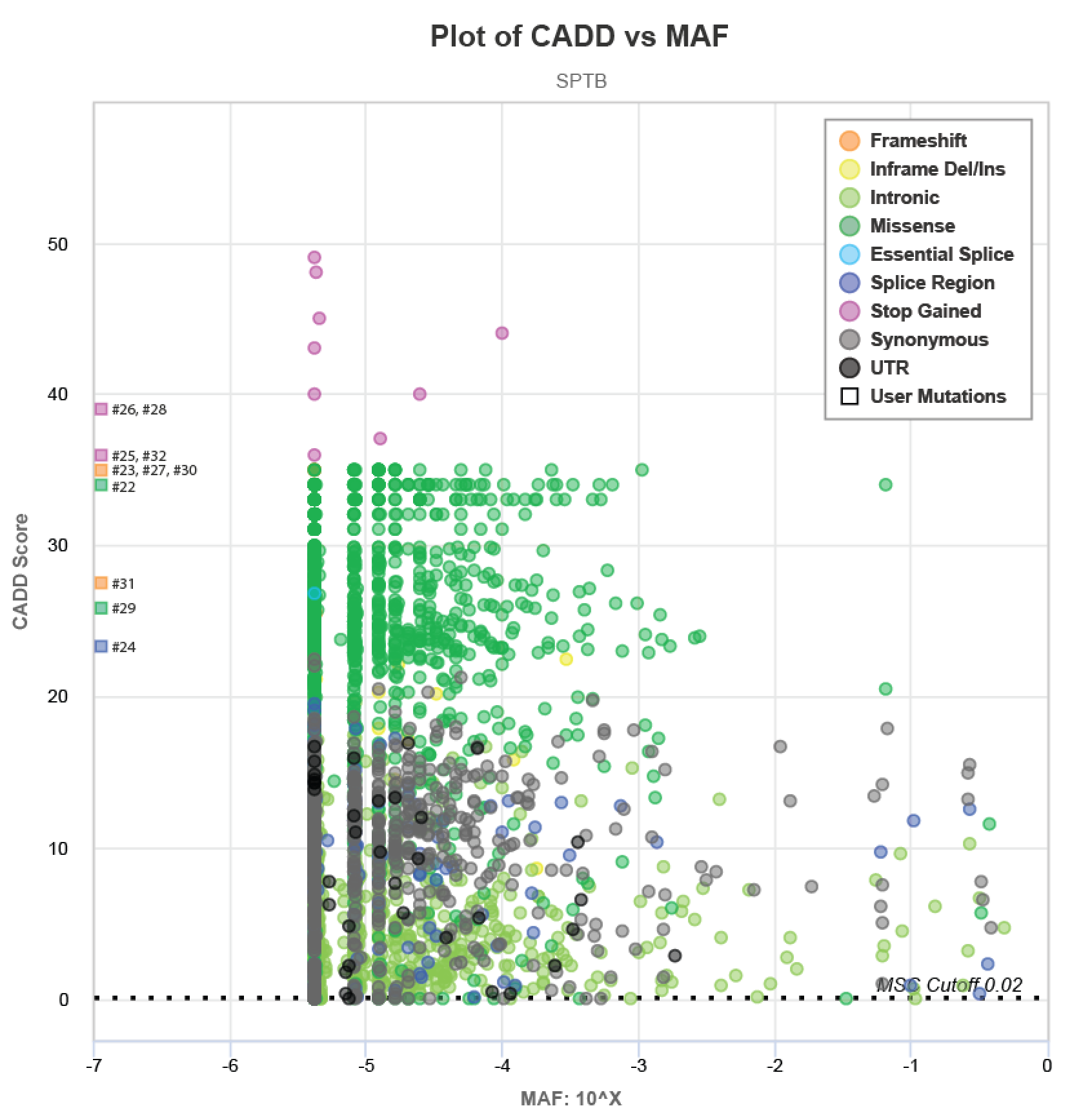


**Table S6.** Disease-causing *SPTB* variants identified in our study which are described in ≥ 2 families.

| **Family No. in our cohort** | **cDNA change** | **Protein change** | **Consequence** | **No. of patients/families** | **Ancestries** | **PMID** |
| --- | --- | --- | --- | --- | --- | --- |
| 22 | c.154C>T | p.Arg52Trp | Missense | 3/2 | Central Europe, South Europe | 36035481 |
| 25 | c.1912C>T | p.Arg638Ter | Stop gain | 5/5 | Central Europe, West Europe, South Asia,  East Asia | 31723846  31602632  31980736  34335240 |
| 26 | c.1956G>A | p.Trp652Ter | Stop gain | 2/2 | Central Europe, South Asia | 29505016 |
| 30 | c.5165_5166del | p.Phe1722Ter | Frameshift | 3/2 | Central Europe, East Asia | 33761640 |

Abbreviation: PMID, Pubmed ID

**Index patients (P) and their families (F) with causative variants in *SPTA1***

**Family F33 (patients P33, P69): Index group patient P33**, female, 17-years-old, Central European, moderate HS phenotype

| **GENE** | **CHR** | **POS** | **IDS** | **REF** | **ALT** | **gnomAD _AF** | **gnomAD _AC** | **gnomAD _hom** | **Consequence** | **HGVSc (cDNA)** | **HGVSp (protein)** | **READS** | **SIFT** | **PolyPhen** | **CADD** |
| --- | --- | --- | --- | --- | --- | --- | --- | --- | --- | --- | --- | --- | --- | --- | --- |
| *SPTA1* | 1 | 158624457 | . | G | A | 0,000004009 | 1 | 0 | Stop gain | ENST00000368147.4:  c.2980C>T | ENSP00000357129.4:  p.Arg994Ter | 46 | NA | NA | 38 |
| *SPTA1* | 1 | 158613314 | . | G | A | 0.004937 | 691 | 3 | Intron | ENST00000368147.4:  c.4339-99C>T | ENSP00000357129.4:  p.Lys1449ValfsTer27 | 46 | NA | NA | 8.7 |
| *ANK1* | 8 | 41551505 | . | C | T | 0,00004474 | 14 | 0 | Missense | ENST00000289734.7:  c.3443G>A | ENSP00000289734.7:  p.Arg1148Gln | 101 | NA | Possibly  _damaging | 29.9 |

| **Gene** | **Method** | **LELY** | **αLEPRA** | **PMID** | **Pathogenicity (split)** |
| --- | --- | --- | --- | --- | --- |
| *SPTA1* | Panel | Wild type | Heterozygous | - | PVS1+PP1+PP3+PP4 |
| *SPTA1* | Panel |  |  | 8941647 | PS1+PS3+PS4+PM3+PP1+PP3+PP4 |
| *ANK1* | Panel |  |  | - | PP1+PP2+PP3+PP4 |

Interpretation: P33 has a moderate HS phenotype. She developed gallstones at age 5 years and underwent cholecystectomy and splenectomy at age 8 years (Table S1d). She has *LELY* wt. Her younger sister (P69) also has a moderate but slightly milder HS phenotype and developed gall stones at age 11 years (Table S2d). Both sisters carry a heterozygous novel pathogenic paternally inherited stop gain variant in *SPTA1* (i.e., NM_003126.2:c.2980C>T) and two maternally inherited sequence variants in HS candidate genes, that is a heterozygous intronic pathogenic low expression variant in *SPTA1* (c.4339-99C>T; called α-LEPRA) and a heterozygous missense variant of unknown significance (VUS) in *ANK1* (c.3443G>A) (Figure S21). The parents do not have HS phenotypes (laboratory data not shown).The pathogenic (class 5) *SPTA1* c.2980C>T variant has a very high CADD score (i.e., 38) and is very rare; only one heterozygote is listed in gnomAD. The *SPTA1* variant is localized in the Src Homology 3 (SH3) domain of the human erythroid α-spectrin [Yang et al. Clin Genet. 2022 Sep 7. doi: 10.1111/cge.14223]. The exact function of the SH3 domain, which comprises Ala977 to Glu1038 of alpha-spectrin protein (SPTA1), is unknown [Lima et al., Blood 2005; 106 (11): 1666]. However, via SH3 domains, spectrin interacts with two members of the Ena/VASP (enabled/vasodilator-stimulated phosphoprotein) family: VASP and EVL (Ena/VASP-like). Ena/VASP proteins appear to regulate adhesion and to control actin dynamics [Machnicka et al. Cell. Mol. Life Sci. 2012; 69:191–201].

The pathogenic (class 5) intron 30 α-LEPRA (‘low expression allele PRAGUE’) variant was first described in trans to other *SPTA1* mutations in 1996 [Wichterle et al. J Clin Invest. 1996 Nov 15;98(10):2300-7]. It enhances an alternative acceptor splice site which ultimately activates nonsens-mediated decay leading to spectrin deficiency [Gallagher et al. J Clin Invest 2019 Apr 30;129(7):2878-2887]. This variant has been reported to have a minor allele frequency (MAF) of 0.004937 per gnomad.broadinstitute.org. The variant has been described in European [Wichterle et al. J Clin Invest. 1996 Nov 15;98(10):2300-7; Delaunay et al. Br J Haematol. 2004 Oct;127(1):118-22; van Vuren A et. al Hemasphere. 2019 Aug 7;3(4):e276] and North American (White, non-Hispanic > white, Hispanic) ancestries [Gallagher et al. J Clin Invest 2019 Apr 30;129(7):2878-2887; Tole S, et al. Br J Haematol. 2020 Nov;191(3):486-496; Chonat et al. Front Physiol. 2019 Jul 3;10:815]. To our knowledge, this variant was yet not reported in other ancestries.

The *ANK1* missense variant of unknown significance (class 3, VUS) has a high CADD score (i.e., 29.9) and is rare, but 14 heterozygote individuals are listed in gnomAD browser. The variant affects a R (Arginine in a coil sequence) in the ZU-5B domain, which is part of the spectrin-binding complex (composed of two ZU5 and the UPA domains).

The pathogenic compound heterozygous *SPTA1* variants α-LEPRA and p.Arg994Ter are the cause of AR inherited HS in patient P33 and her sister P69. Recently, Conat et al. proposed a three-scale phenotype classification of patients with causative *SPTA1* variants, and our patients would be considered Group II (severe to moderate, recessive HS) [Chonat et al. Front Physiol. 2019 Jul 3;10:815]. It is unknown, if the *ANK1* VUS affects the phenotype.


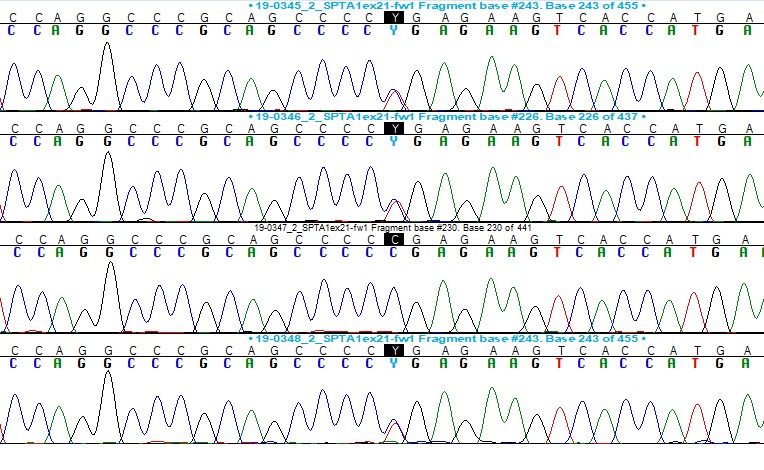


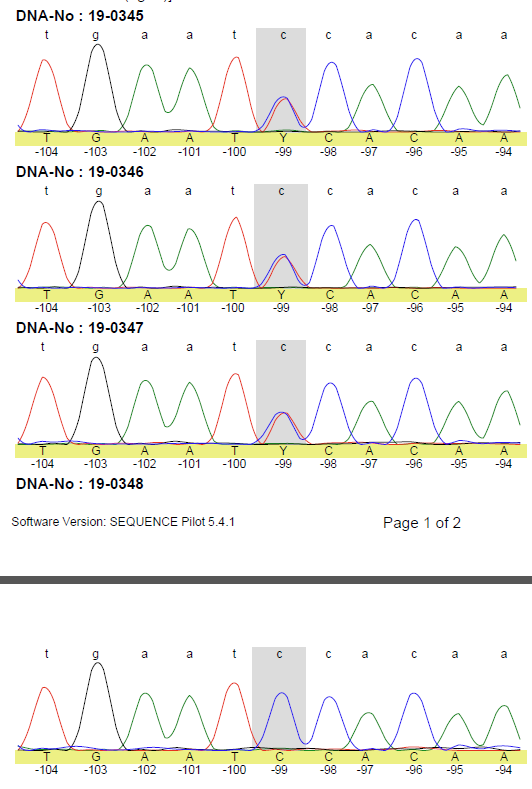

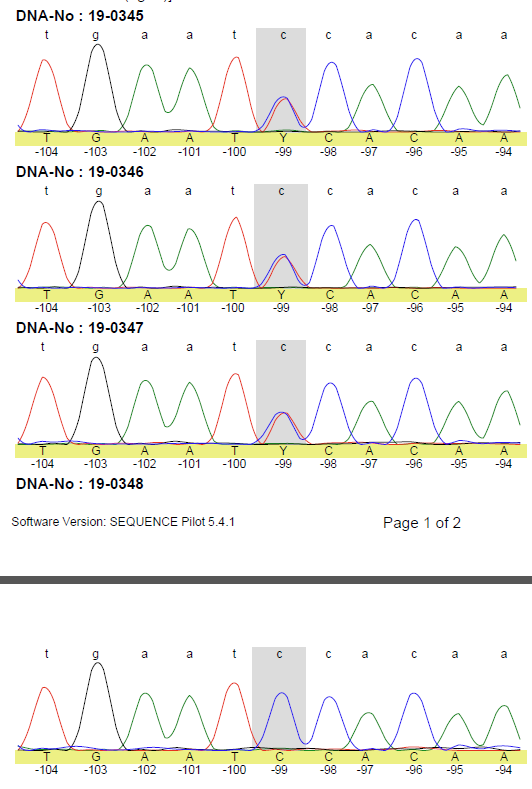


**Figure S21. Left:** *SPTA1* αLEPRA allele variant segregation. Starting from top to bottom of the chromatograms: Index patient (HET), affected sister (HET), mother (HET) and father (WT). **Right:** *SPTA1* nonsense variant segregation. Starting from top to bottom of the chromatograms: Index patient (HET), affected sister (HET), mother (WT) and father (HET).

**Family F34: Index group patient P34**, male, 11-years-old, Central European, moderate HS phenotype

| **GENE** | **CHR** | **POS** | **IDS** | **REF** | **ALT** | **gnomAD _AF** | **gnomAD _AC** | **gnomAD _hom** | **Consequence** | **HGVSc (cDNA)** | **HGVSp**  **(protein)** | **READS** | **SIFT** | **PolyPhen** | **CADD** |
| --- | --- | --- | --- | --- | --- | --- | --- | --- | --- | --- | --- | --- | --- | --- | --- |
| *SPTA1* | 1 | 158647479 | . | C | T | 0 | 0 | 0 | splice_donor | ENST00000368147.4:  c.957+1G>A | NA | 482 | NA | NA | 26.4 |
| *SPTA1* | 1 | 158613314 | . | G | A | 0.004937 | 691 | 3 | Intron | ENST00000368147.4:  c.4339-99C>T | ENSP00000357129.4:  p.Lys1449ValfsTer27 | 46 | NA | NA | 8.7 |

| **Method** | **LELY** | **αLEPRA** | **PMID** | **Pathogenicity (split)** |
| --- | --- | --- | --- | --- |
| Panel | Heterozygous | Heterozygous | - | PVS1+PM2+PP3 |
| Panel |  |  | 8941647 | PS1+PS3+PS4+PM3+PP1+PP3+PP4 |

Interpretation: P34 has a moderate HS phenotype and developed gall stones at age 7 years and underwent cholecystectomy and splenectomy at 8 years (Table S1d). He carries compound heterozygote pathogenic variants in *SPTA1*; that is two maternally inherited *SPTA1* variants (LELY and a class 5 pathogenic c.957+1G>A splice donor variant) and a paternally inherited pathogenic *SPTA1* αLEPRA allele. Of note, Aggarwal et al reported a similar intronic *SPTA1* splicing error variant (i.e., c.9581G>A) in combination with a *SPTA1* c.6536_6539del ACTT variant as cause of HS in two patients from India [Aggarwal et al. Br J Haematol. 2020 Mar;188(5):784-795].

The pathogenic compound heterozygous *SPTA1* variants α-LEPRA and c.957+1G>A are the cause of AR inherited HS in patient P34. Recently, Conat et al. proposed a three-scale phenotype classification of patients with causative SPTA1 variants, and our patients would be considered Group II (severe to moderate, recessive HS) [Chonat et al. Front Physiol. 2019 Jul 3;10:815].

**Summary *SPTA1 v*ariants**

The 3 patients (two index patients and one affected family member; due to the low number we herein include all affected patients) with causative *SPTA1* variants had, at least, one high impact variant – that is, a stop gain (N=2 patients) or a splicing variant (N=1 patient) (Figure S22). The other variant in trans was the same in all three patients, that is a deep intronic low expression variant called αLEPRA.


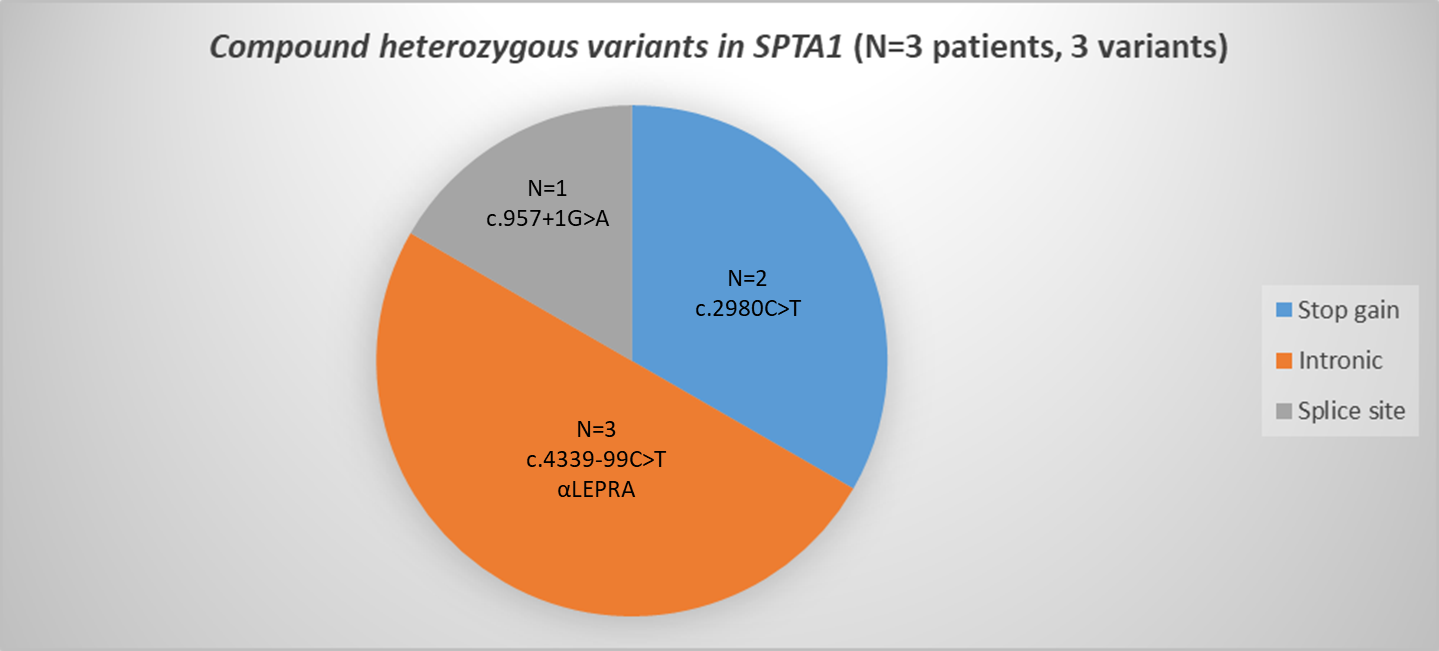


**Figure S22:** The 3 causative variants and the types of variation in *SPTA1* identified in three patients. Stop-gain, N=2 (P33 and her sister P69); Splicing, N=1 (P34); Intronic, N=3 (P33, P34 and P69). According to the annotation of pathogenicity based on the Standard Guidelines for the Interpretation of Sequence Variants [(https://pubmed.ncbi.nlm.nih.gov/25741868/)](https://pubmed.ncbi.nlm.nih.gov/25741868/), all three variants were classified as pathogenic.

**Figure S23A:** gDNA representation of *SPTA1*. In red, newly described variants. In black, variants previously reported in the literature. The colours of the circles are described in the CADD vs MAF plot. The effect on the protein level is displayed in main figure 1.


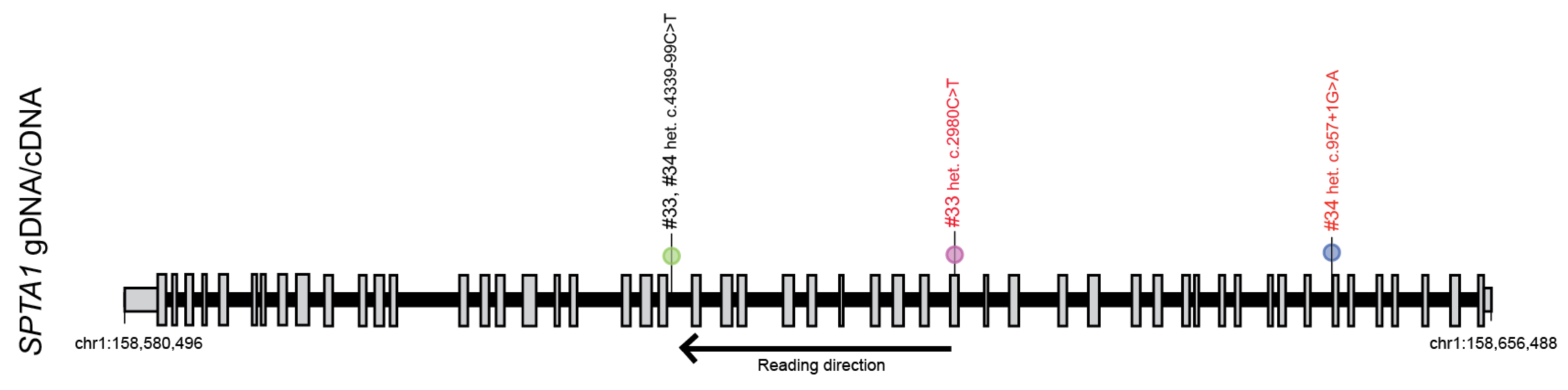


**Figure S23B:** CADD versus minor allele frequency (MAF) plot of all known *SPTA1* sequence variants visualized by PopViz [Zhang et al. Bioinformatics 2018; 34: 4307–4309]. The horizontal axis shows the MAF scores and the vertical axis the CADD v1.3 ones. The specific types of the various sequence variants, which were collected from the gnomAD r2.0.2 database (https://gnomad.broadinstitute.org/), are colour-coded, and the variants of our patients are square-shaped. CNVs are not included in the CADD/MAF plot.


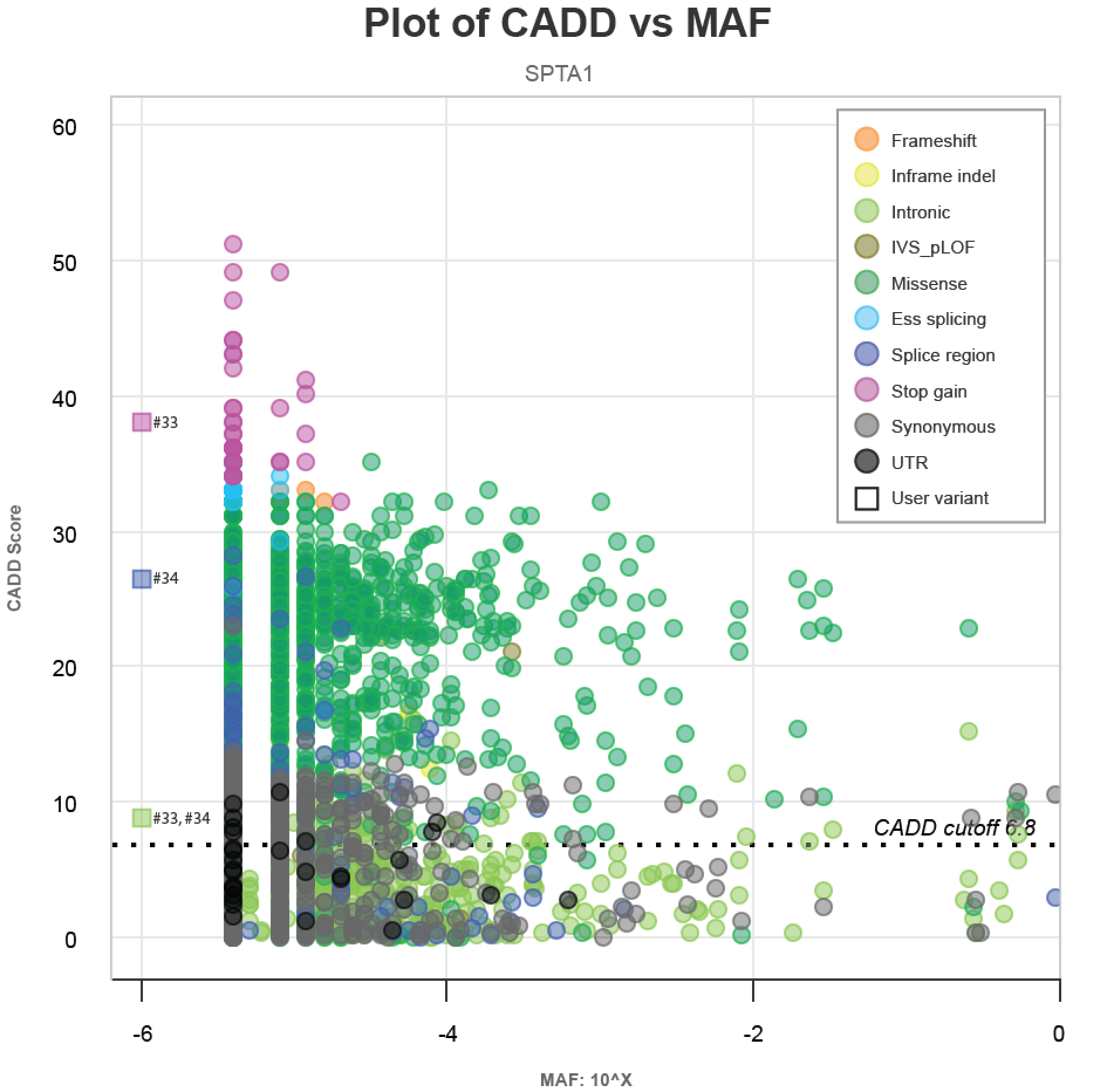


**Index patient (P) and his family (F) with no unequivocal causative candidate variants identified**

**Family F35: Index group patient P35**, male, 18-years-old, Central European, moderate HS phenotype

| **GENE** | **CHR** | **POS** | **IDS** | **REF** | **ALT** | **gnomAD _AF** | **gnomAD _AC** | **gnomAD _hom** | **Consequence** | **HGVSc (cDNA)** | **HGVSp (protein)** | **READS** | **SIFT** | **PolyPhen** | **CADD** |
| --- | --- | --- | --- | --- | --- | --- | --- | --- | --- | --- | --- | --- | --- | --- | --- |
| *ANK1* | 8 | 41573232 | . | C | A | 0,00008 | 19 | 0 | Missense | ENST00000289734.7:  c.1540G>T | ENSP00000289734.7:  p.Gly514Cys | 188 | Dele- terious | Probably _damaging | 31.0 |

| **Method** | **LELY** | **αLEPRA** | **PMID** | **Pathogenicity (split)** |
| --- | --- | --- | --- | --- |
| WES | Heterozygous | Wild type | - | PP3 |

Interpretation: P35 has a moderate HS and developed gall stones at the age of 4 years and underwent cholecystectomy at 14 years and splenectomy at 17 years (Table S1d). He carries an *ANK1* missense variant c.1540G>T, which is classified as variant of unknown significance (class 3, VUS). In addition, he carries a heterozygous *LELY* variant. The variants (*ANK1* and *LELY*) were identified via WES. His father, who has no HS phenotype (laboratory data not shown) carries the same *ANK1* variant, but is *LELY* wt. His mother, who has no HS phenotype (laboratory data not shown), is *ANK1* wt and carries the heterozygote *LELY* variant (Figure S24).

The *ANK1* missense VUS has a high CADD score (i.e., 31.0) and affects a highly conserved G (Glycine in a coil sequence) in AR16 of the membrane binding domain of ANK1. AR16-18 bind to band 3-III [Vallese et al. Nat Struct Mol Biol. 2022 Jul;29(7):706-718].

In addition, the patient has **α-Thalassemia minor** (heterozygote a^3,7^), the subclinical hemolysis of which, may have had contributed to the high bilirubin levels (median 100µmol/L) and early development of gall stones.

It is unknown if the combination of heterozygote *LELY* with *ANK1* c.1540G>T can explain the HS phenotype in P35; and further investigations are necessary to identify the underlying molecular cause of his disease.


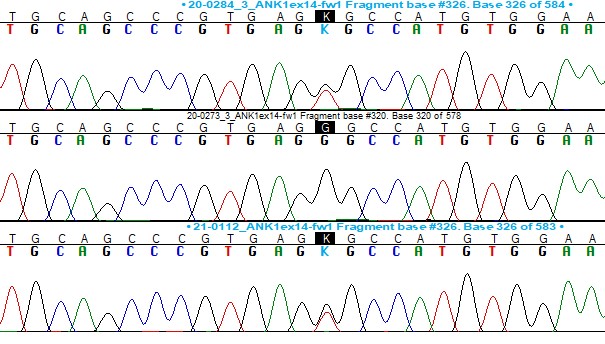

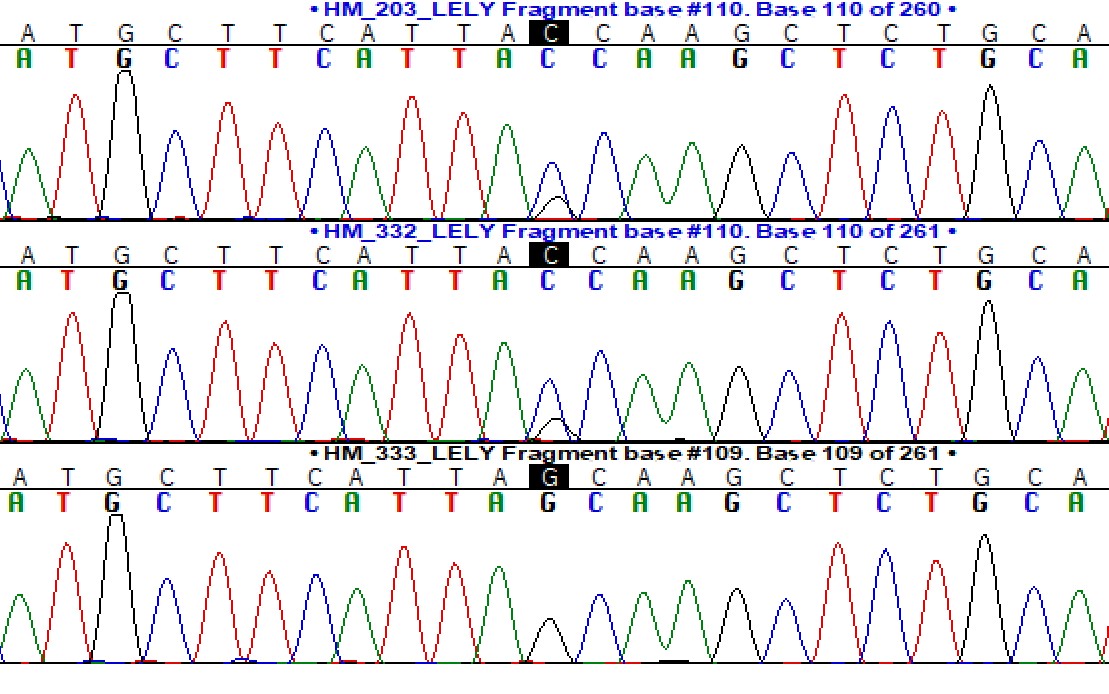


**Figure S24.** *ANK1 mis*sense variant segregation (left) and *LELY* allele segregation (right). Starting from top to bottom of the chromatograms for both variants, *ANK1* and *LELY*, respectively: Index patient (HET, HET), mother (WT, HET) and father (HET, WT).

**Summary of identified pathogenic variants in HS candidate genes in the index group**

In 34/35 patients in the index group, 34 causative variants in HS candidate genes have been identified. Variants in *ANK1* (46%) and *SPTB* (31%) were most commonly encountered. 14% had causative variants in *SCL4A1*, 6% had compound heterozygous variants in *SPTA1*, and in one patient (3%) no unequivocally proven causative variants had been identified (Figure S25).


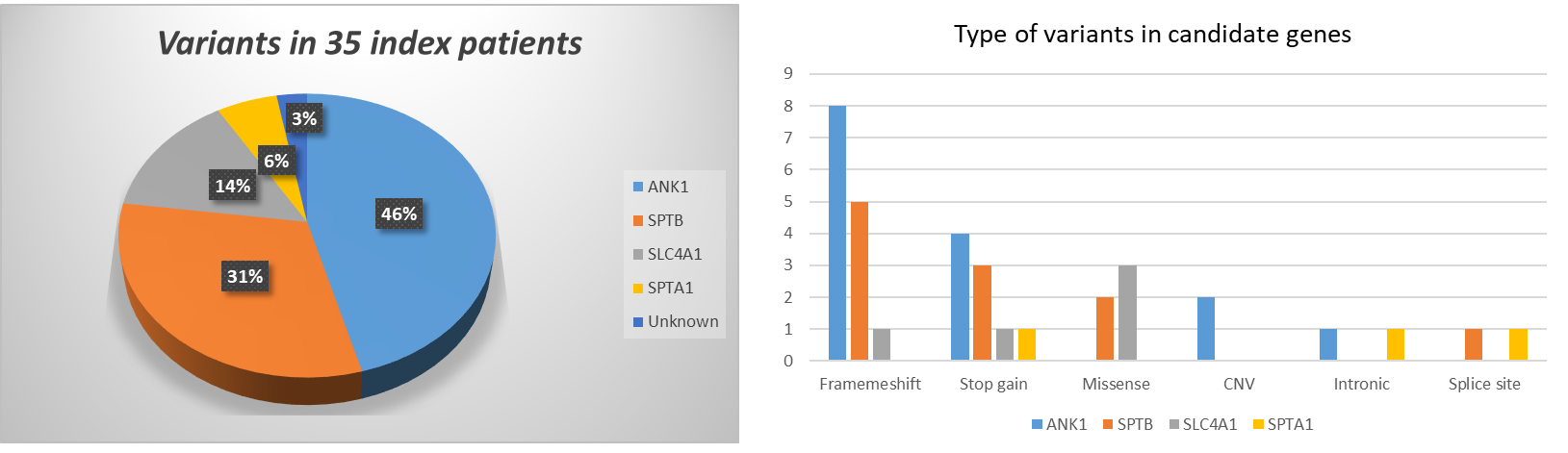


**Figure S25.** Distribution of causative variants among HS candidate genes in the index group (N=35 patients) (left). Type of variants and their distribution among candidate genes within the index group (right).

.

**Supporting References**

Aggarwal A, Jamwal M, Sharma P, Sachdeva MUS, Bansal D, Malhotra P, Das R. Deciphering molecular heterogeneity of Indian families with hereditary spherocytosis using targeted next-generation sequencing: First South Asian study. Br J Haematol. 2020 Mar;188(5):784-795.

Alanentalo T, Chatonnet F, Karlen M, Sulniute R, Ericson J, Andersson E, Ahlgren U. Cloning and analysis of Nkx6.3 during CNS and gastrointestinal development. Gene Expr Patterns. 2006 Jan;6(2):162-70.

An X, Debnath G, Guo X, Liu S, Lux SE, Baines A, Gratzer W, Mohandas N. Identification and functional characterization of protein 4.1R and actinbinding sites in erythrocyte beta spectrin: regulation of the interactions by phosphatidylinositol-4,5-bisphosphate. Biochemistry. 2005 Aug 9;44(31):10681-8.

Andres O, Loewecke F, Morbach H, Kraus S, Einsele H, Eber S, Speer CP. Hereditary spherocytosis is associated with decreased pyruvate kinase activity due to impaired structural integrity of the red blood cell membrane. Br J Haematol. 2019 Nov;187(3):386-395.

Choi HS, Choi Q, Kim JA, Im KO, Park SN, Park Y, Shin HY, Kang HJ, Kook H, Kim SY, Kim SJ, Kim I, Kim JY, Kim H, Park KD, Park KB, Park M, Park SK, Park ES, Park JA, Park JE, Park JK, Baek HJ, Seo JH, Shim YJ, Ahn HS, Yoo KH, Yoon HS, Won YW, Lee KS, Lee KC, Lee MJ, Lee

SA, Lee JA, Lee JM, Lee JH, Lee JW, Lim YT, Jung HJ, Chueh HW, Choi EJ, Jung HL, Kim JH, Lee DS; Hereditary Hemolytic Anemia Working Party of the Korean Society of Hematology. Molecular diagnosis of hereditary spherocytosis by multi-gene target sequencing in Korea: matching with osmotic fragility test and presence of spherocyte. Orphanet J Rare Dis. 2019 May 23;14(1):114.

Chonat S, Risinger M, Sakthivel H, Niss O, Rothman JA, Hsieh L, Chou ST, Kwiatkowski JL, Khandros E, Gorman MF, Wells DT, Maghathe T, Dagaonkar N, Seu KG, Zhang K, Zhang W, Kalfa TA. The Spectrum of SPTA1-Associated Hereditary Spherocytosis. Front Physiol. 2019 Jul 3;10:815.

Cunningham F, Allen JE, Allen J, Alvarez-Jarreta J, Amode MR, Armean IM et al. Ensembl 2022. Nucleic Acids Res 2022; 50: D988–D995.

Delaunay J, Nouyrigat V, Proust A, Schischmanoff PO, Cynober T, Yvart J, Gaillard C, Danos O, Tchernia G. Different impacts of alleles alphaLEPRA and alphaLELY as assessed versus a novel, virtually null allele of the SPTA1 gene in trans. Br J Haematol. 2004 Oct;127(1):118-22.

Dhermy D, Galand C, Bournier O, Boulanger L, Cynober T, Schismanoff PO, Bursaux E, Tchernia G, Boivin P, Garbarz M. Heterogenous band 3 deficiency in hereditary spherocytosis related to different band 3 gene defects. Br J Haematol. 1997 Jul;98(1):32-40.

Dhermy D, Burnier O, Bourgeois M, Grandchamp B. The red blood cell band 3 variant (band 3Biceêtrel:R490C) associated with dominant hereditary spherocytosis causes defective membrane targeting of the molecule and a dominant negative effect. Mol Membr Biol. 1999 Oct-Nov;16(4):305-12.

Eber S & Lux SE. Hereditary spherocytosis--defects in proteins that connect the membrane skeleton to the lipid bilayer. Semin Hematol. 2004 Apr;41(2):118-41.

Huang N, Lee I, Marcotte EM, Hurles ME. Characterising and predicting haploinsufficiency in the human genome. PLoS Genet 2010; 6: e1001154.

Ipsaro JJ, Huang L, Mondragón A. Structures of the spectrin-ankyrin interaction binding domains. Blood. 2009 May 28;113(22):5385-93.

Ipsaro JJ & Mondragón A. Structural basis for spectrin recognition by ankyrin. Blood. 2010 May 20;115(20):4093-101.

Jiang Y, Wang R, Urrutia E, Anastopoulos IN, Nathanson KL, Zhang NR. CODEX2: full-spectrum copy number variation detection by high-throughput DNA sequencing. Genome Biol 2018; 19: 202.

Kager L, Bruce LJ, Zeitlhofer P, Flatt JF, Maia TM, Ribeiro ML, Fahrner B, Fritsch G, Boztug K, Haas OA. Band 3 null^VIENNA^, a novel homozygous SLC4A1 p.Ser477X variant causing severe hemolytic anemia, dyserythropoiesis and complete distal renal tubular acidosis. Pediatr Blood Cancer. 2017 Mar;64(3).

Kager L, Jimenez Heredia R, Hirschmugl T, Dmytrus J, Krolo A, Müller H, Bock C, Zeitlhofer P, Dworzak M, Mann G, Holter W, Haas O, Boztug K. Targeted mutation screening of 292 candidate genes in 38 children with inborn haematological cytopenias efficiently identifies novel disease-causing mutations. Br J Haematol. 2018 Jul;182(2):251-258.

Kalli AC, Reithmeier RAF. Organization and Dynamics of the Red Blood Cell Band 3 Anion Exchanger SLC4A1: Insights From Molecular Dynamics Simulations. Front Physiol. 2022 Feb 25;13:817945.

King MJ, Behrens J, Rogers C, Flynn C, Greenwood D, Chambers K. Rapid flow cytometric test for the diagnosis of membrane cytoskeleton-associated haemolytic anaemia. Br J Haematol 2000; 111: 924–33.

King M-J, Garçon L, Hoyer JD, et al. ICSH guidelines for the laboratory diagnosis of nonimmune hereditary red cell membrane disorders. Int J Lab Hematol. 2015;37(3):304-325.

Kircher M, Witten DM, Jain P, O’Roak BJ, Cooper GM, Shendure J. A general framework for estimating the relative pathogenicity of human genetic variants. Nat Genet 2014; 46: 310–5.

Lawrence M, Gentleman R, Carey V. rtracklayer: an R package for interfacing with genome browsers. Bioinformatics 2009; 25: 1841–2.

Lawrence M, Morgan M. Scalable Genomics with R and Bioconductor. Stat Sci 2014; 29: 214–226.

Lazzareschi I, Curatola A, Pedicelli C, Castiglia D, Buonsenso D, Gatto A, Attinà G, Valentini P. A previously unrecognized Ankyrin-1 mutation associated with Hereditary Spherocytosis in an Italian family. Eur J Haematol. 2019 Nov;103(5):523-526.

Lek M, Karczewski KJ, Minikel E V, Samocha KE, Banks E, Fennell T et al. Analysis of protein-coding genetic variation in 60,706 humans. Nature 2016; 536: 285–91.

Lima PRM, Baratti MO, Basseres DS, Duarte ASS, Costa FF, Saad STO. The SH3 domain of alpha spectrin binds to galectin-1 during erythroid differentiation. Blood (2005) 106 (11): 1666.

Lin PC, Chiou SS, Lin CY, Wang SC, Huang HY, Chang YS, Tseng YH, Kan TM, Liao YM, Tsai SP, Peng CT, Chang JG. Whole-exome sequencing for the genetic diagnosis of congenital red blood cell membrane disorders in Taiwan. Clin Chim Acta. 2018 Dec;487:311-317.

Lunati-Rozie A, Janin A, Faubert E, Nony S, Renoux C, Carcao MD, Fanen P, Funalot B, Mansour-Hendili L, Joly P. Use of minigene assays as a useful tool to confirm the pathogenic role of intronic variations of the ANK1 gene: Report of two cases of hereditary spherocytosis. Br J Haematol. 2023 Mar 16. doi: 10.1111/bjh.18760. Online ahead of print.

Lux SE 4th. Anatomy of the red cell membrane skeleton: unanswered questions. Blood. 2016 Jan 14;127(2):187-99.

Machnicka B, Grochowalska R, Bogusławska DM, Sikorski AF, Lecomte MC. Spectrin-based skeleton as an actor in cell signaling. Cell. Mol. Life Sci. (2012) 69:191–201.

Matte A, Federti E, De Franceschi L. Erythrocyte pyruvate kinase activation in red cell disorders. Curr Opin Hematol. 2023 Feb 10.; doi: 10.1097/MOH.0000000000000758. Online ahead of print.

Miya K, Shimojima K, Sugawara M, Shimada S, Tsuri H, Harai-Tanaka T, Nakaoka S, Kanegane H, Miyawaki T, Yamamoto T. A de novo interstitial deletion of 8p11.2 including ANK1 identified in a patient with spherocytosis, psychomotor developmental delay, and distinctive facial features. Gene. 2012 Sep 10;506(1):146-9.

More TA, Devendra R, Dongerdiye R, Warang P, Kedar P. Targeted next-generation sequencing identifies novel deleterious variants in ANK1 gene causing severe hereditary spherocytosis in Indian patients: expanding the molecular and clinical spectrum. Mol Genet Genomics. 2023 Jan 4. doi: 10.1007/s00438-022-01984-1.

Müller H, Jimenez-Heredia R, Krolo A, Hirschmugl T, Dmytrus J, Boztug K et al. VCF.Filter: interactive prioritization of disease-linked genetic variants from sequencing data. Nucleic Acids Res 2017; 45: W567–W572.

Ozcan R, Jarolim P, Lux SE, Ungewickell E, Eber SW. Simultaneous (AC)n microsatellite polymorphism analysis and single-stranded conformation polymorphism screening is an efficient strategy for detecting ankyrin-1 mutations in dominant hereditary spherocytosis. Br J Haematol. 2003 Aug;122(4):669-77.

Ozen A, Comrie WA, Ardy RC, Domínguez Conde C, Dalgic B, Beser ÖF, Morawski AR, Karakoc-Aydiner E, Tutar E, Baris S, Ozcay F, Serwas NK, Zhang Y, Matthews HF, Pittaluga S, Folio LR, Unlusoy Aksu A, McElwee JJ, Krolo A, Kiykim A, Baris Z, Gulsan M, Ogulur I, Snapper SB, Houwen RHJ, Leavis HL, Ertem D, Kain R, Sari S, Erkan T, Su HC, Boztug K, Lenardo MJ. CD55 Deficiency, Early-Onset Protein-Losing Enteropathy, and Thrombosis. N Engl J Med. 2017 Jul 6;377(1):52-61.

Park J, Jeong DC, Yoo J, Jang W, Chae H, Kim J, Kwon A, Choi H, Lee JW, Chung NG, Kim M, Kim Y. Mutational characteristics of ANK1 and SPTB genes in hereditary spherocytosis. Clin Genet. 2016 Jul;90(1):69-78.

Plagnol V, Curtis J, Epstein M, Mok KY, Stebbings E, Grigoriadou S et al. A robust model for read count data in exome sequencing experiments and implications for copy number variant calling. Bioinformatics 2012; 28: 2747–54.

Qin L, Nie Y, Zhang H, Chen L, Zhang D, Lin Y, Ru K. Identification of new mutations in patients with hereditary spherocytosis by next-generation sequencing. J Hum Genet. 2020 Apr;65(4):427-434.

Ribeiro ML, Alloisio N, Almeida H, Gomes C, Texier P, Lemos C, Mimoso G, Morlé L, Bey-Cabet F, Rudigoz RC, Delaunay J, Tamagnini G. Severe hereditary spherocytosis and distal renal tubular acidosis associated with the total absence of band 3. Blood. 2000 Aug 15;96(4):1602-4.

Richards S, Aziz N, Bale S, Bick D, Das S, Gastier-Foster J, Grody WW, Hegde M, Lyon E, Spector E, Voelkerding K, Rehm HL; ACMG Laboratory Quality Assurance Committee. Standards and guidelines for the interpretation of sequence variants: a joint consensus recommendation of the American College of Medical Genetics and Genomics and the Association for Molecular Pathology. Genet Med. 2015 May;17(5):405-24.

Salzer E, Cagdas D, Hons M, Mace EM, Garncarz W, Petronczki ÖY, Platzer R, Pfajfer L, Bilic I, Ban SA, Willmann KL, Mukherjee M, Supper V, Hsu HT, Banerjee PP, Sinha P, McClanahan F, Zlabinger GJ, Pickl WF, Gribben JG, Stockinger H, Bennett KL, Huppa JB, Dupré L, Sanal Ö, Jäger U, Sixt M, Tezcan I, Orange JS, Boztug K. Nat Immunol. 2016 Dec;17(12):1352-1360.

Shen H, Huang H, Luo K, Yi Y, Shi X. Two different pathogenic gene mutations coexisted in the same hereditary spherocytosis family manifested with heterogeneous phenotypes. BMC Med Genet. 2019 May 24;20(1):90.

Shin S, Jang W, Kim M, Kim Y, Park SY, Park J, Yang YJ. Targeted next-generation sequencing identifies a novel nonsense mutation in SPTB for hereditary spherocytosis: A case report of a Korean family. Medicine (Baltimore). 2018 Jan;97(3):e9677.

Svidnicki MCCM, Zanetta GK, Congrains-Castillo A, Costa FF, Saad STO. Targeted next-generation sequencing identified novel mutations associated with hereditary anemias in Brazil. Ann Hematol. 2020 May;99(5):955-962.

Tole S, Dhir P, Pugi J, Drury LJ, Butchart S, Fantauzzi M, Langer JC, Baker JM, Blanchette VS, Kirby-Allen M, Carcao MD. Genotype-phenotype correlation in children with hereditary spherocytosis. Br J Haematol. 2020 Nov;191(3):486-496.

Vallese F, Kim K, Yen LY, Johnston JD, Noble AJ, Calì T, Clarke OB. Architecture of the human erythrocyte ankyrin-1 complex. Nat Struct Mol Biol. 2022 Jul;29(7):706-718.

van Vuren A, van der Zwaag B, Huisjes R, Lak N, Bierings M, Gerritsen E, van Beers E, Bartels M, van Wijk R. The Complexity of GenotypePhenotype Correlations in Hereditary Spherocytosis: A Cohort of 95 Patients: Genotype-Phenotype Correlation in Hereditary Spherocytosis. Hemasphere. 2019 Aug 7;3(4):e276.

Vercellati C, Marcello AP, Fattizzo B, Zaninoni A, Seresini A, Barcellini W, Bianchi P, Fermo E. Effect of primary lesions in cytoskeleton proteins on red cell membrane stability in patients with hereditary spherocytosis. Front Physiol. 2022 Aug 12;13:949044.

Wang R, Yang S, Xu M, Huang J, Liu H, Gu W, Zhang X. Exome sequencing confirms molecular diagnoses in 38 Chinese families with hereditary spherocytosis. Sci China Life Sci. 2018 Aug;61(8):947-953.

Wang D, Lai P. Global retardation and hereditary spherocytosis associated with a novel deletion of chromosome 8p11.21 encompassing KAT6A and ANK1. Eur J Med Genet. 2020 Dec;63(12):104082.

Wang D, Song L, Shen L, Zhang K, Lv Y, Gao M, Ma J, Wan Y, Gai Z, Liu Y. Mutational Characteristics of Causative Genes in Chinese Hereditary Spherocytosis Patients: a Report on Fourteen Cases and a Review of the Literature. Front Pharmacol. 2021 Jul 16;12:644352.

Wang WJ, Xie JD, Yao H, Ding ZX, Jiang AR, Ma L, Shen HJ, Chen SN. Identification of variants in 94 Chinese patients with hereditary spherocytosis by next-generation sequencing. Clin Genet. 2023 Jan;103(1):67-78.

H Wichterle, M Hanspal, J Palek, P Jarolim. Combination of two mutant alpha spectrin alleles underlies a severe spherocytic hemolytic anemia

J Clin Invest. 1996 Nov 15;98(10):2300-7.

Wilmotte R, Maréchal J, Morlé L, Baklouti F, Philippe N, Kastally R, Kotula L, Delaunay J, Alloisio N.J. Low expression allele alpha LELY of red cell spectrin is associated with mutations in exon 40 (alpha V/41 polymorphism) and intron 45 and with partial skipping of exon 46. J Clin Invest. 1993 May;91(5):2091-6.

Wu C, Xiong T, Xu Z, Zhan C, Chen F, Ye Y, Wang H, Yang Y. Preliminary Study on the Clinical and Genetic Characteristics of Hereditary Spherocytosis in 15 Chinese Children. Front Genet. 2021 Mar 18;12:652376. eCollection 2021.

Xia X, Liu S, Zhou ZH. Structure, dynamics and assembly of the ankyrin complex on human red blood cell membrane. Nat Struct Mol Biol. 2022

Jul;29(7):698-705

Xie F, Lei L, Cai B, Gan L, Gao Y, Liu X, Zhou L, Jiang J. Clinical manifestation and phenotypic analysis of novel gene mutation in 28 Chinese children with hereditary spherocytosis. Mol Genet Genomic Med. 2021 Apr;9(4):e1577.

Yamamoto KS, Utshigisawa T, Ogura H, Aoki T, Kawakami T, Ohga S, Ohara A, Ito E, Yamamoto T, Kanno H. Clinical and genetic diagnosis of thirteen Japanese patients with hereditary spherocytosis. Hum Genome Var. 2022 Jan 12;9(1):1.

Yasanuga M, Ipsaro JJ, Mondragon A. Structurally similar but functionally diverse ZU5 domains in human erythrocyte ankyrin. J Mol Biol. 2012 Apr 6;417(4):336-50.

Yang L, Shu H, Zhou M, Gong Y. Literature review on genotype-phenotype correlation in patients with hereditary spherocytosis. Clin Genet. 2022 Sep 7. doi: 10.1111/cge.14223.

Zhang P, Bigio B, Rapaport F, Zhang SY, Casanova JL, Abel L et al. PopViz: A webserver for visualizing minor allele frequencies and damage prediction scores of human genetic variations. Bioinformatics 2018; 34: 4307–4309.

Zhang Y, Shao S, Liu J, Zeng C, Han Y, Zhang X. Neonatal hereditary spherocytosis caused by a de novo frameshift mutation of the SPTB gene characterized by hydrops fetalis: A case report. Medicine (Baltimore). 2021 Mar 26;100(12):e24804.

1. Age at analysis. ^2^Patient P19 has autosomal recessive hereditary spherocytosis with homozygous *SLC4A1* variants associated with hereditary spherocytosis and distal renal acidosis [Kager et al. PBC 2016]. Abbreviations: CE, Central European; ERCP, endoscopic retrograde cholangiopancreatography; f, female, m, male

   [↑](#footnote-ref-2)
2. Age at analysis. ^2^P28 carries *UGT1A1* homozygous promotor variants (Gilbert Meulengracht disease). Abbreviations: CE, Central European; ERCP, endoscopic retrograde cholangiopancreatography; f, female; m, male

   [↑](#footnote-ref-3)
3. Age at analysis. Abbreviations: CE, Central European; ERCP, endoscopic retrograde cholangiopancreatography; f, female; m, male [↑](#footnote-ref-4)
4. Age at analysis. Abbreviations: CE, Central European; ERCP, endoscopic retrograde cholangiopancreatography; f, female; m, male [↑](#footnote-ref-5)
5. Age at analysis. ^2^Testing was performed in our hospital reference laboratory after splenectomy. Abbreviations: CE, Central European; f, female; m, male [↑](#footnote-ref-6)
6. Age at analysis. Abbreviations: CE, Central European; f, female. [↑](#footnote-ref-7)
7. According to the review of Yang et al. [Yang L, et al. Clin Genet. 2022 Sep 7. doi: 10.1111/cge.14223] three more patients with this variant have been reported, but we were unable to identify these patients in the English literature. Abbreviation: PMID, Pubmed ID [↑](#footnote-ref-8)
